# Supplementary figures and images for: Inference of Candidate Germline Mutator Loci in Humans from Genome-Wide Haplotype Data
Source: PLoS Genet. 2017 Jan 17;13(1):e1006549. doi: 10.1371/journal.pgen.1006549 (PMC5283766; doi:10.1371/journal.pgen.1006549)

1

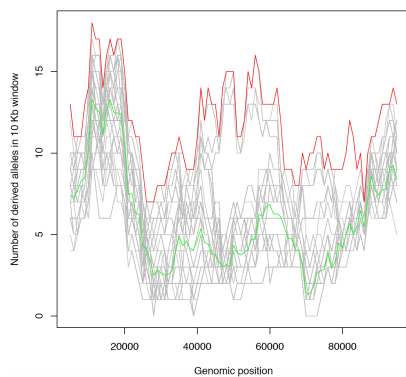

2

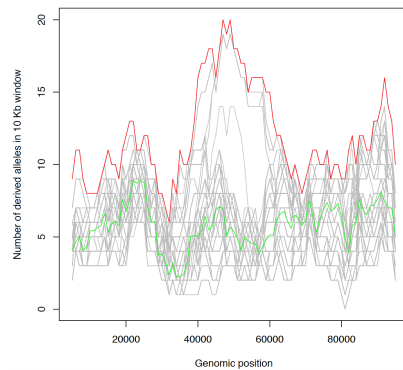

3

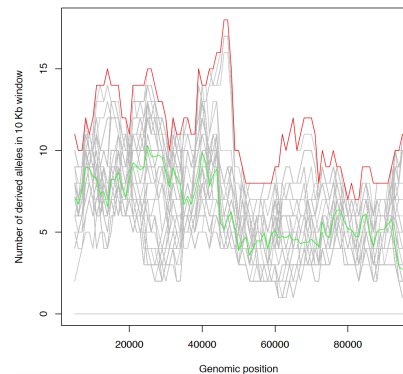

4

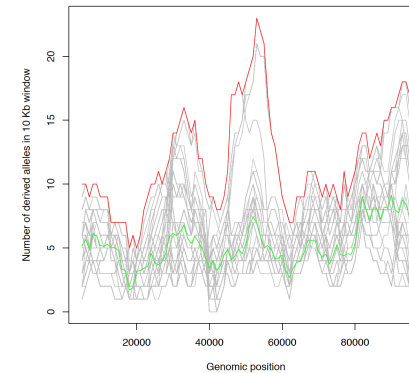

5

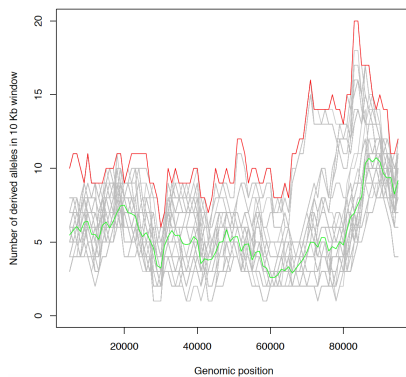

6

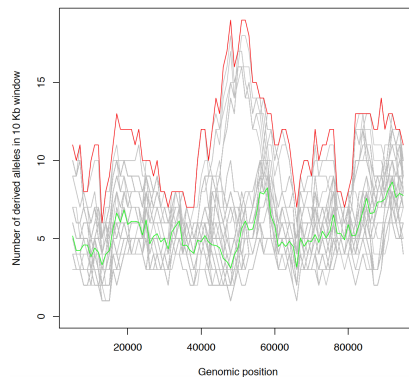

7

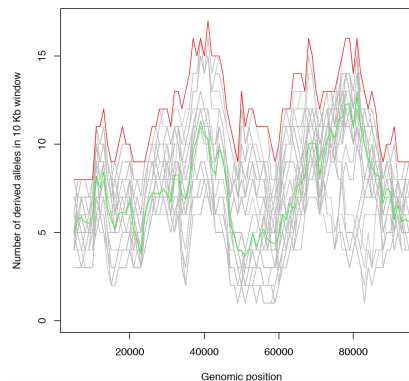

8

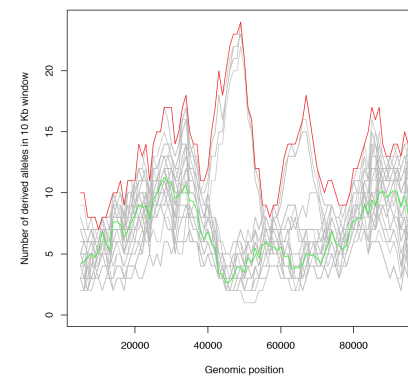

9

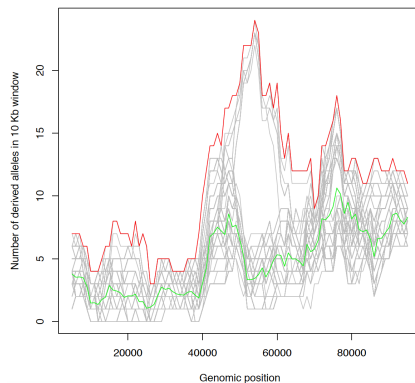

10

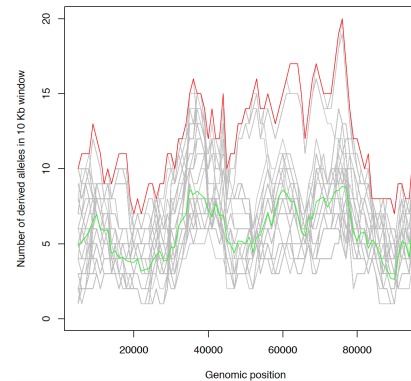

Supplement: S1 Fig — Ten random simulations, equivalent to the simulation shown in Fig 1. (PDF) [file pgen.1006549.s001.pdf]

A

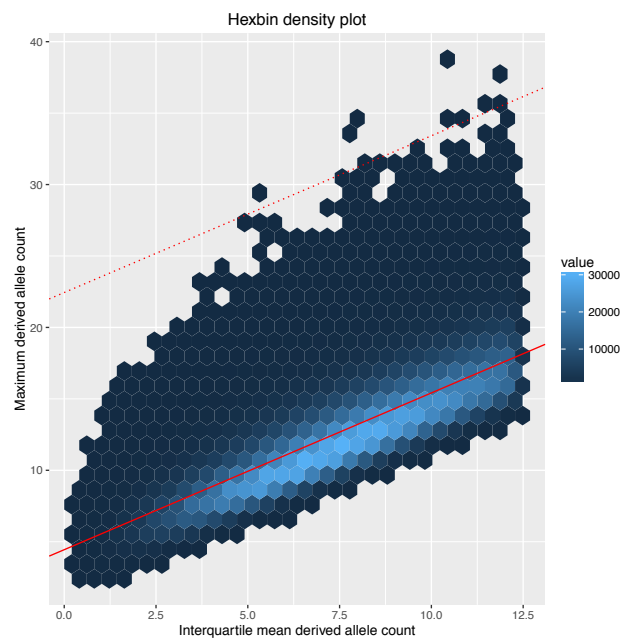

B

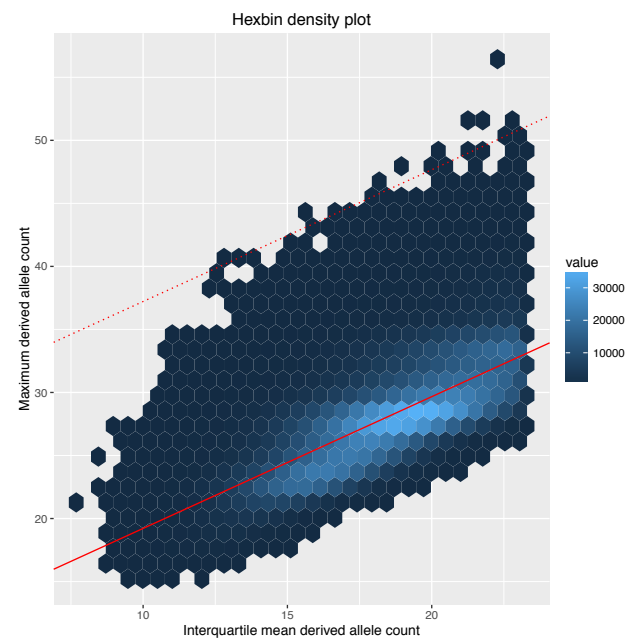

C

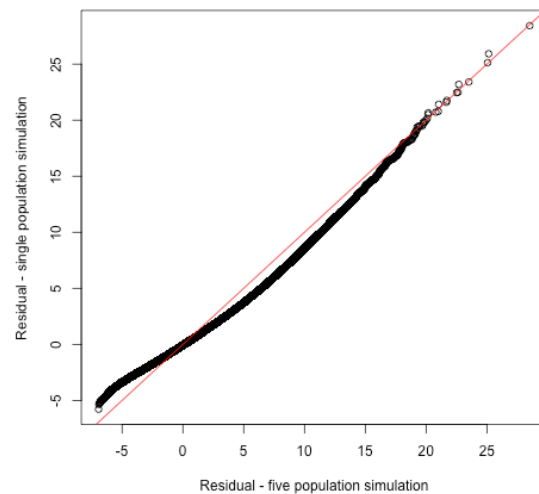

Supplement: S2 Fig — (a,b) Hexagon bin plots illustrating the relationship between the maximum and the interquartile mean number of derived alleles across all haplotypes in 10Kb windows for two simulated demographic scenarios, consisting of a single population (a) or five populations separated 100 kya (b). The shading indicates the number of points within each hexagonal bin. The linear regression line is shown as a solid red line and the dashed red line corresponds to the threshold for the candidate loci shown in Table 1. (c) Quantile-quantile plot of the residuals from the regression line in the single population and five-population simulations. (PDF) [file pgen.1006549.s002.pdf]

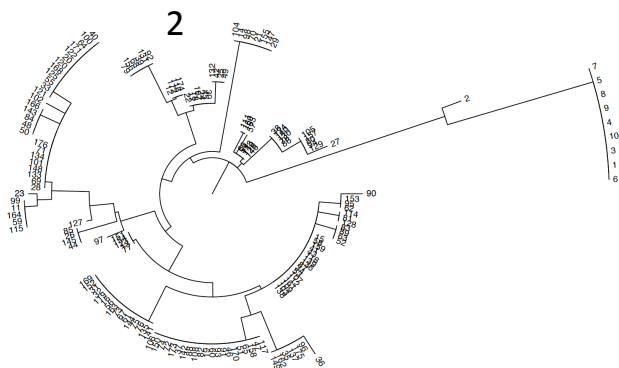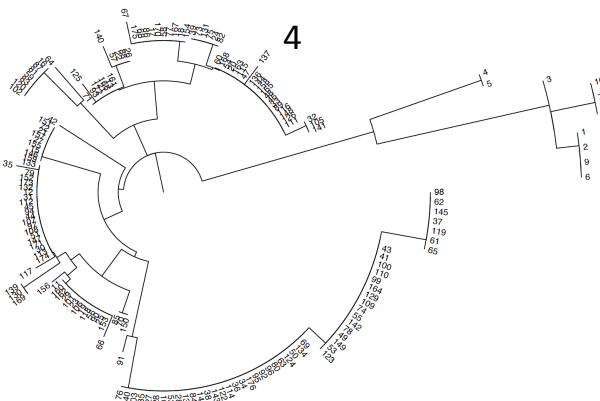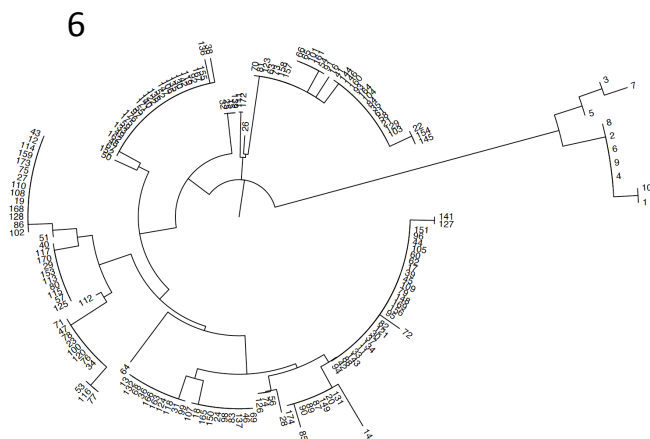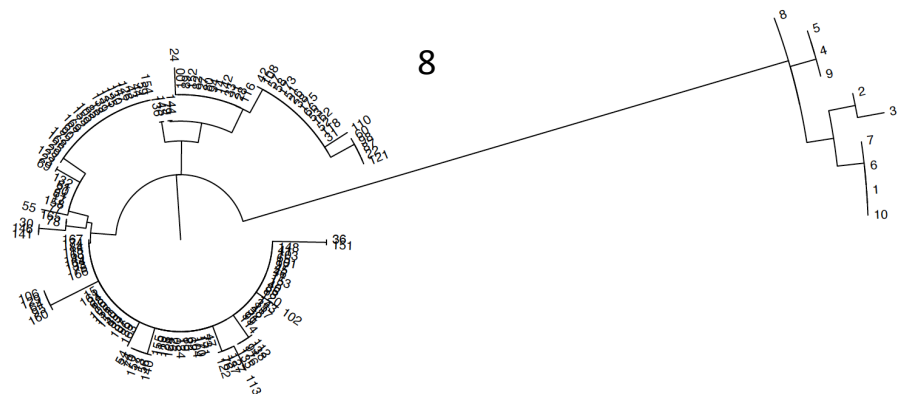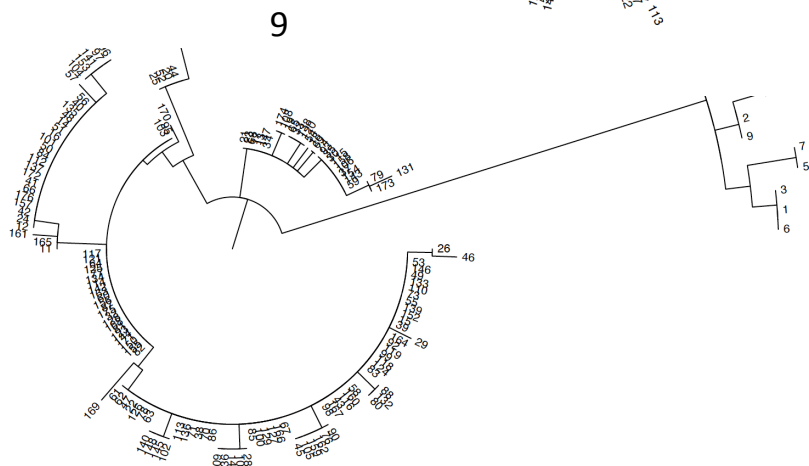

Supplement: S4 Fig — Trees from the subset of the ten proof-of-concept simulations that showed a peak in the maximal derived allele count. (PDF) [file pgen.1006549.s004.pdf]

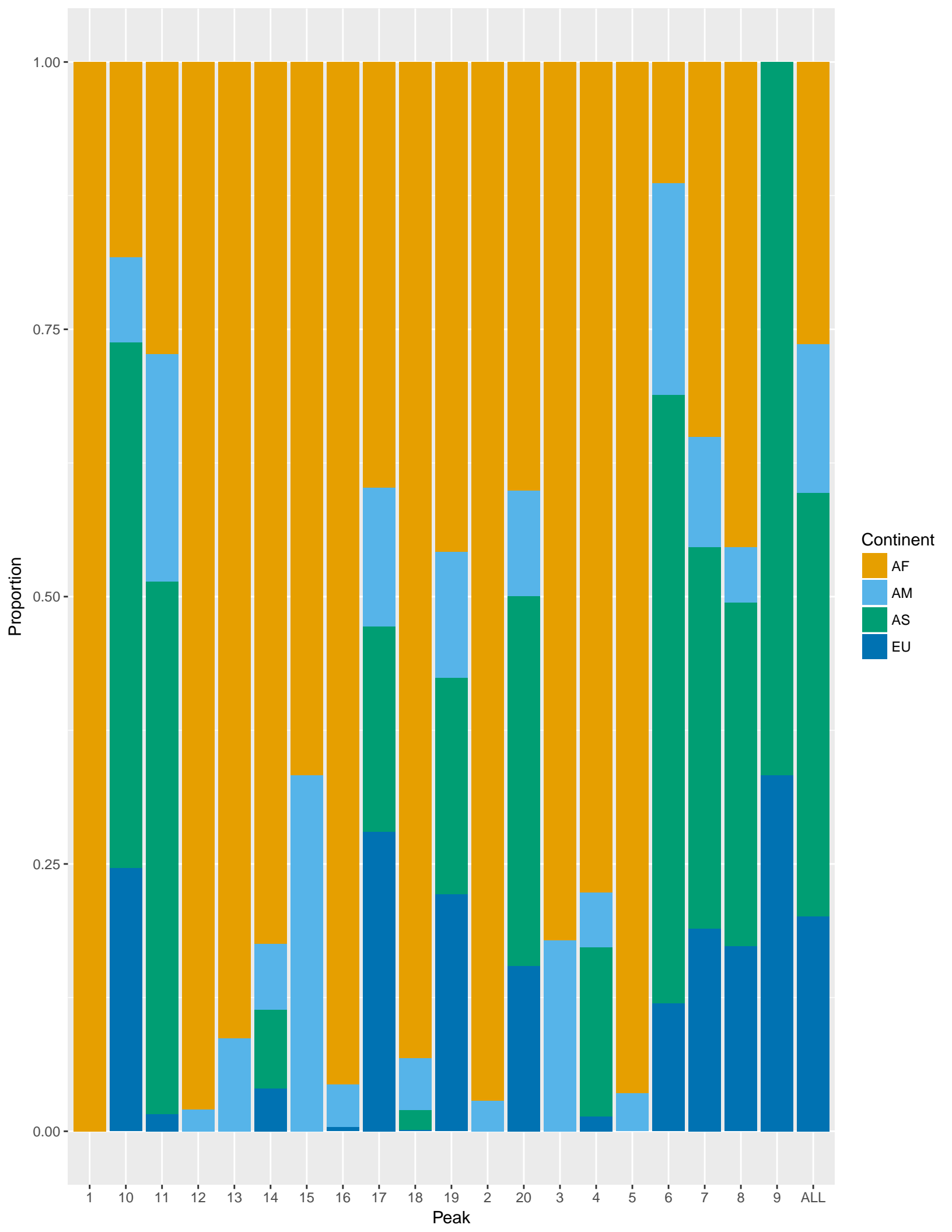

Supplement: S5 Fig — The proportion of the highly derived haplotypes at each peak corresponding to populations from Africa, Asia, America and Europe. The rightmost bar shows the proportions of each continent of origin among all G1K phase 3 samples. (PDF) [file pgen.1006549.s005.pdf]

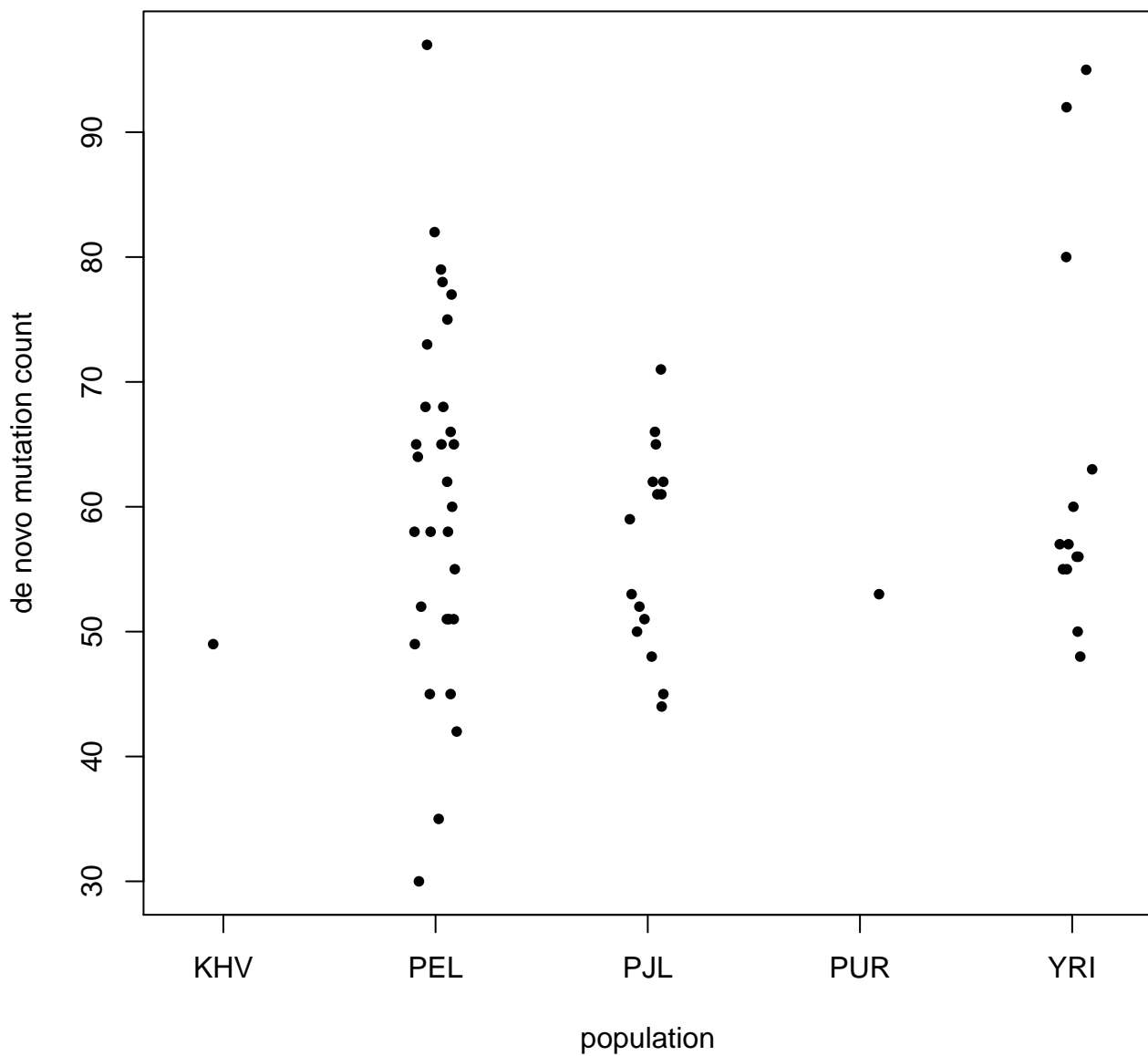

Supplement: S6 Fig — Stripchart showing de novo mutation count as a function of population of origin in the G1K data. (PDF) [file pgen.1006549.s006.pdf]

Chromosome 14

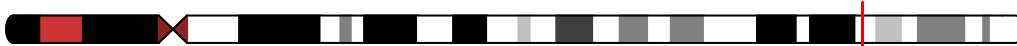

90.435 mb

90.445 mb

90.455 mb

90.44 mb

90.45 mb

Gene Models

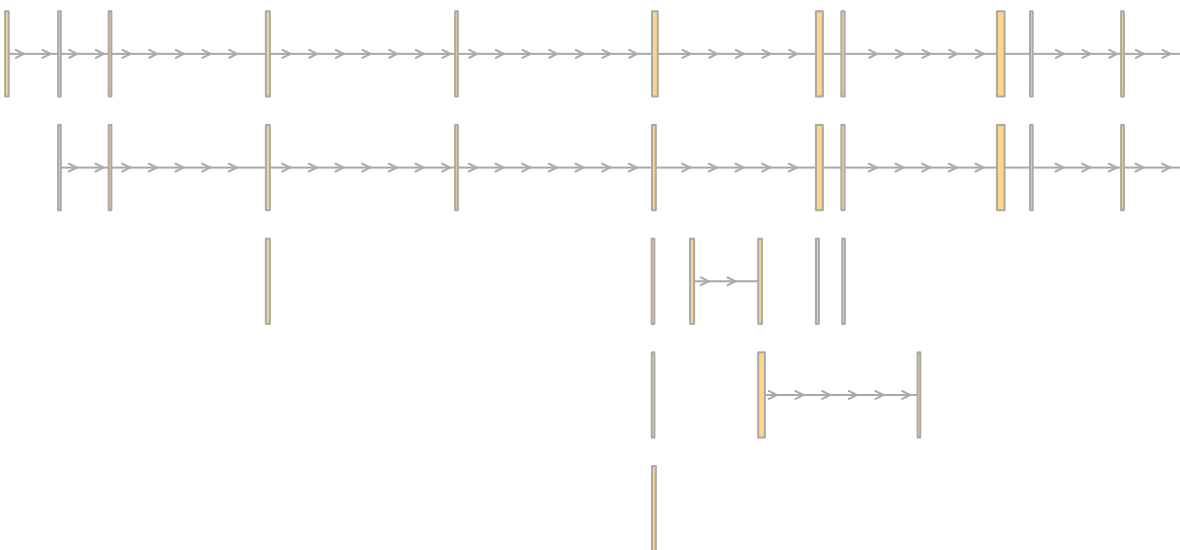

Residual

15

10

5

0

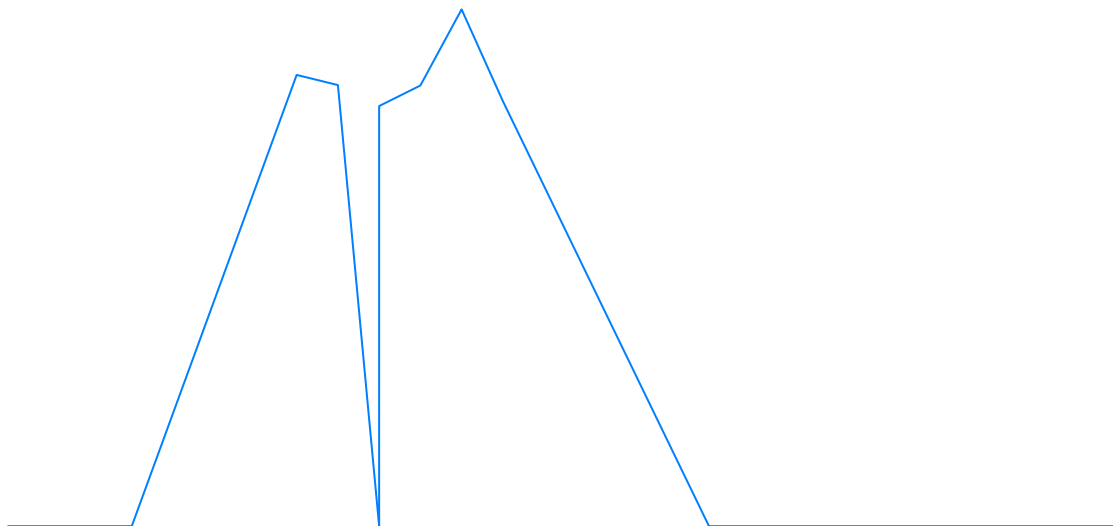

Supplement: S7 Fig — (PDF) [file pgen.1006549.s007.pdf]

10\_25031000

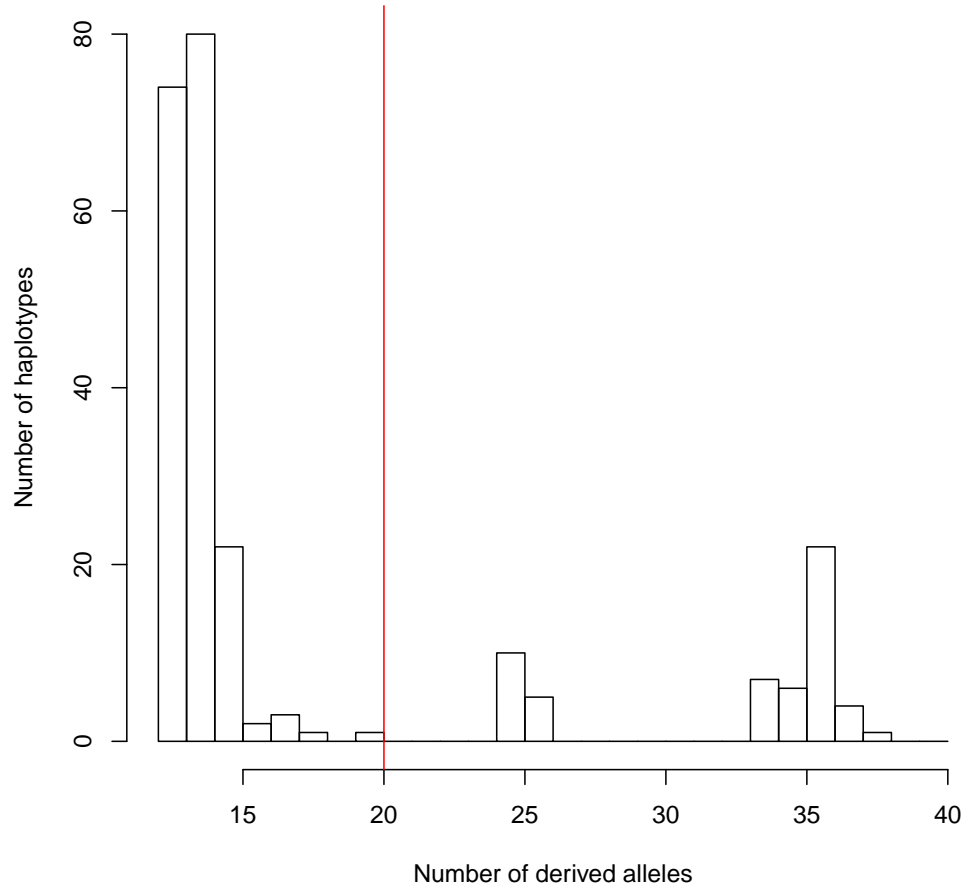

11\_1399000

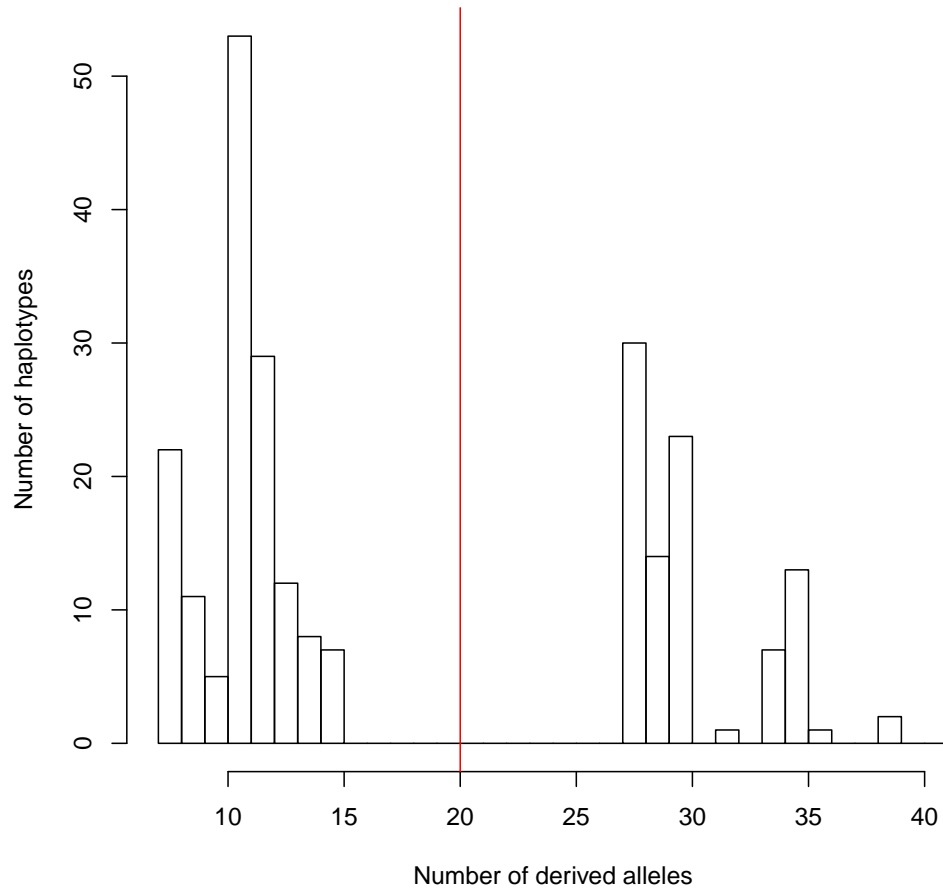

12\_44619000

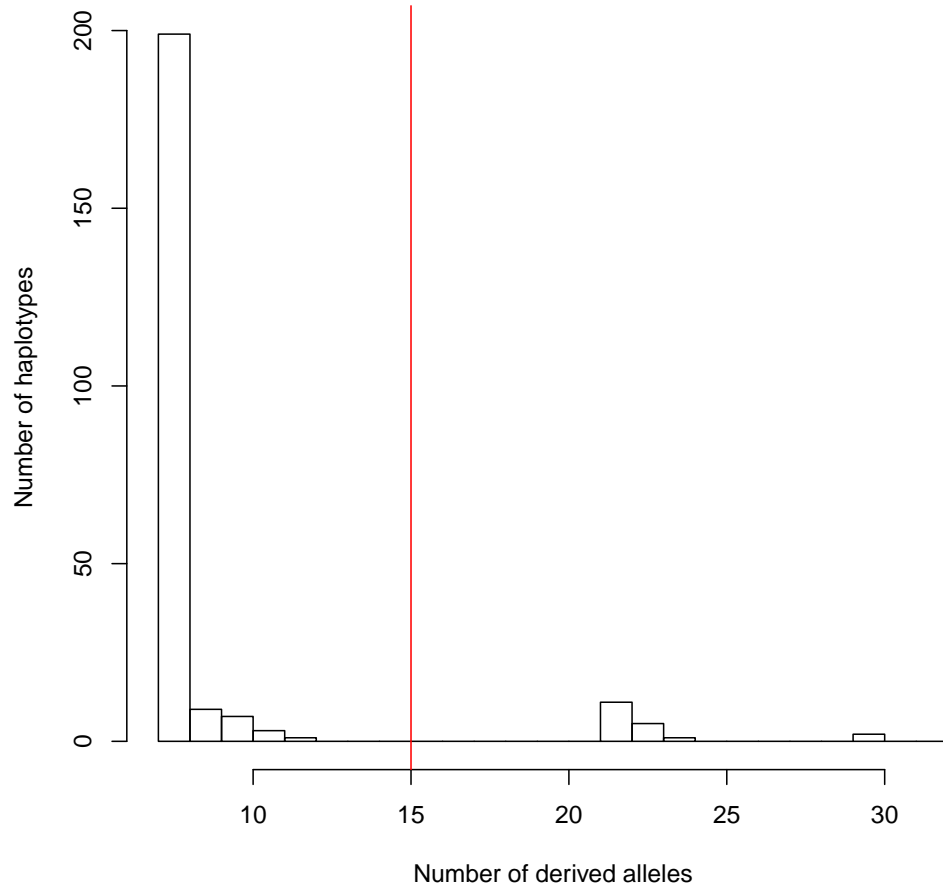

12\_87448000

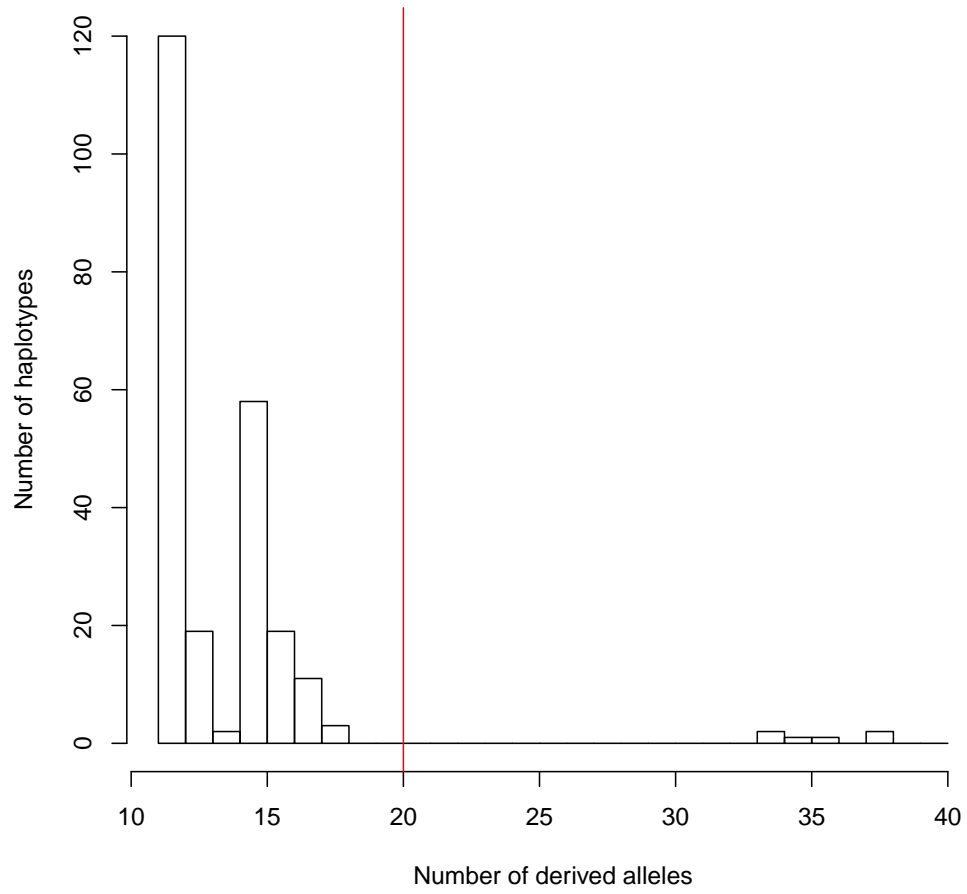

12\_96533000

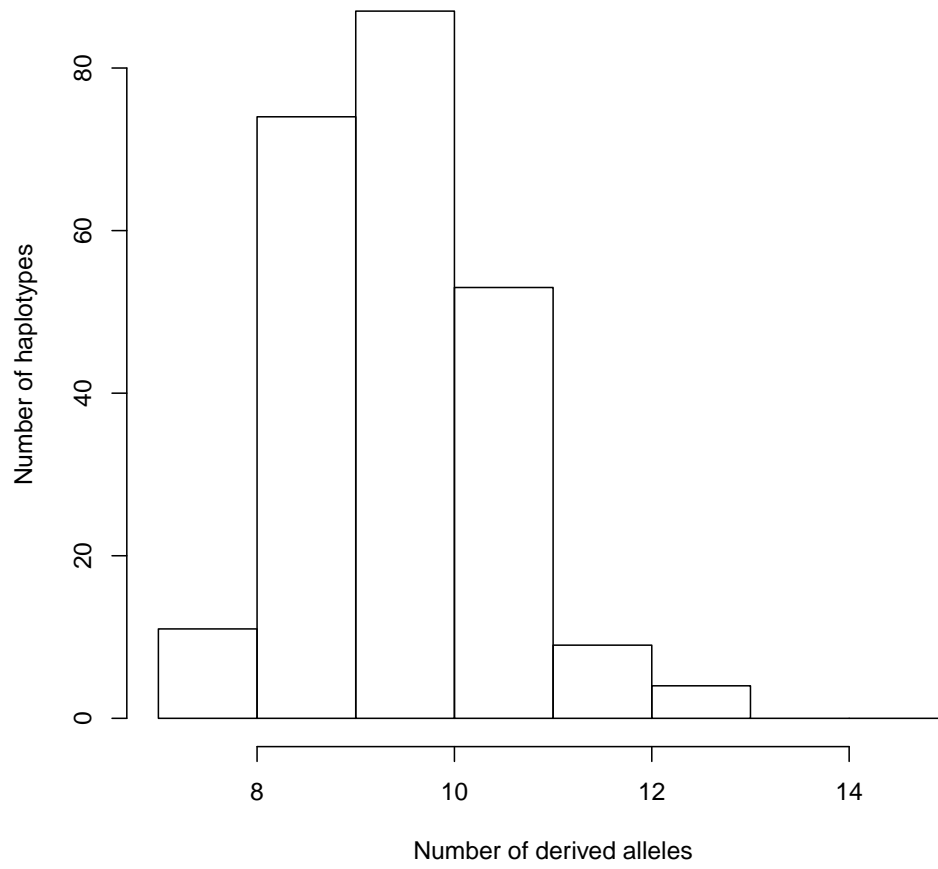

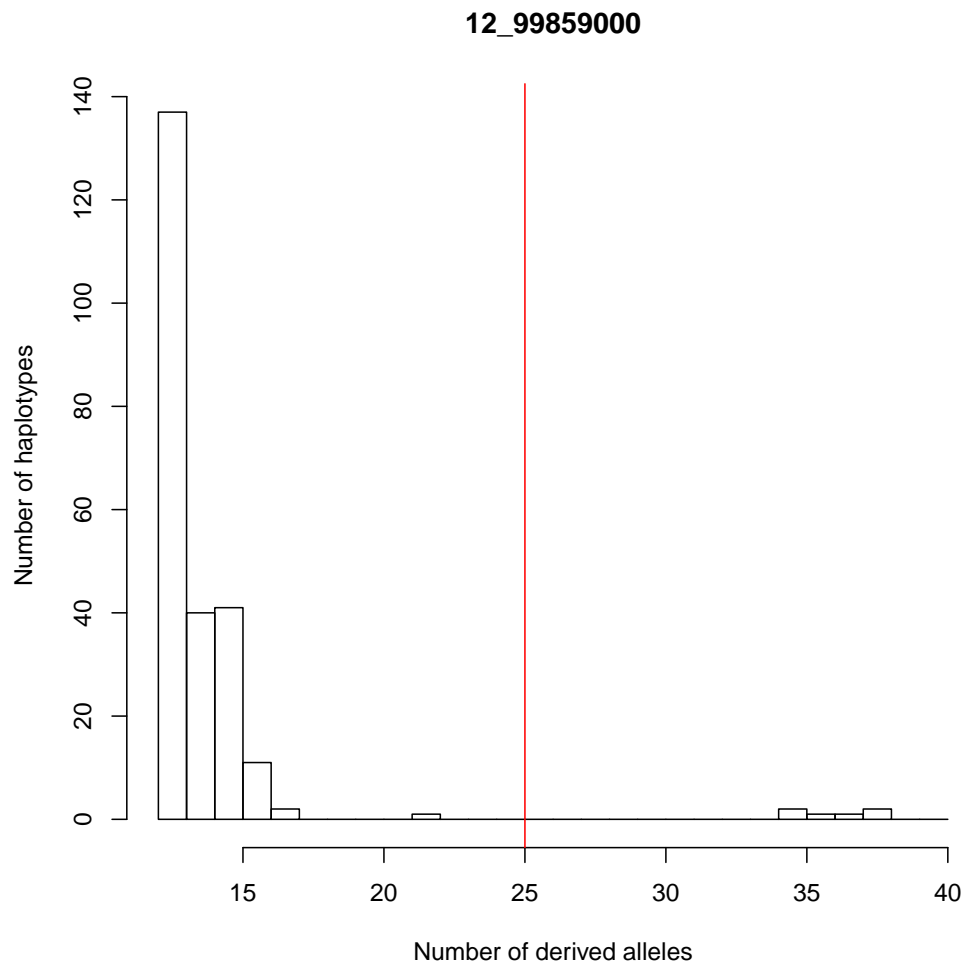

14\_90442000

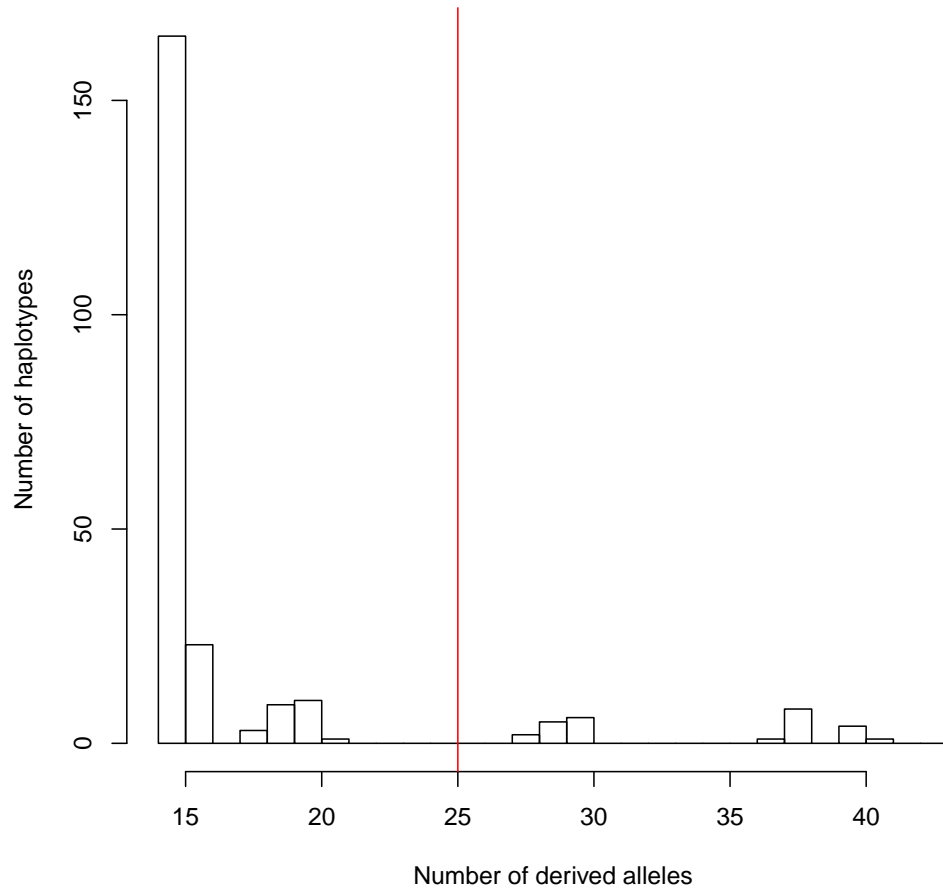

16\_32379000

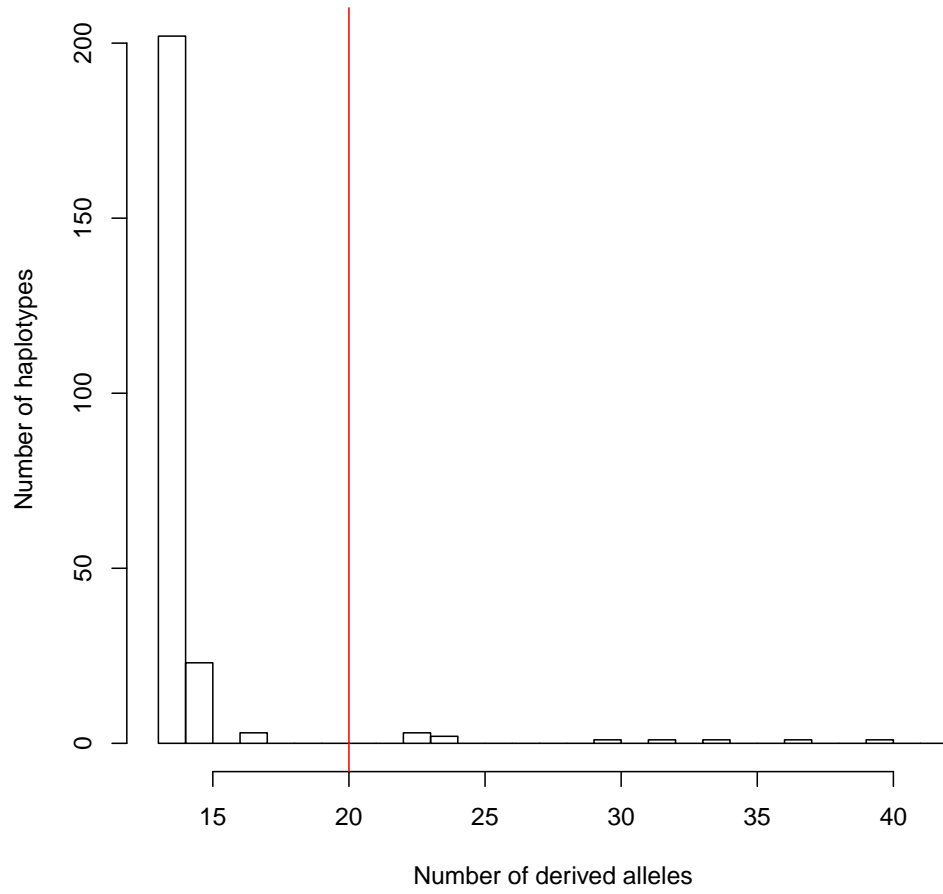

17\_15866000

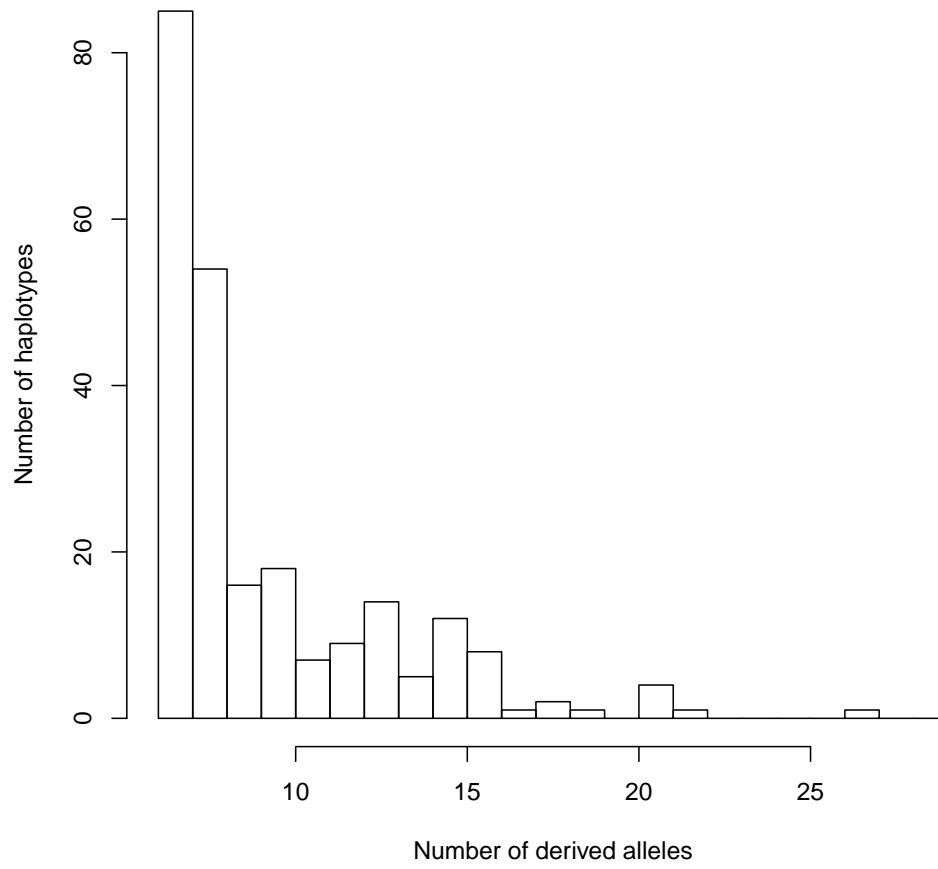

**18\_15301000**

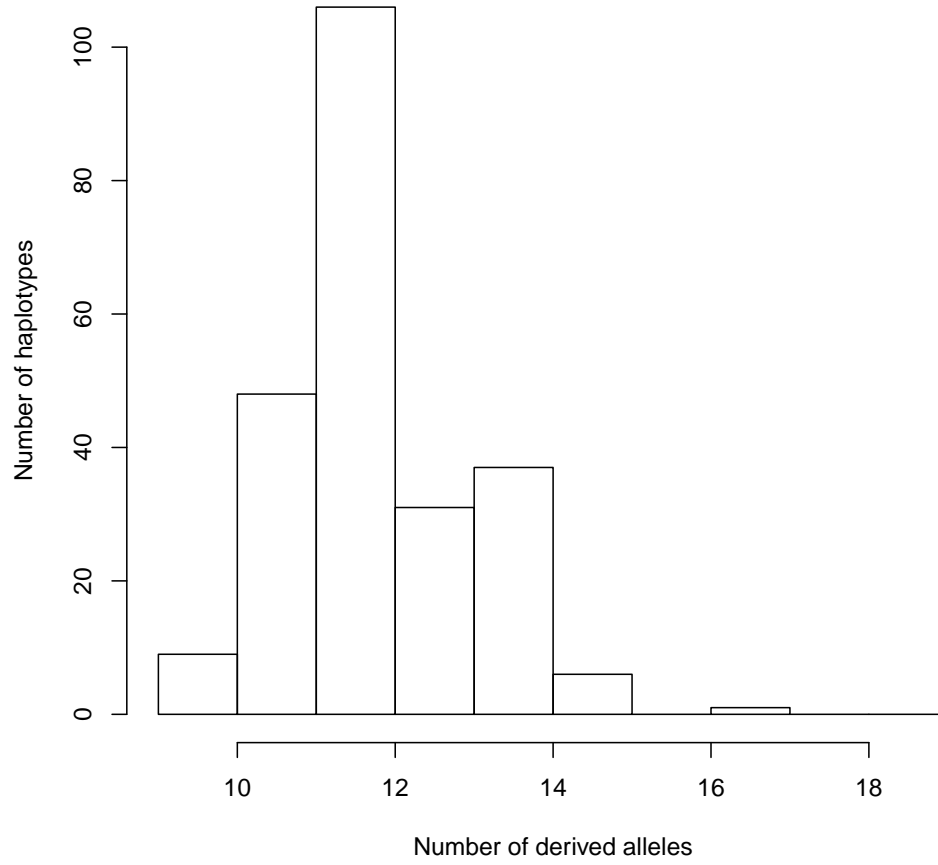

**18\_24477000**

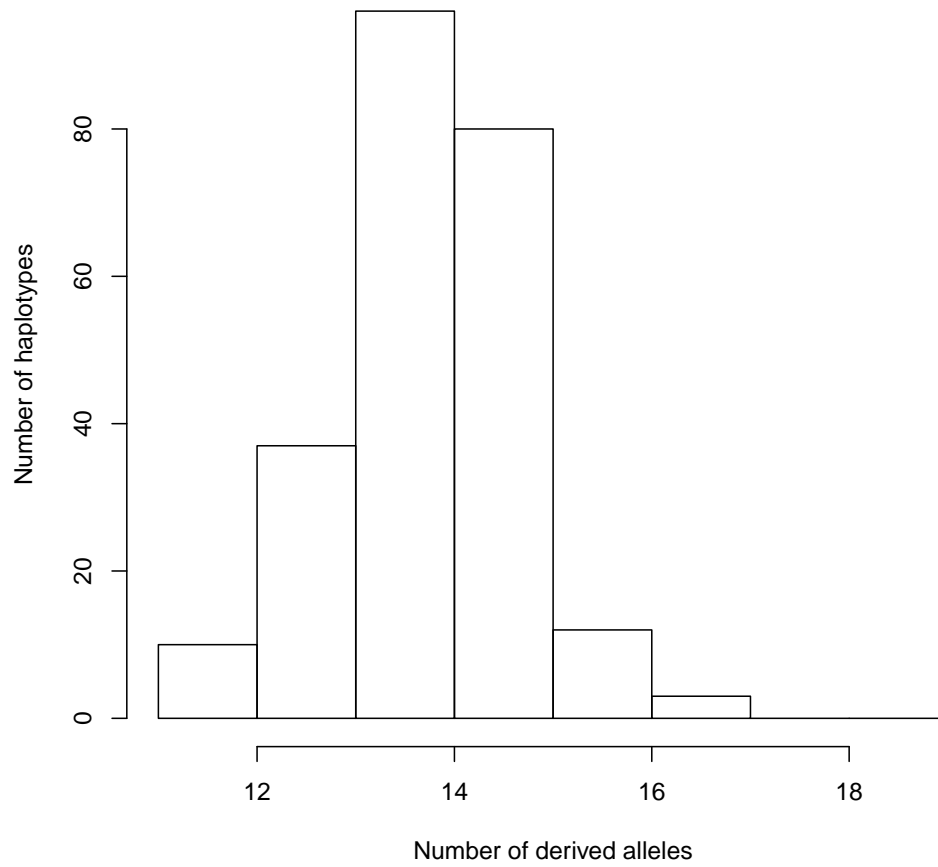

1\_192611000

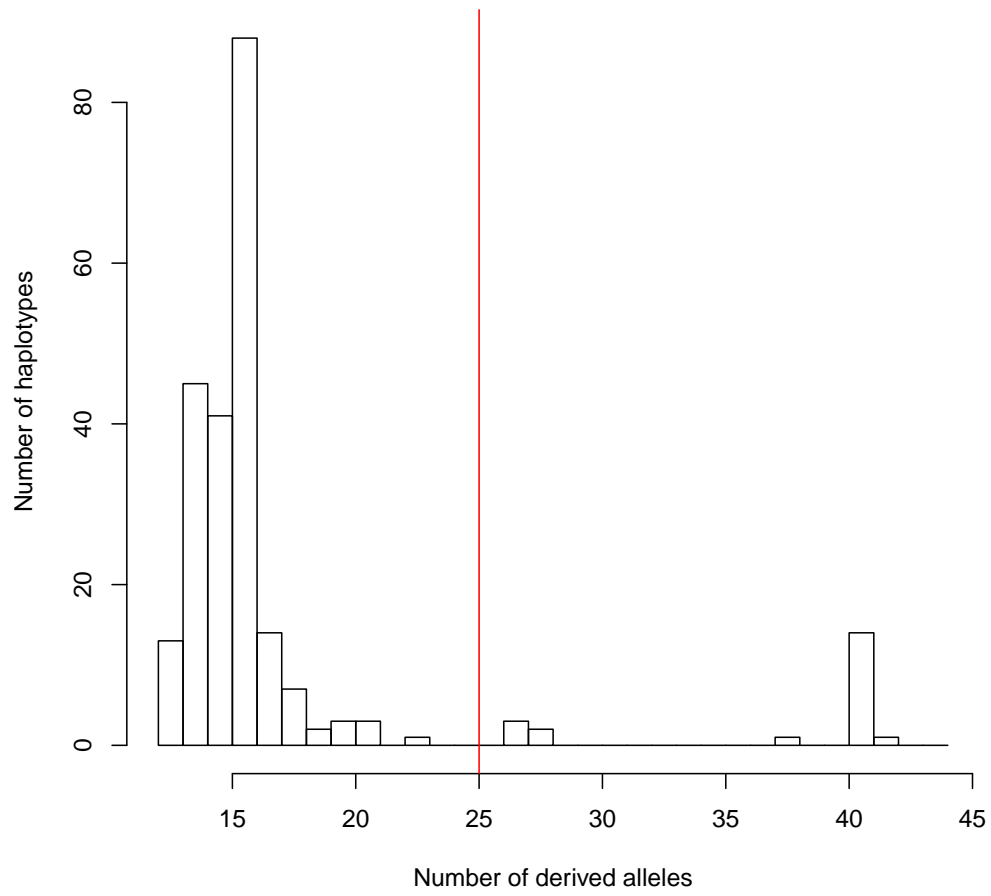

1\_202403000

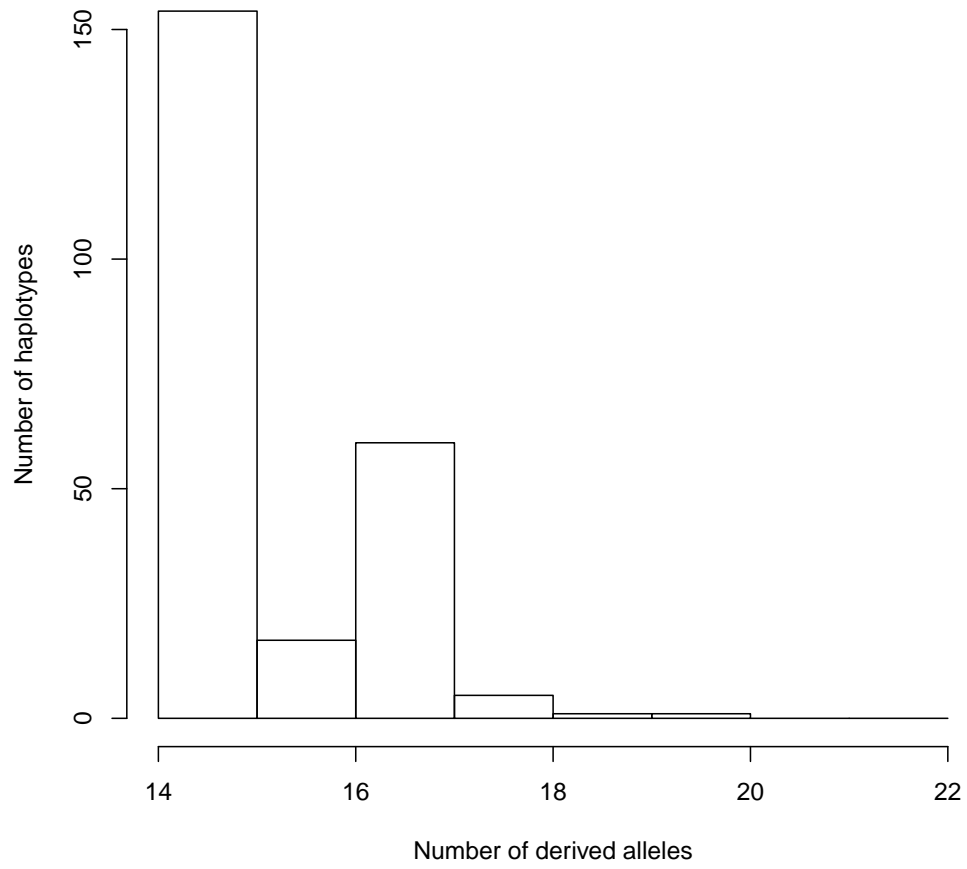

21\_24150000

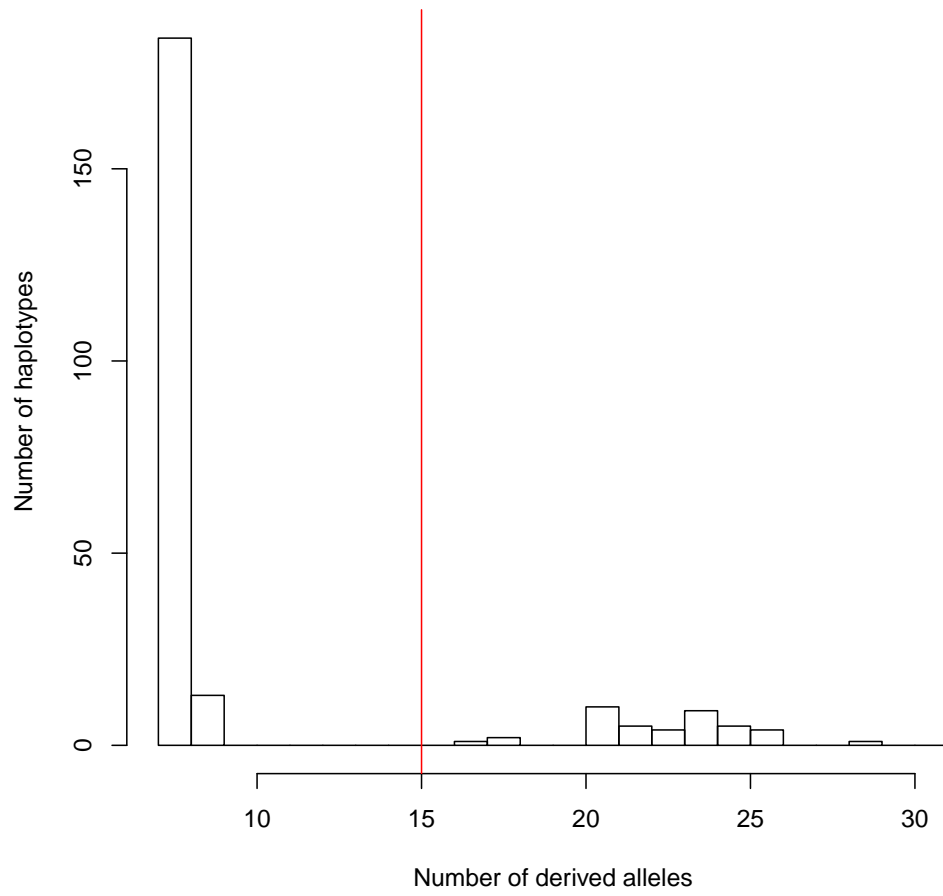

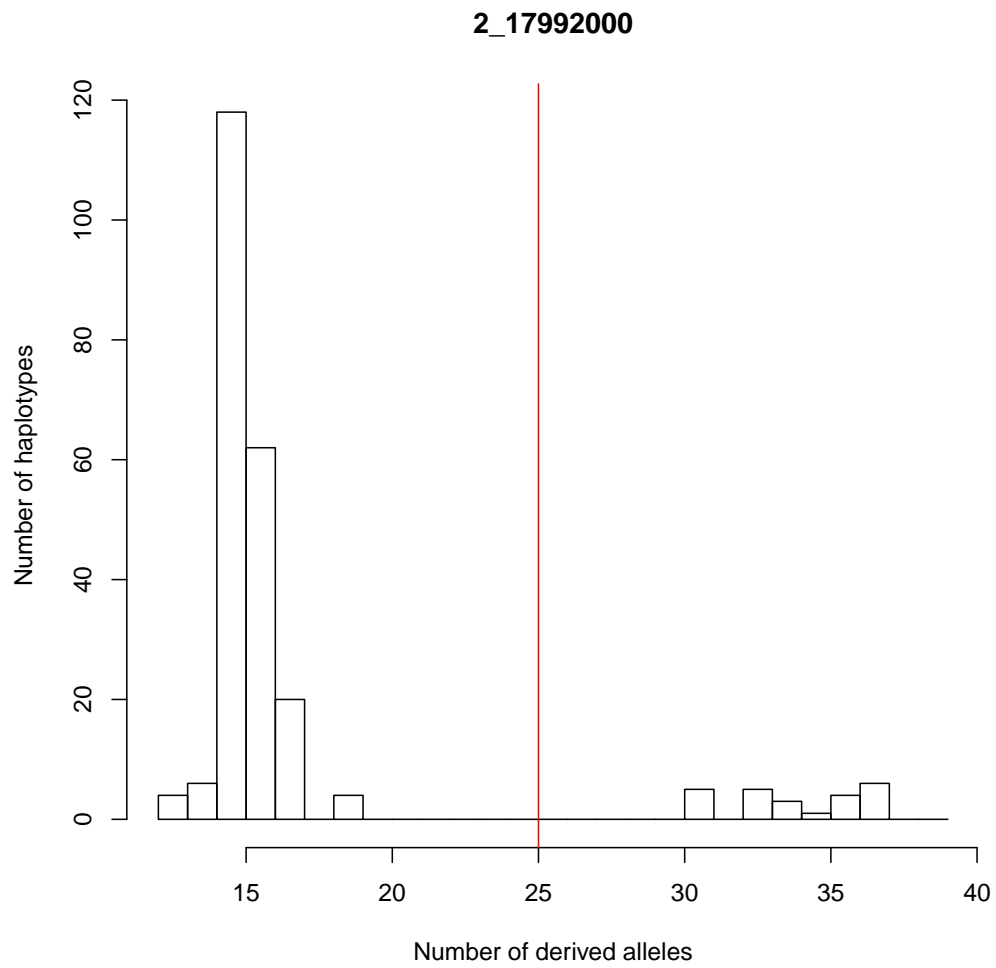

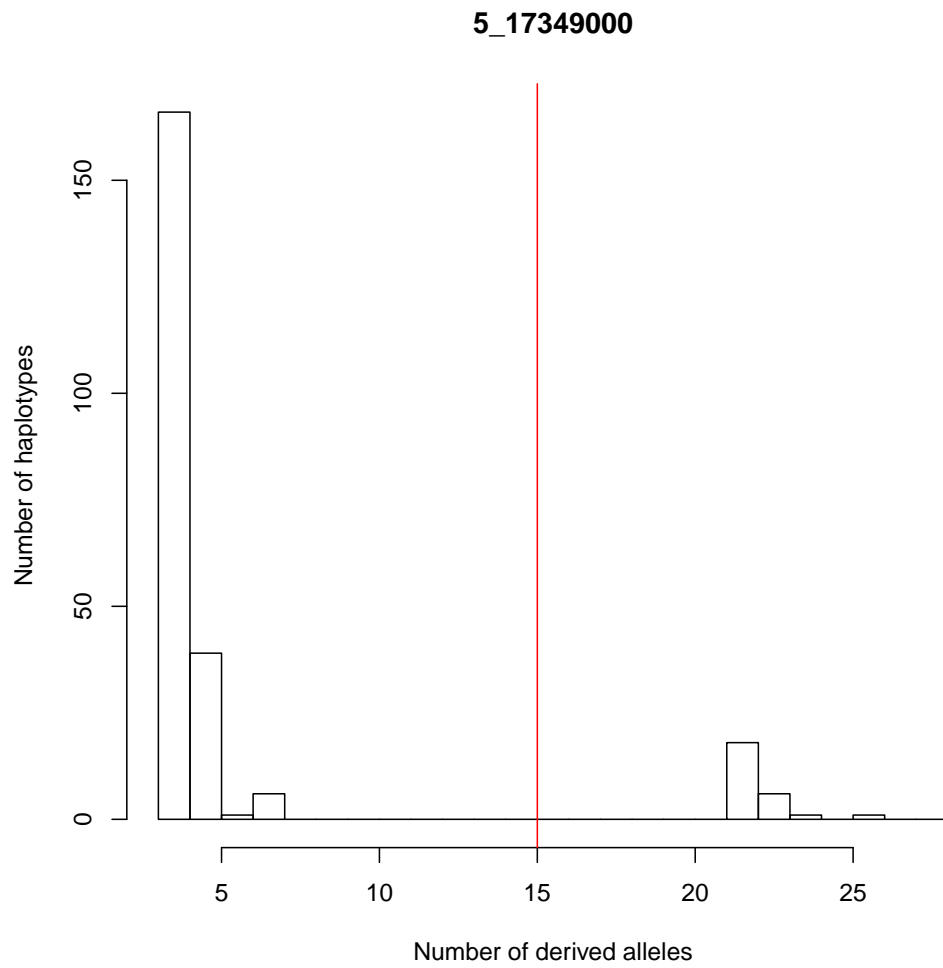

6\_114211000

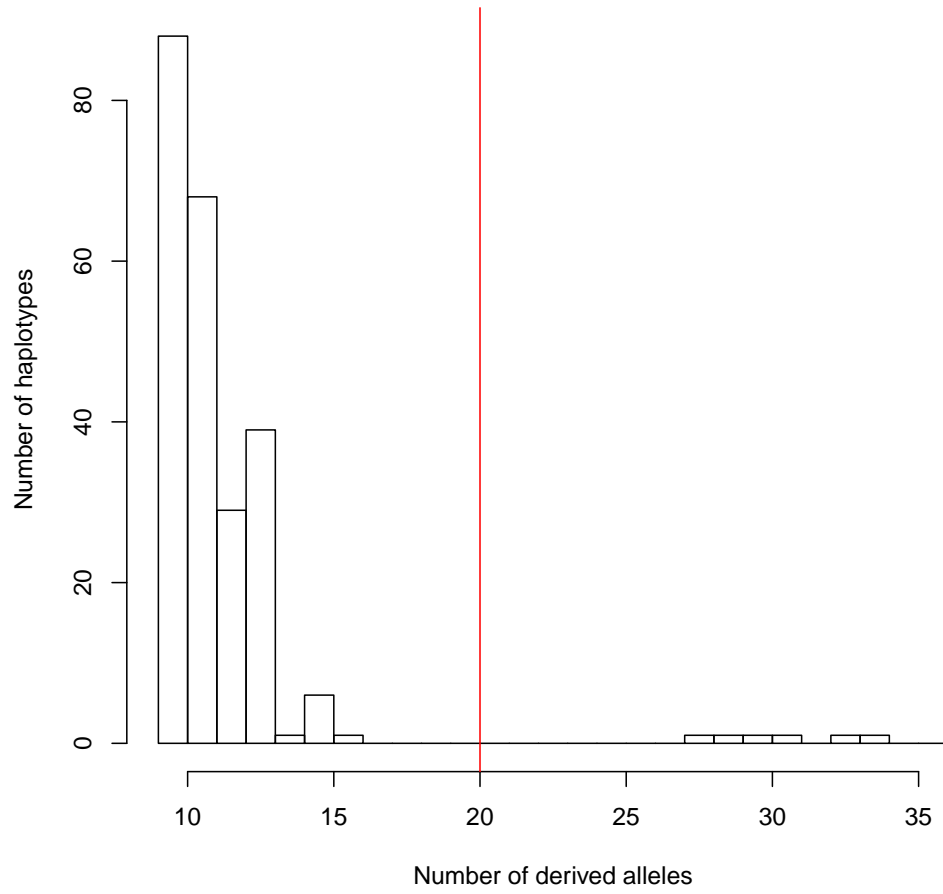

6\_130907000

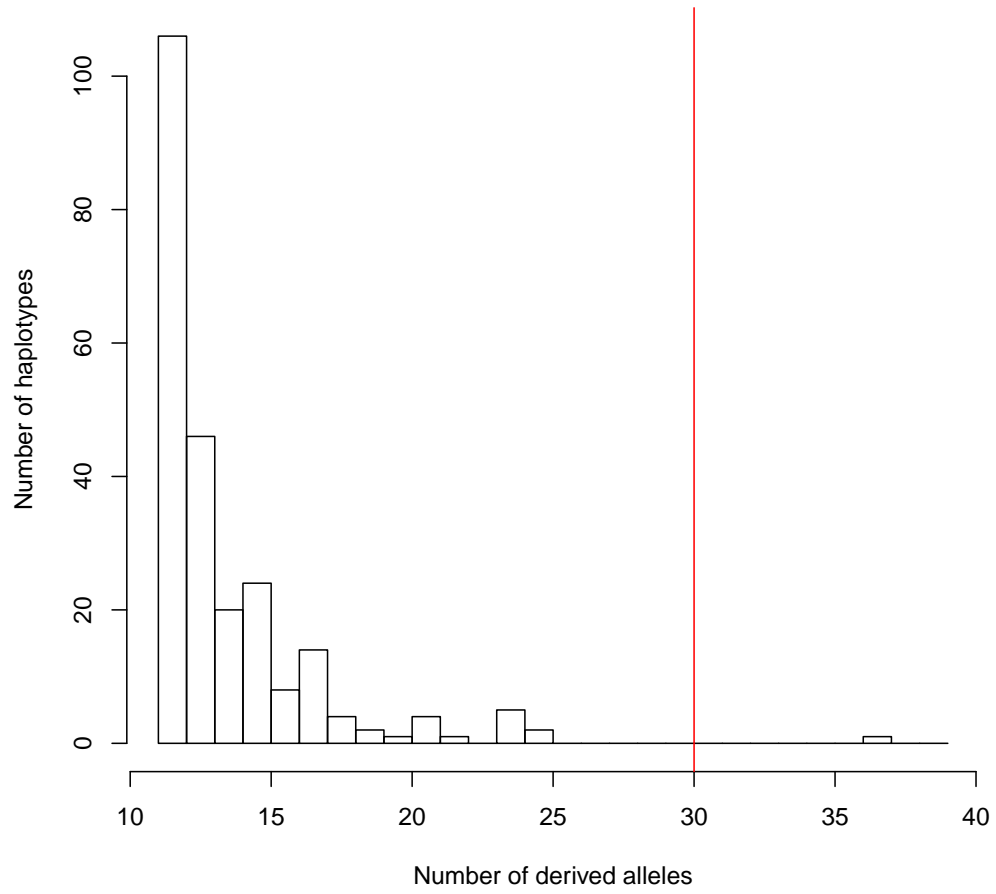

8\_140118000

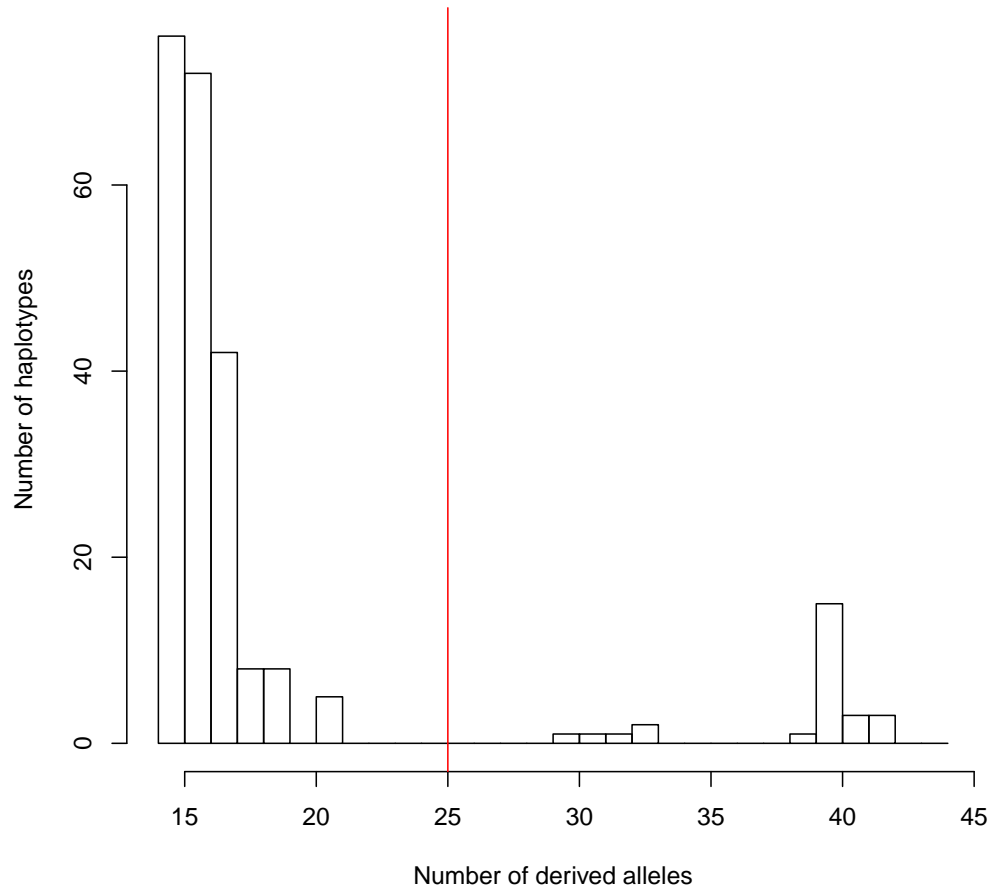

8\_41985000

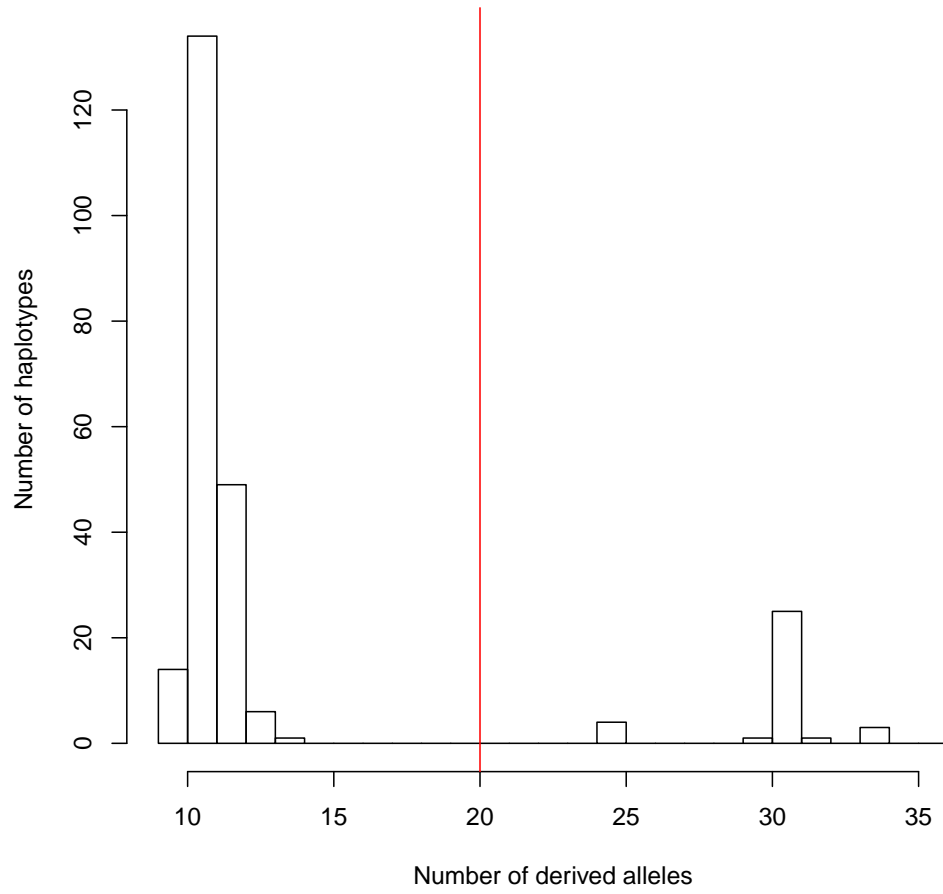

Supplement: S8 Fig — (PDF) [file pgen.1006549.s008.pdf]

10\_25031000

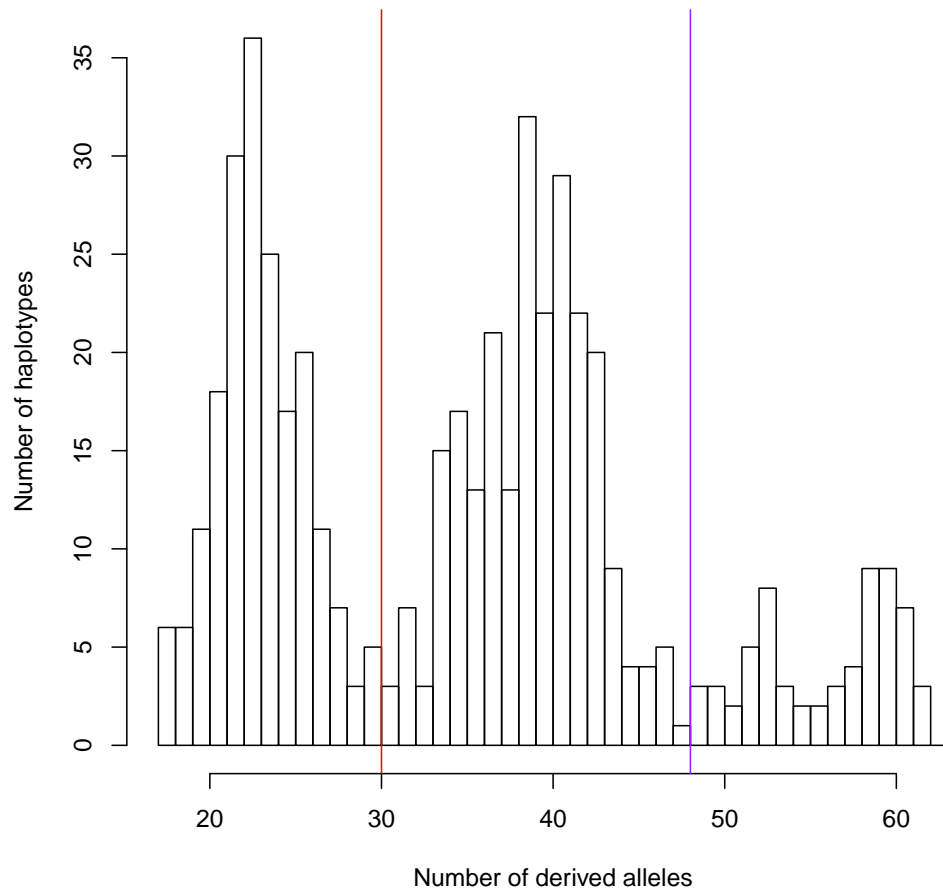

11\_1399000

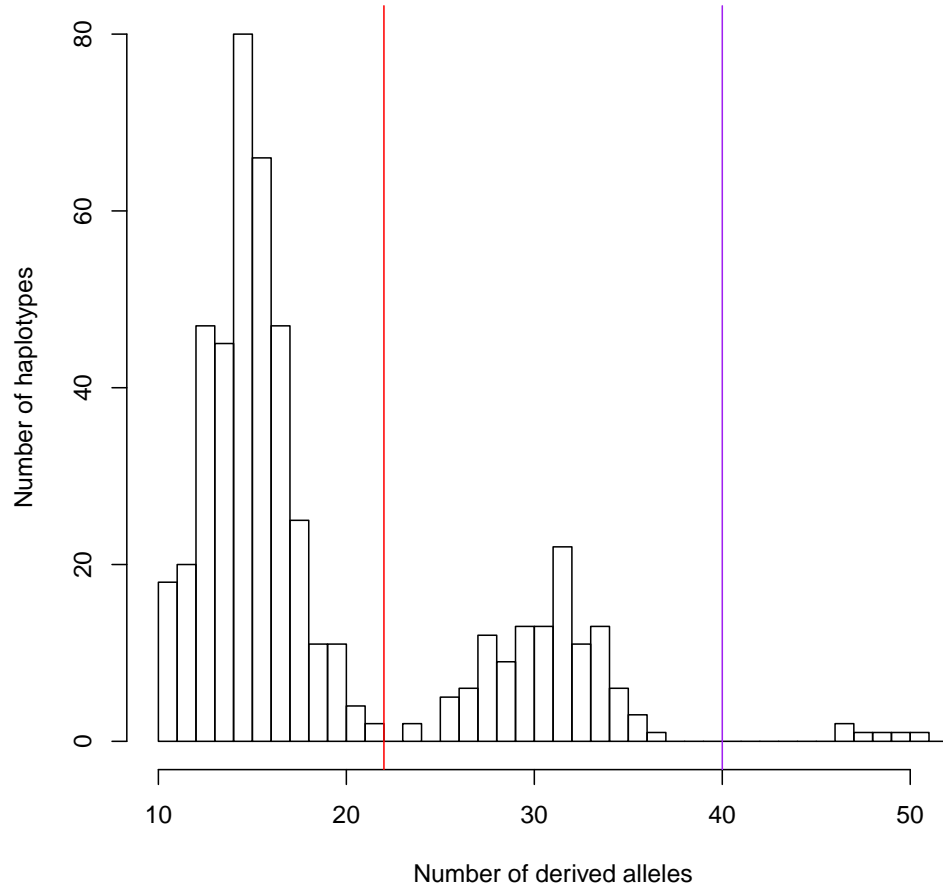

12\_44619000

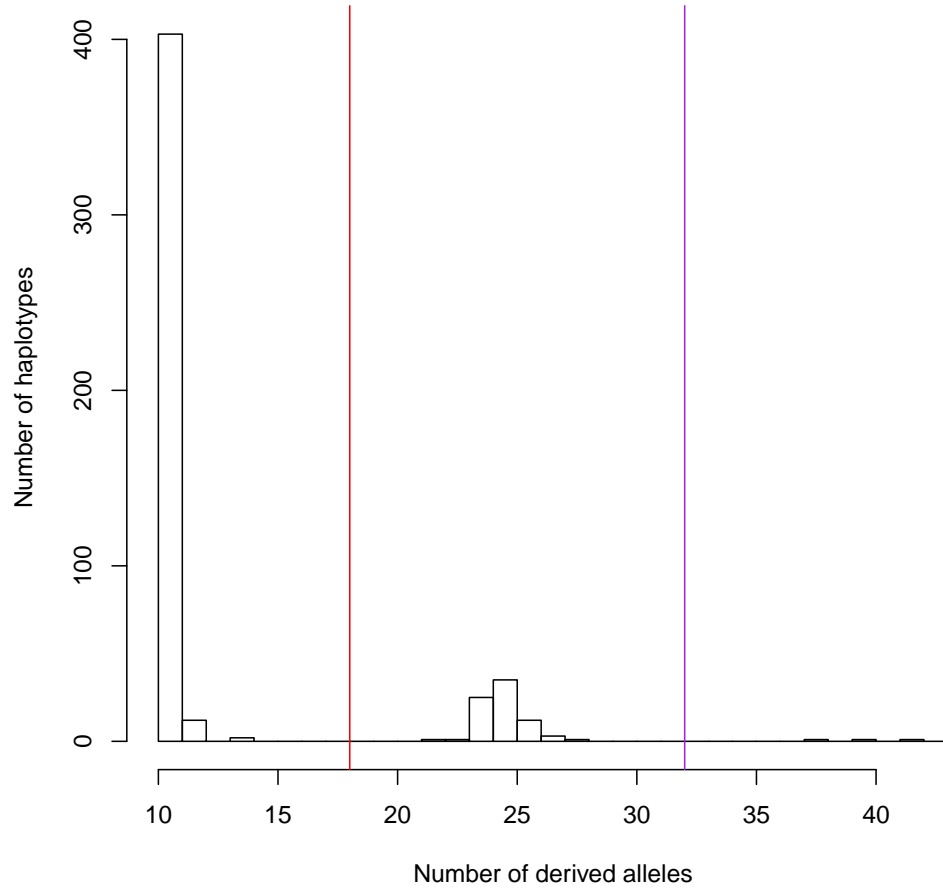

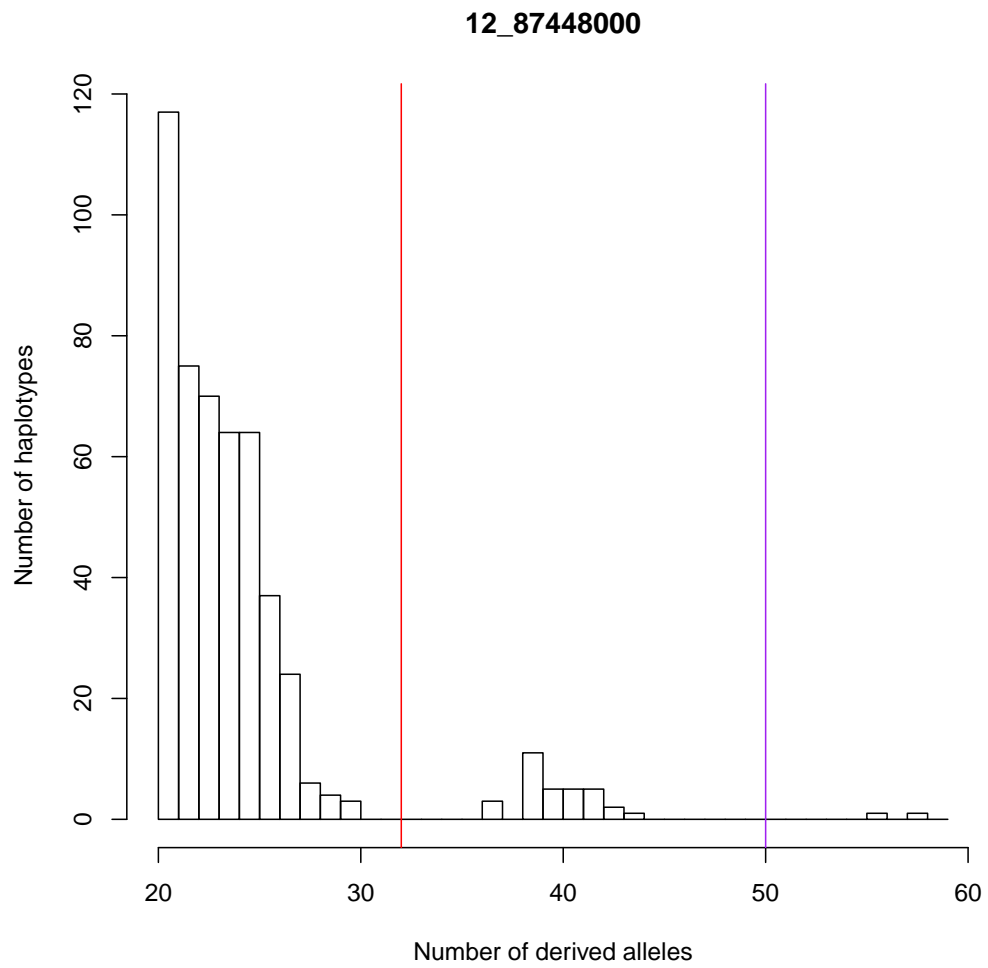

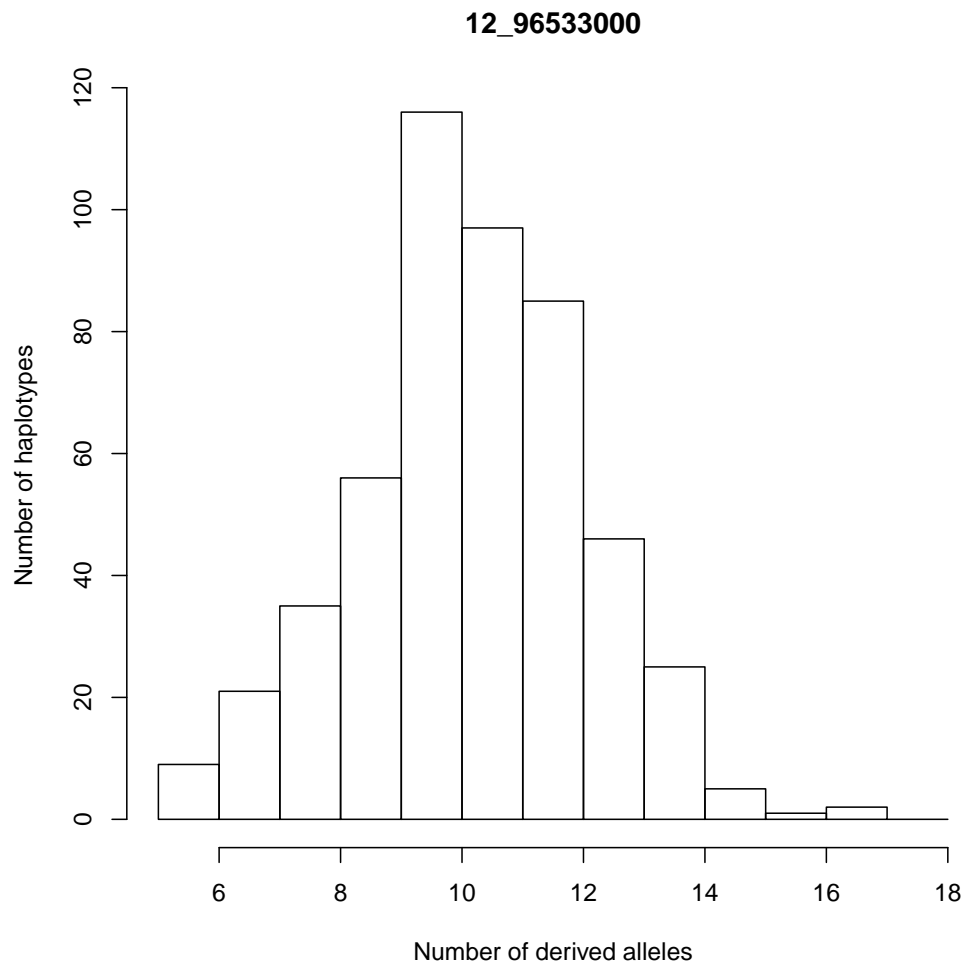

12\_99859000

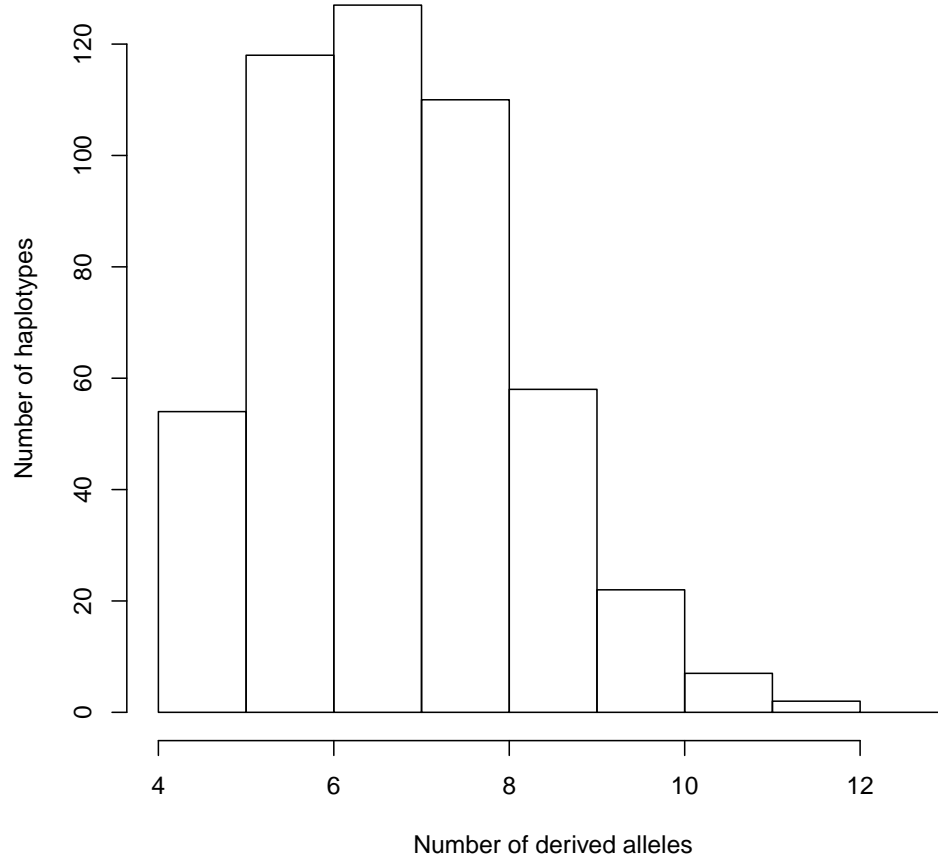

14\_90442000

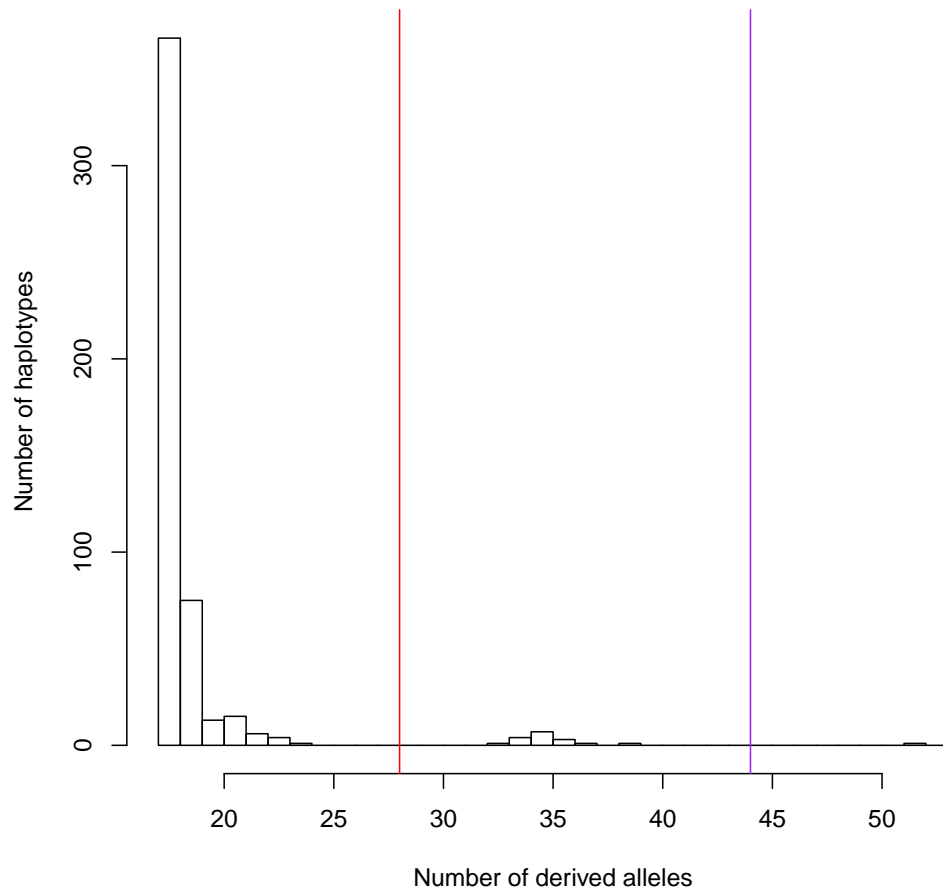

16\_32378000

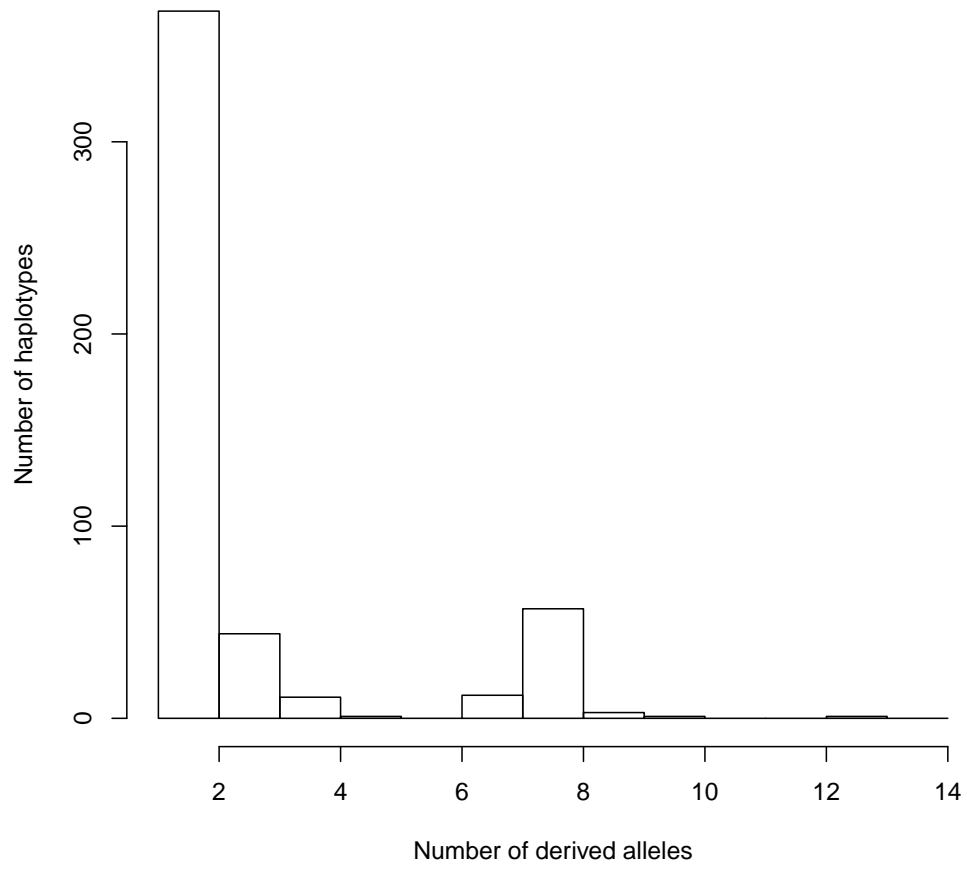

**17\_15866000**

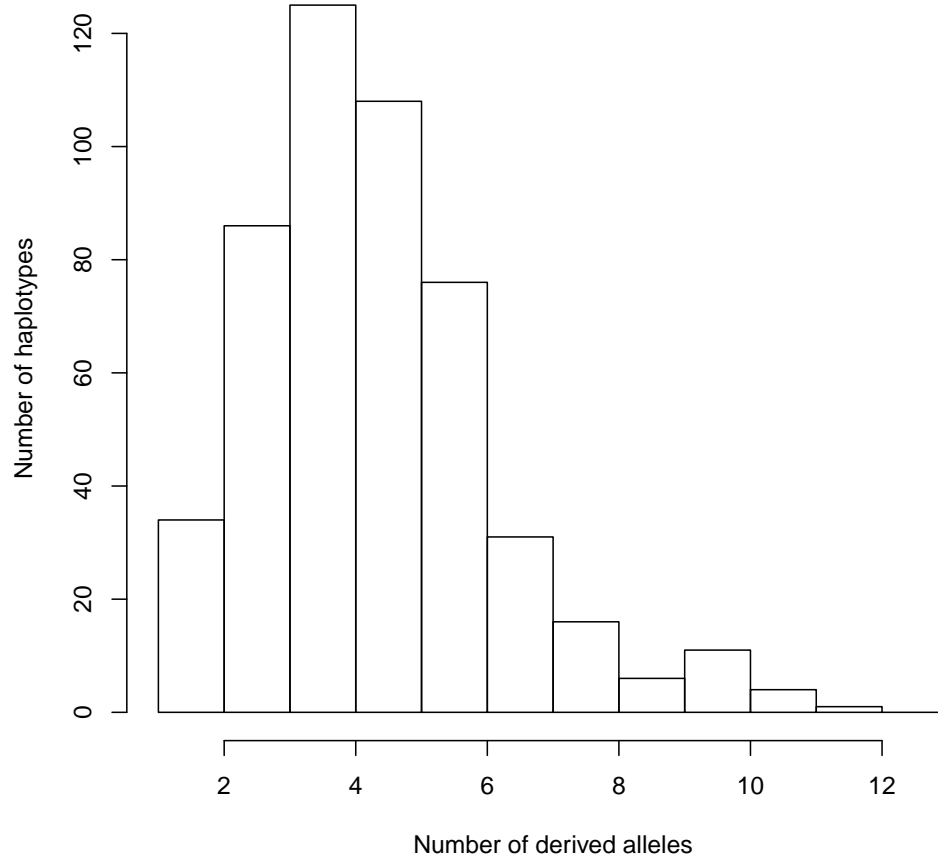

**18\_15301000**

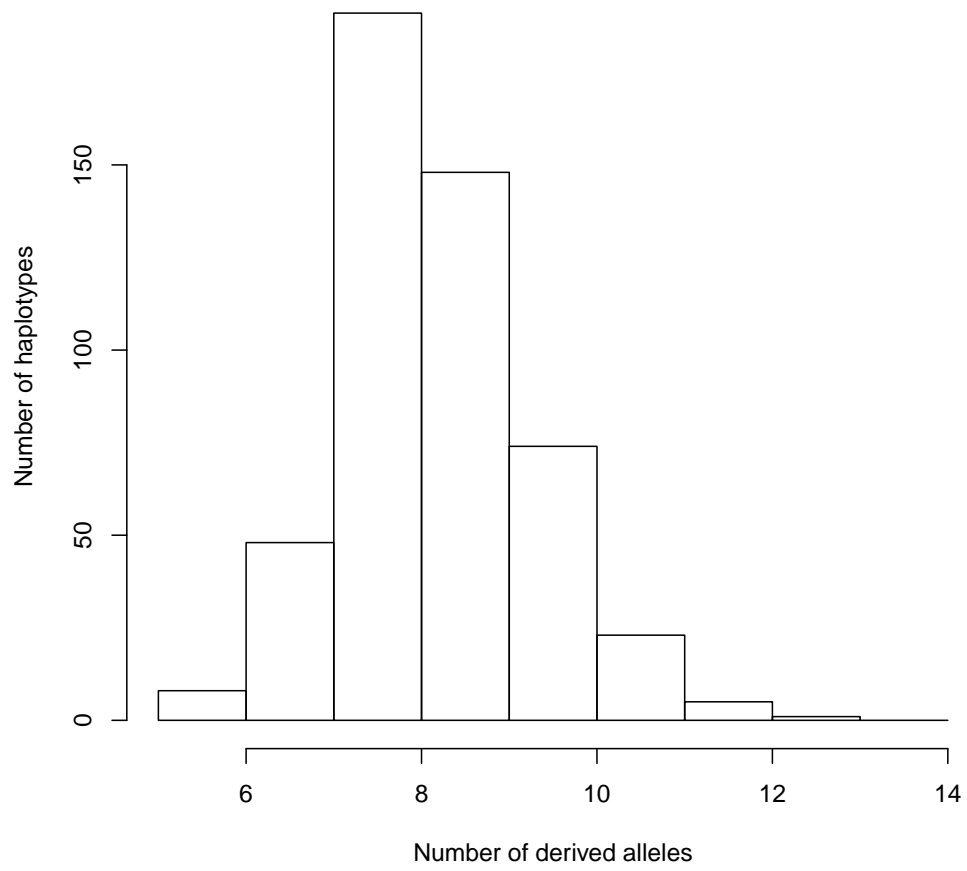

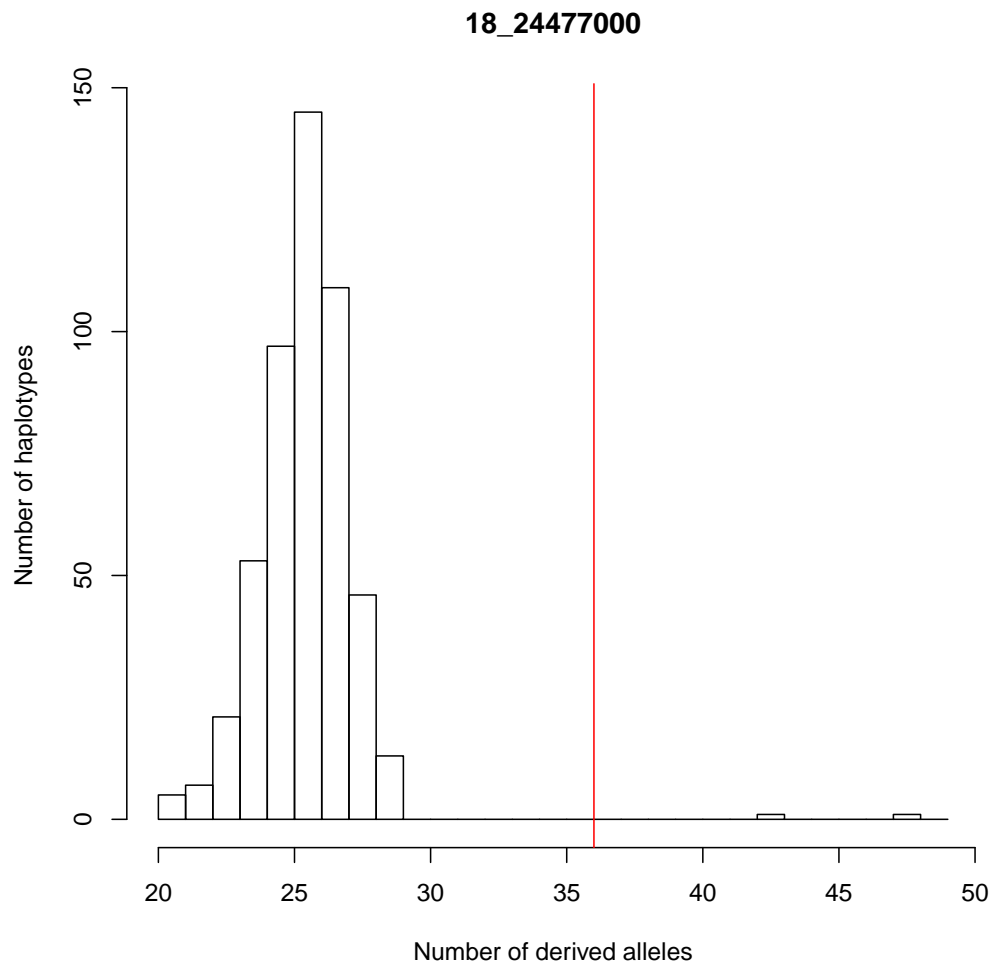

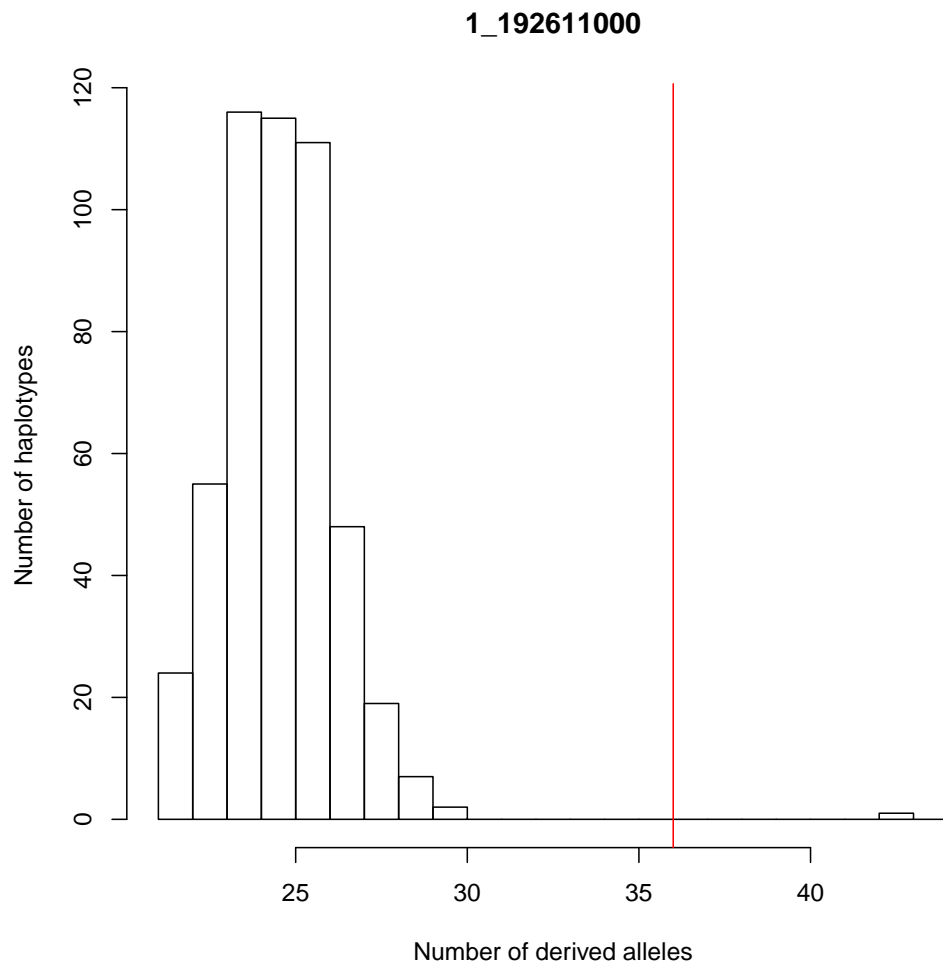

1\_202404000

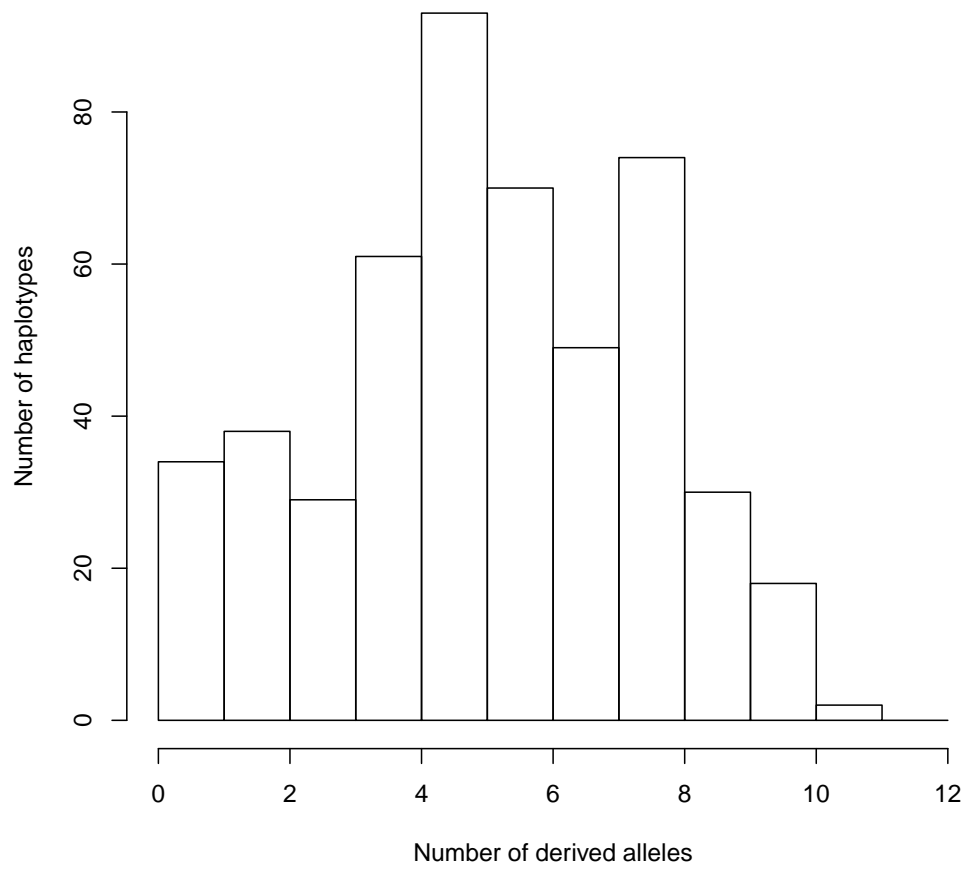

**21\_24151000**

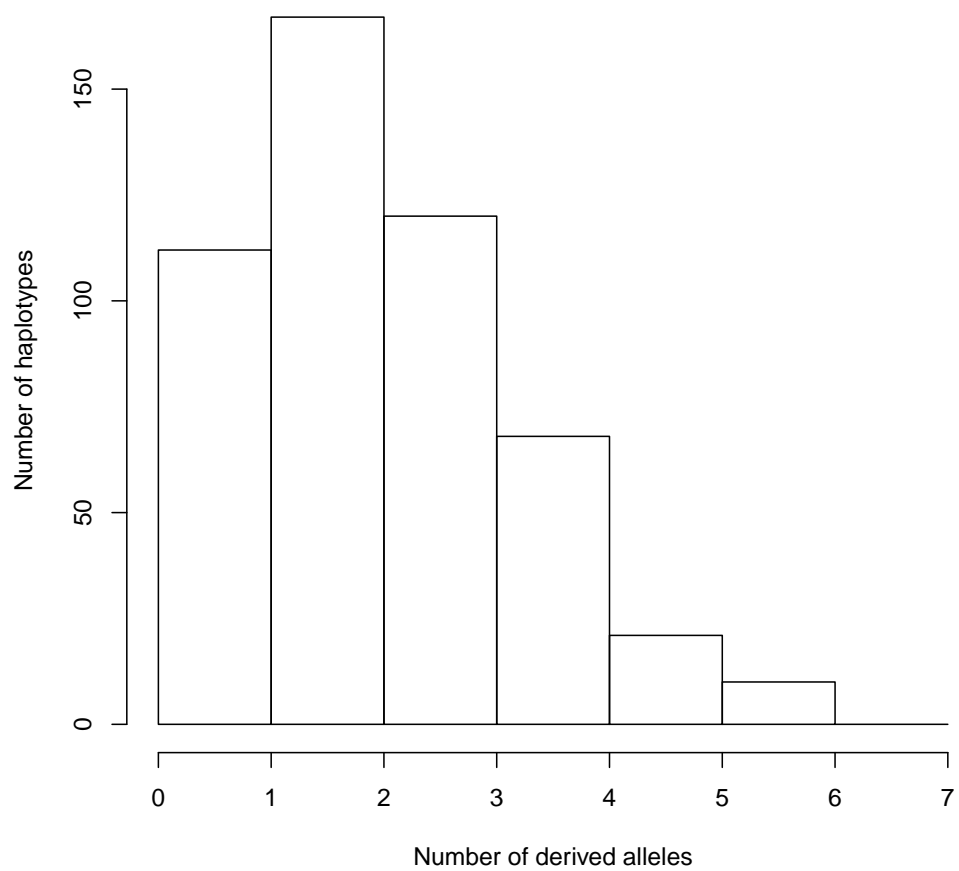

**2\_17992000**

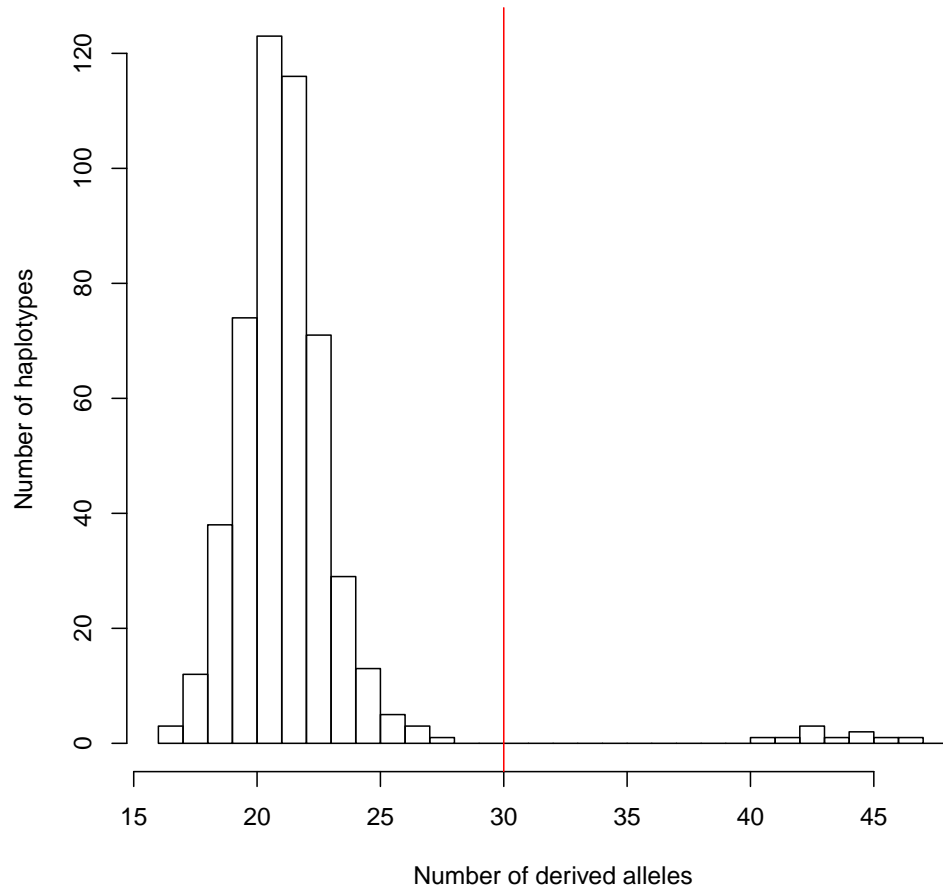

5\_17349000

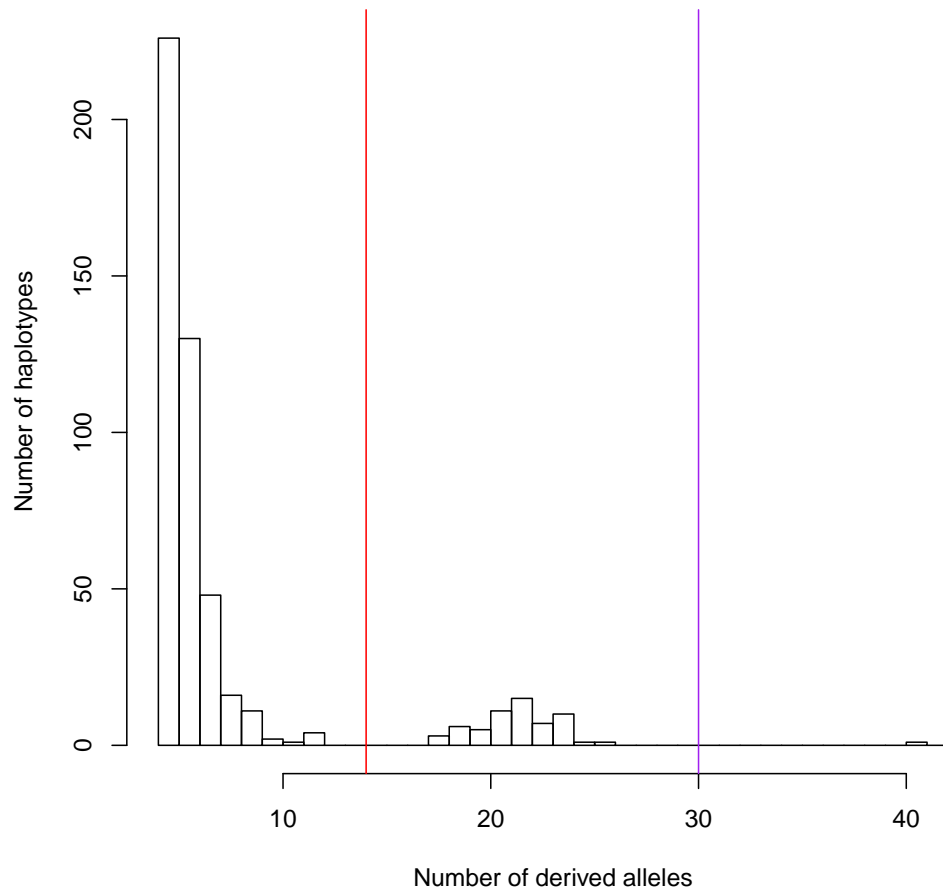

**6\_114211000**

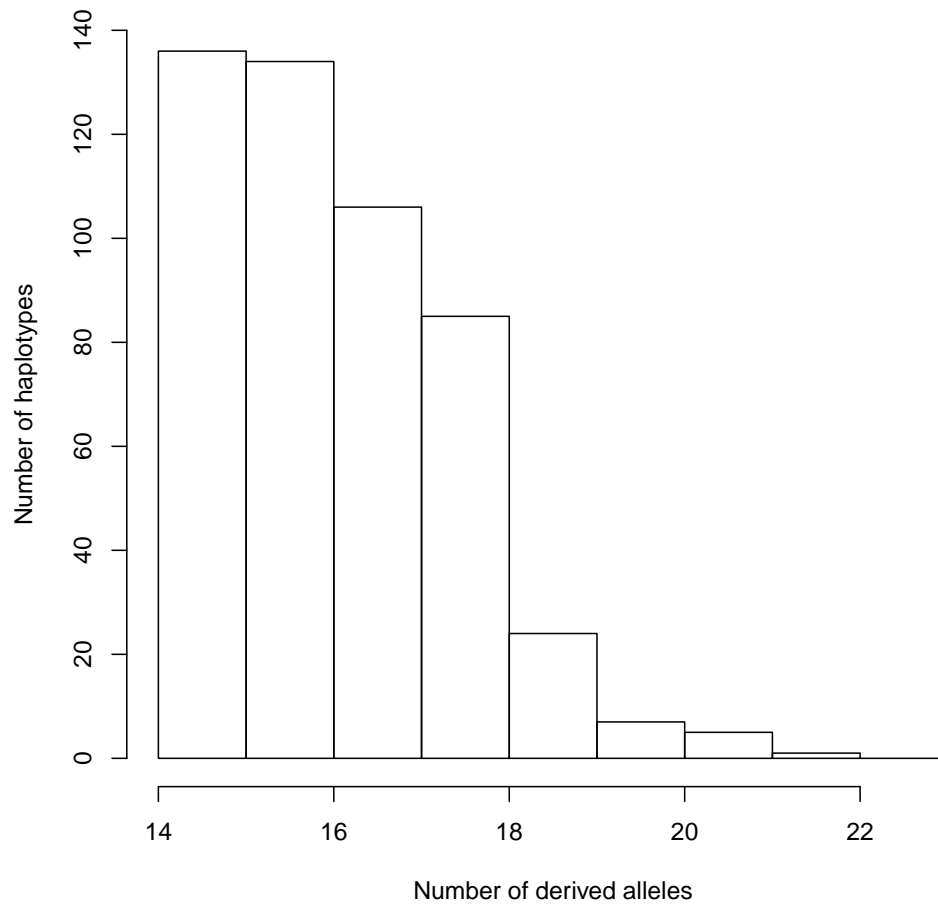

**6\_130907000**

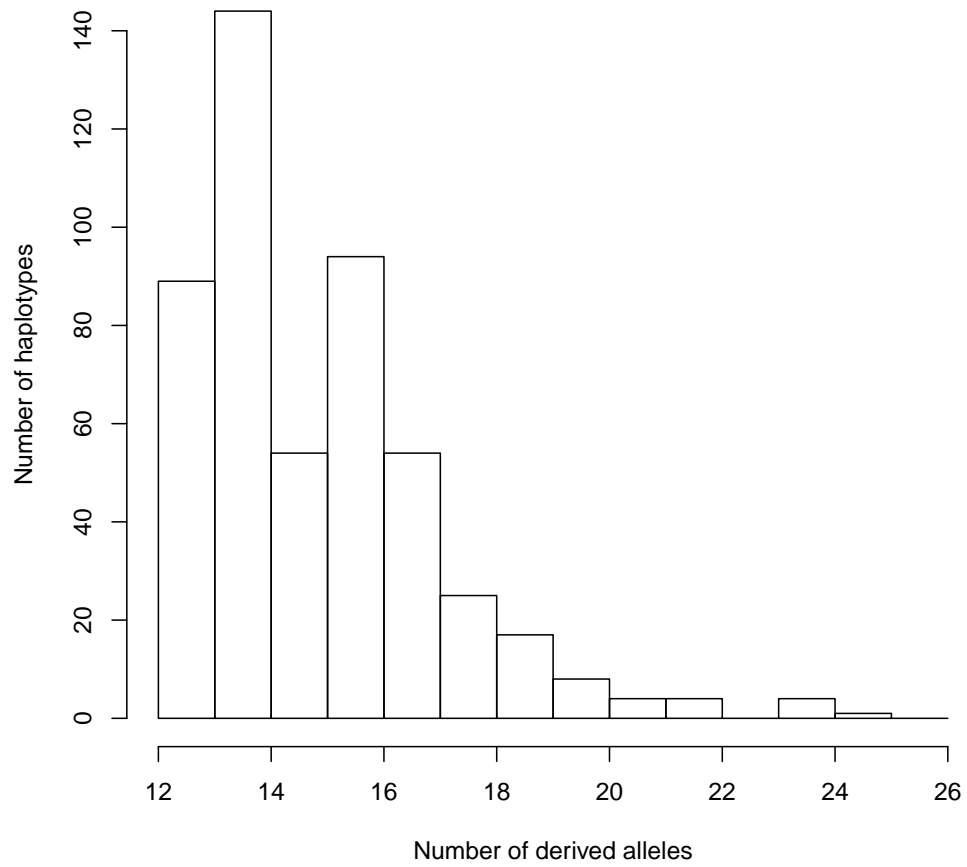

**8\_140118000**

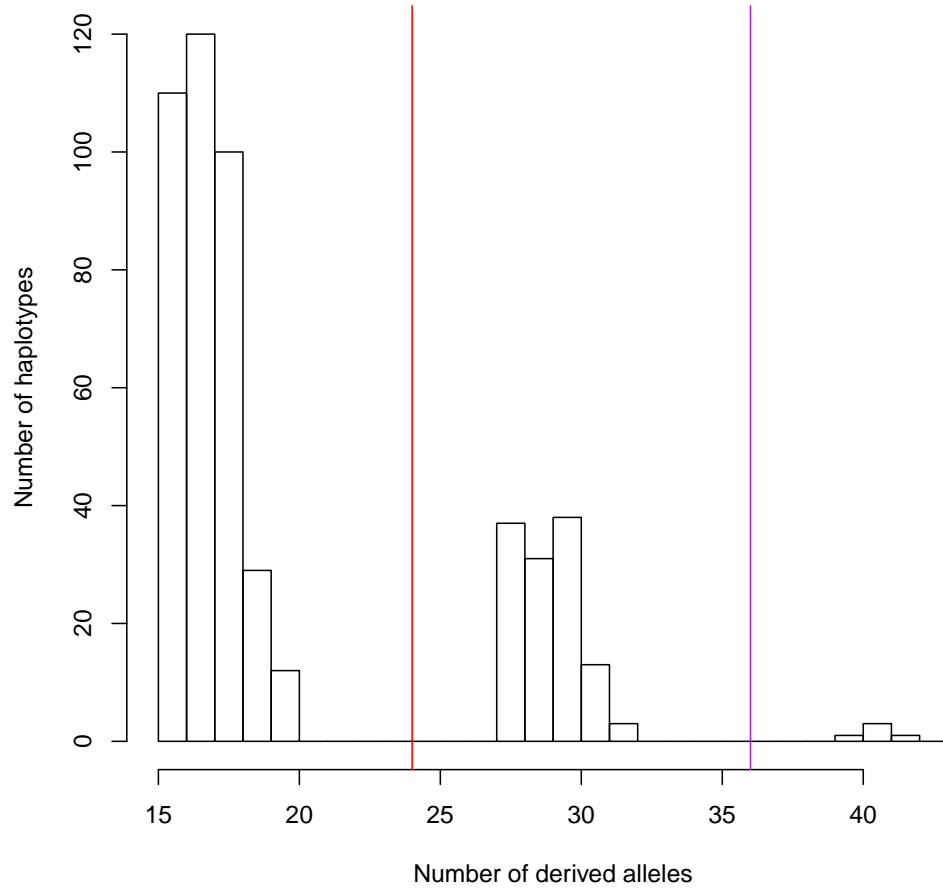

**8\_41985000**

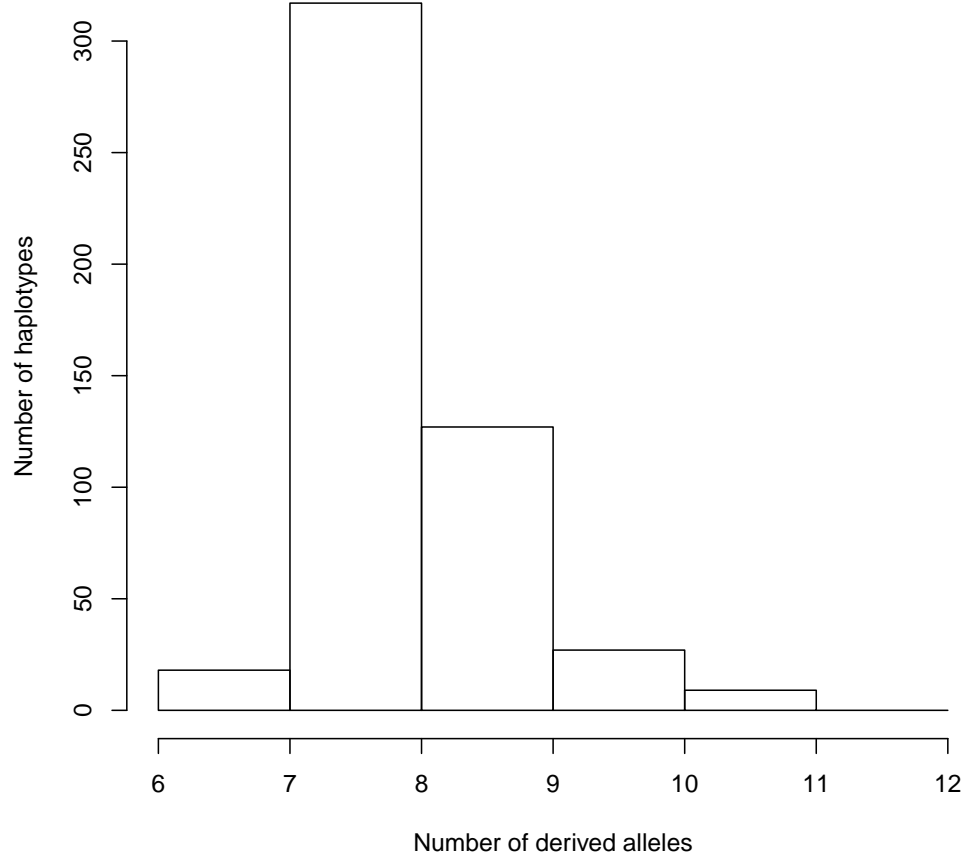

Supplement: S9 Fig — (PDF) [file pgen.1006549.s009.pdf]
