# Supplementary material for: Inference of Candidate Germline Mutator Loci in Humans from Genome-Wide Haplotype Data
Source: PLoS Genet. 2017 Jan 17;13(1):e1006549. doi: 10.1371/journal.pgen.1006549 (PMC5283766; doi:10.1371/journal.pgen.1006549)

Locus: Chr1, 202Mb

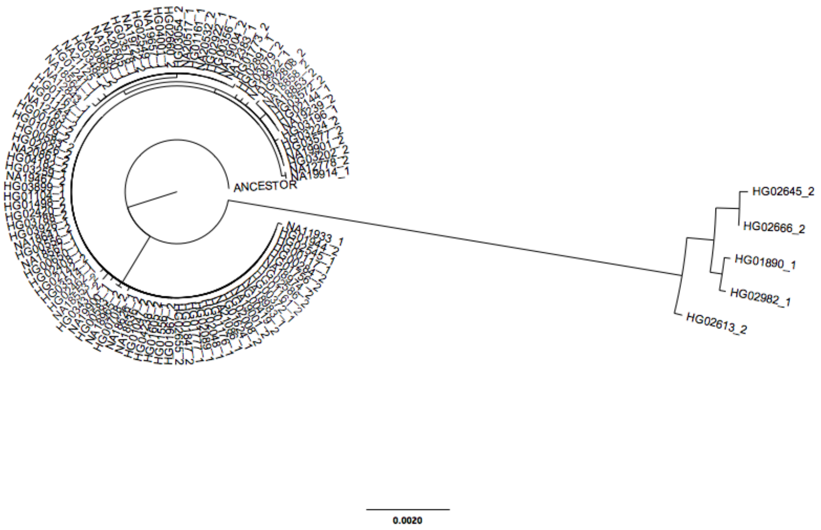

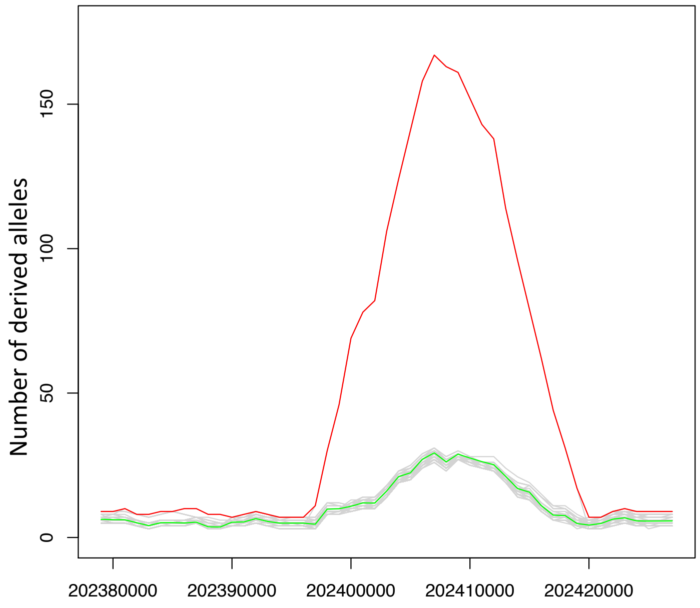

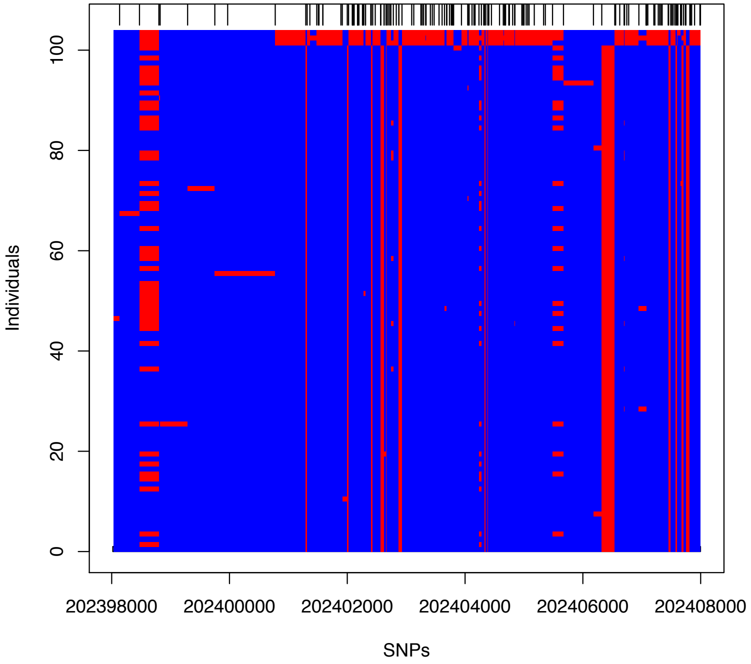

Locus: Chr18, 15Mb

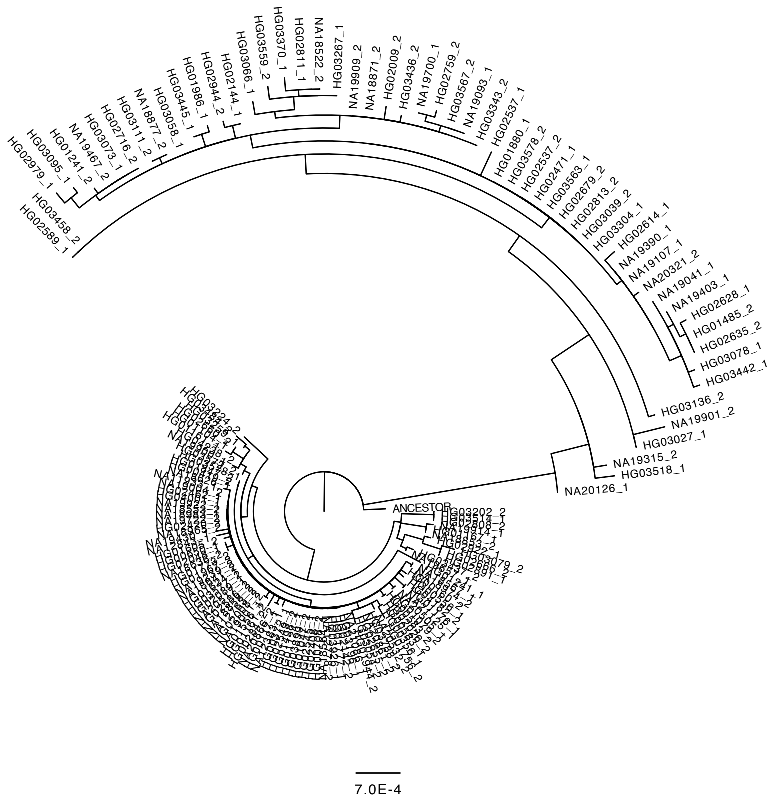

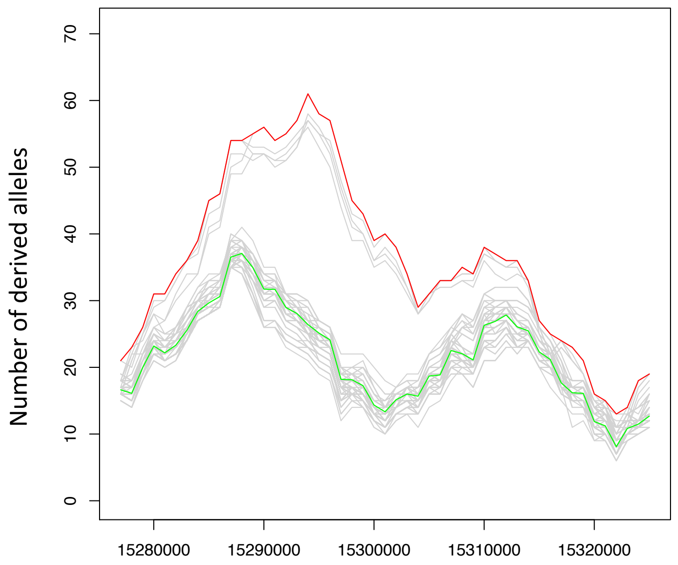

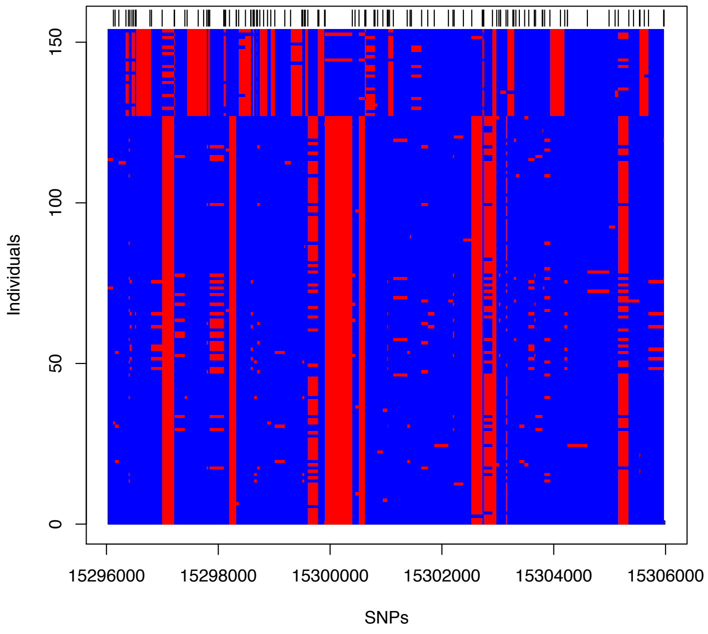

Locus: Chr12, 96.5Mb

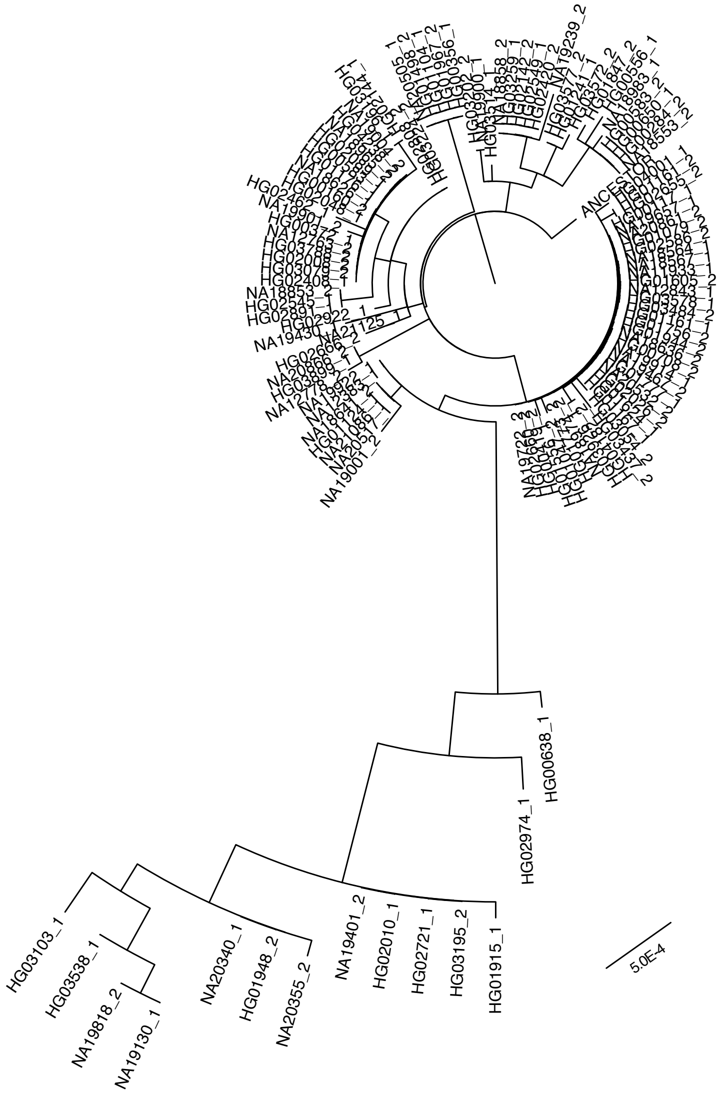

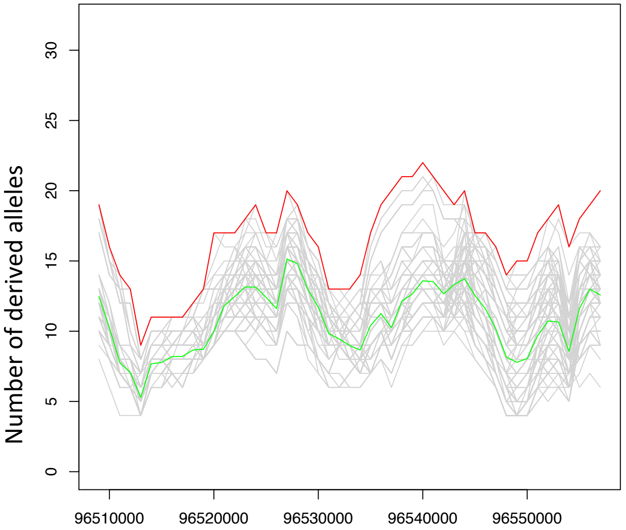

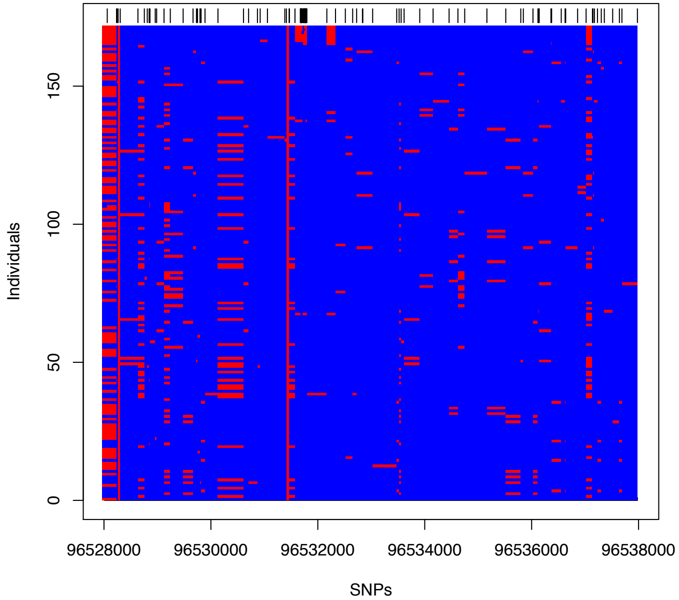

Locus: Chr2, 18Mb

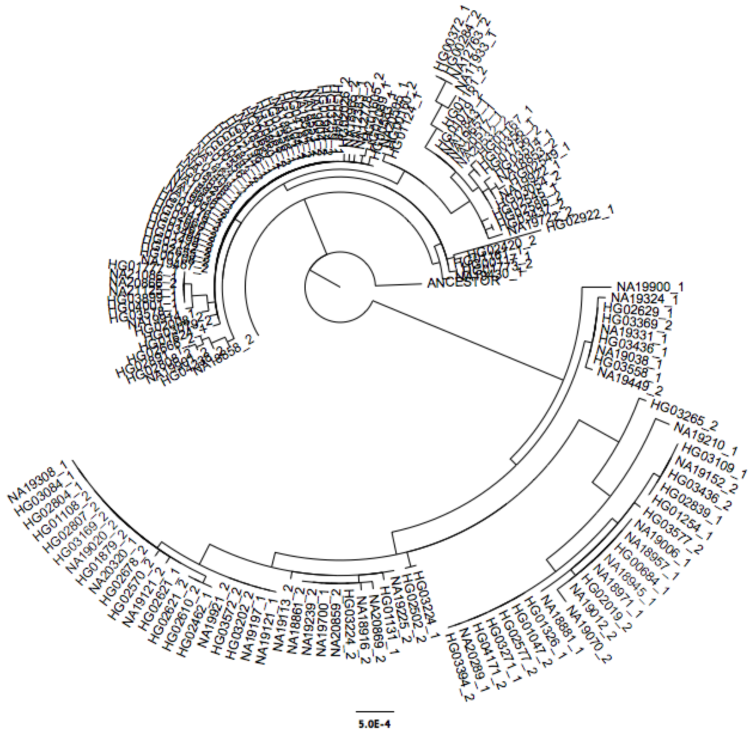

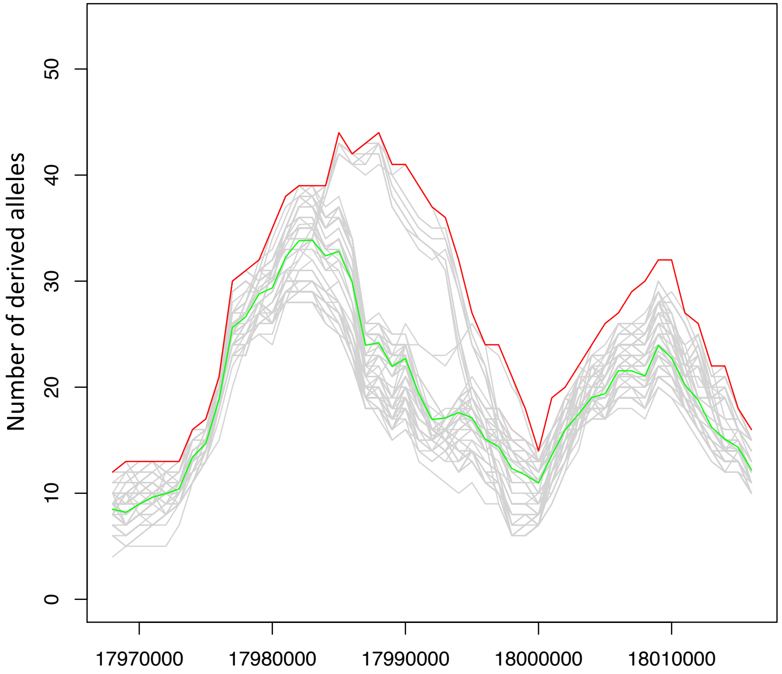

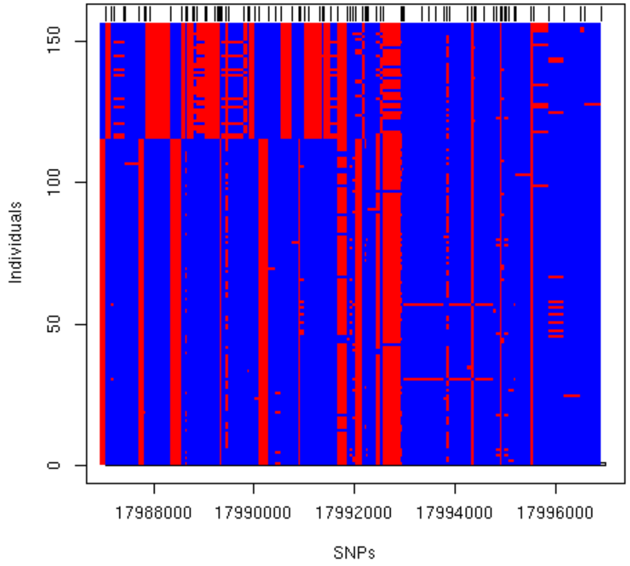

Locus: Chr6, 114Mb

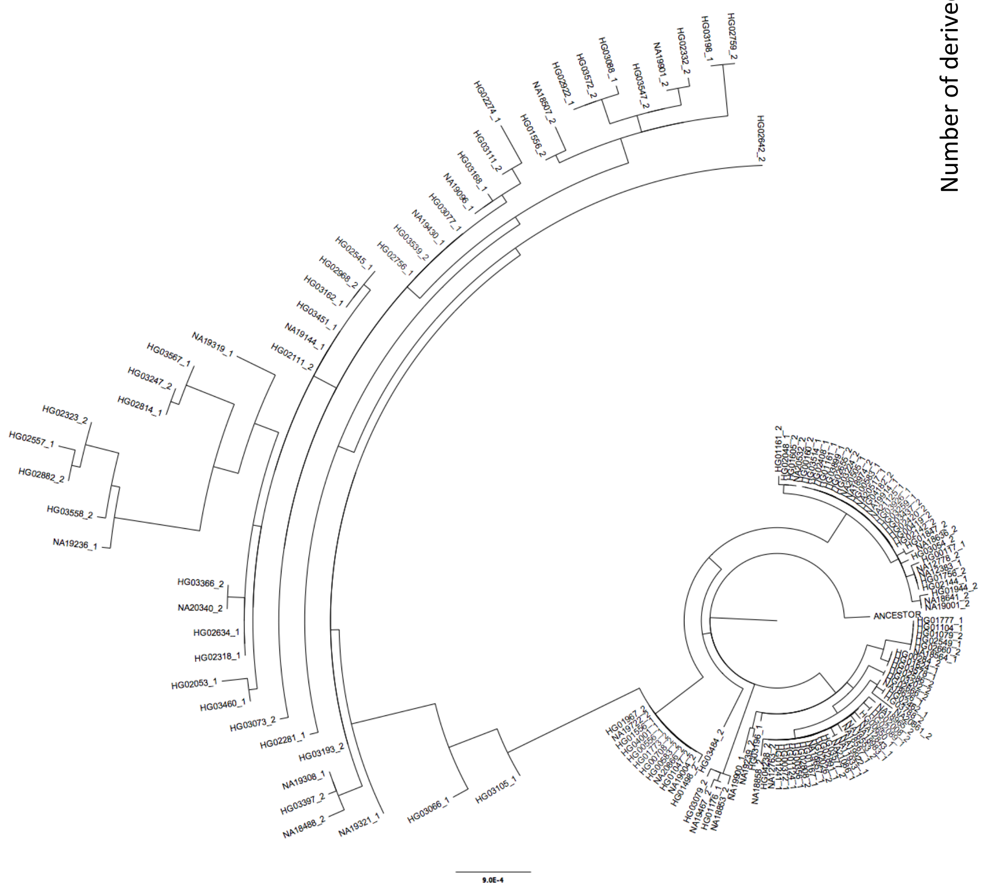

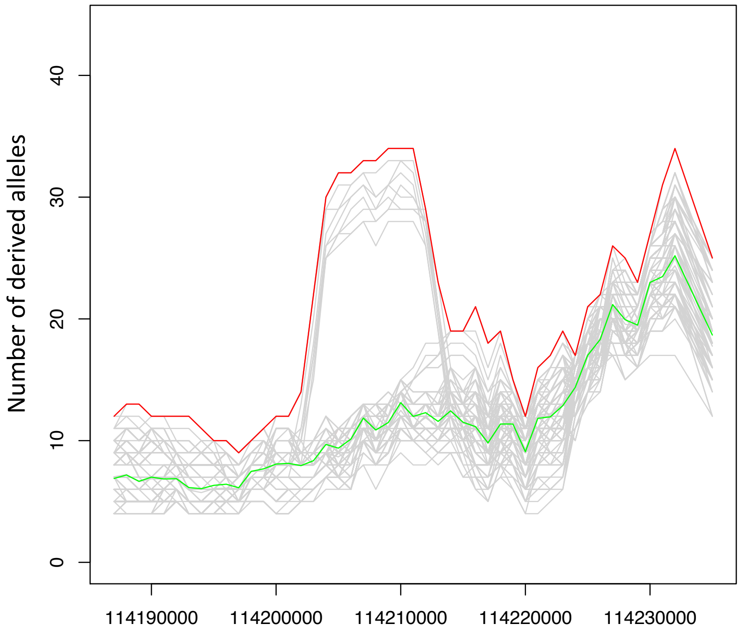

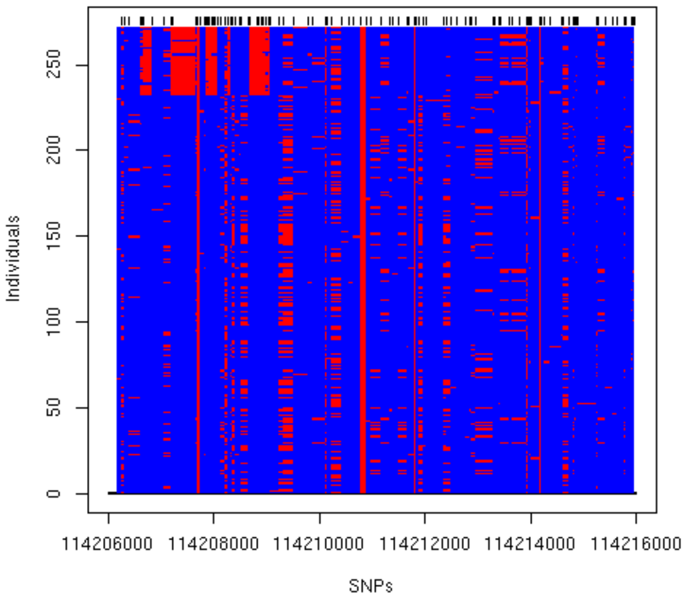

Locus: Chr11, 1.4Mb

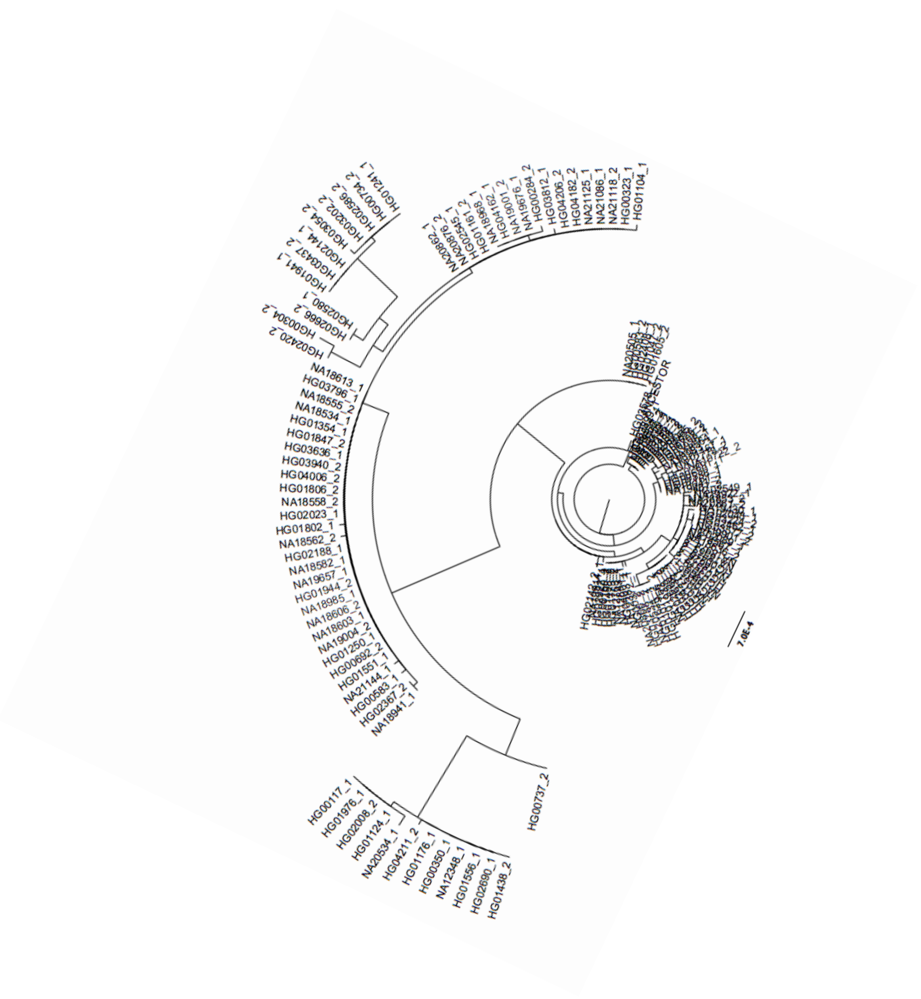

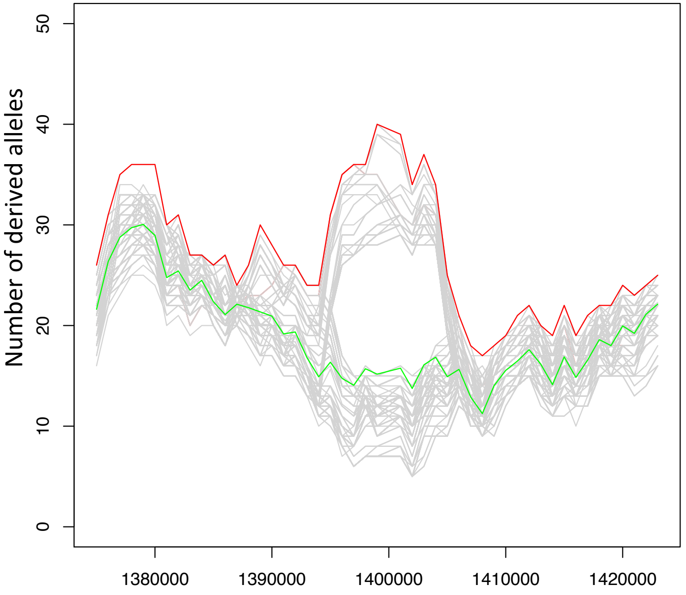

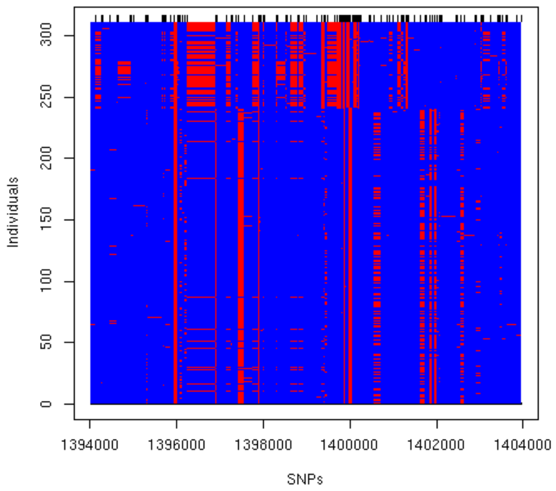

Locus: Chr8, 140Mb

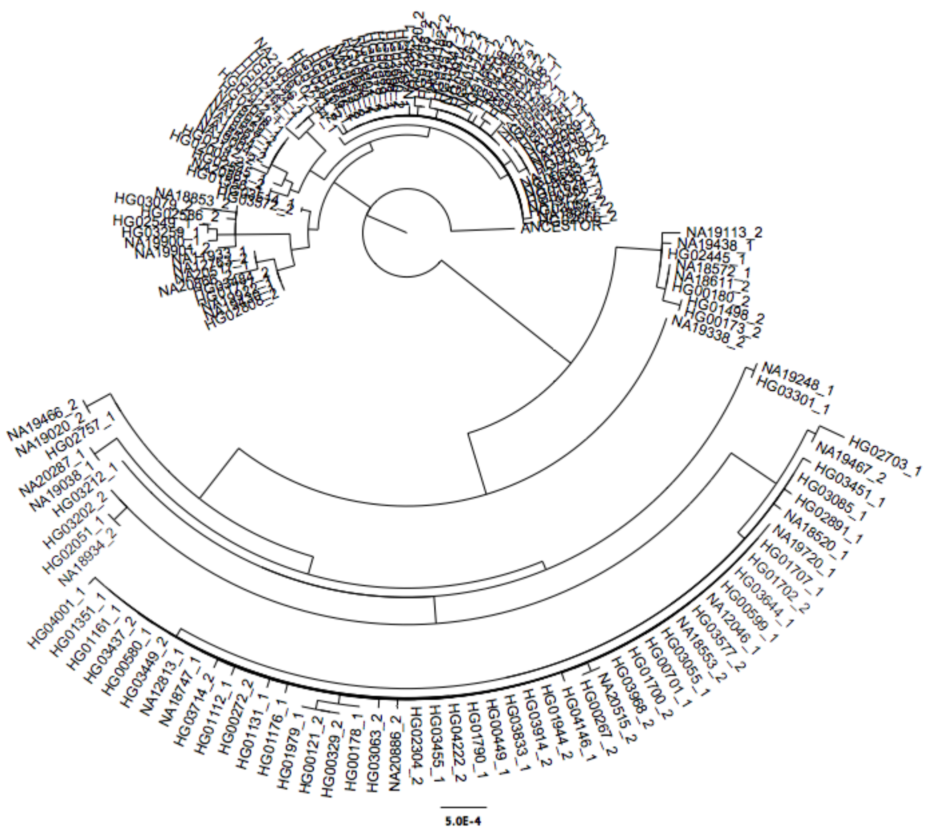

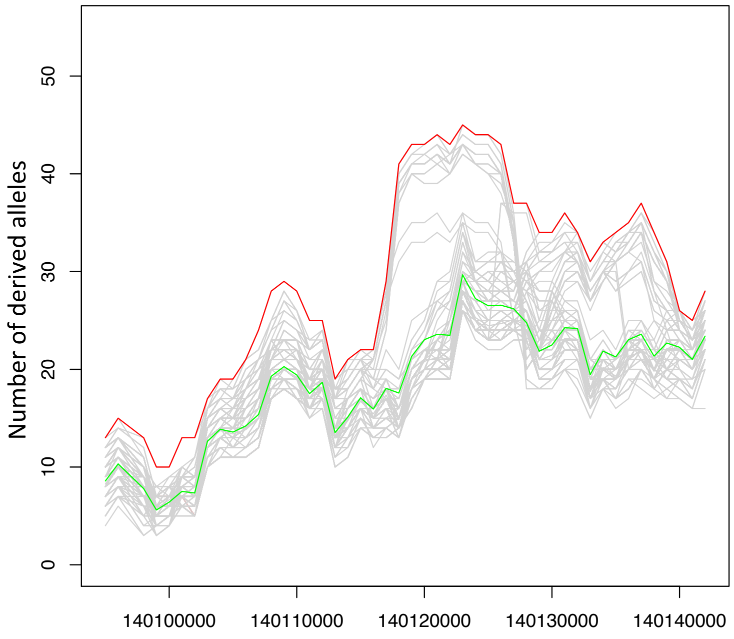

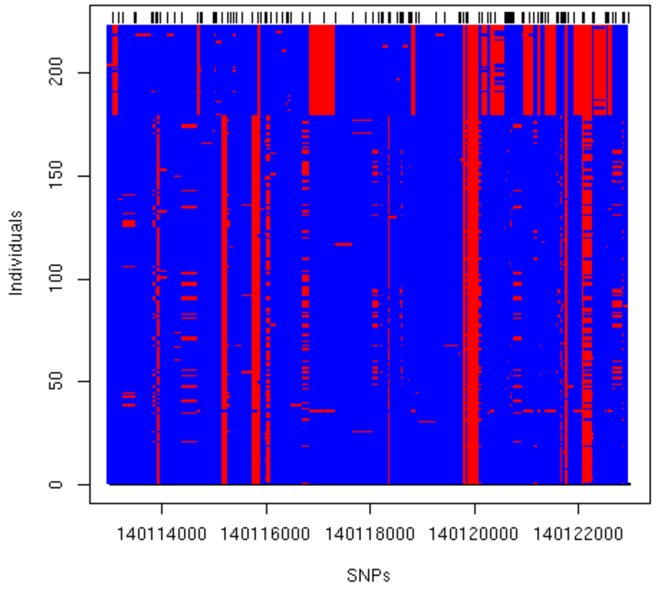

Locus: Chr16, 32Mb

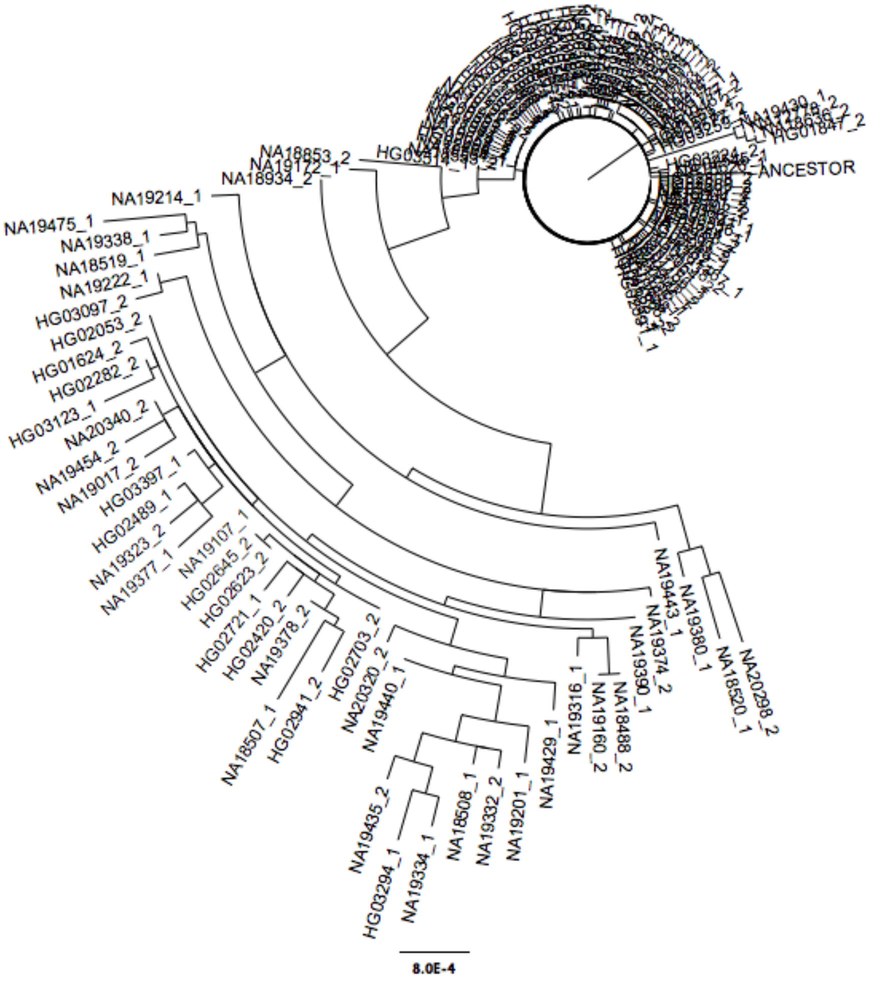

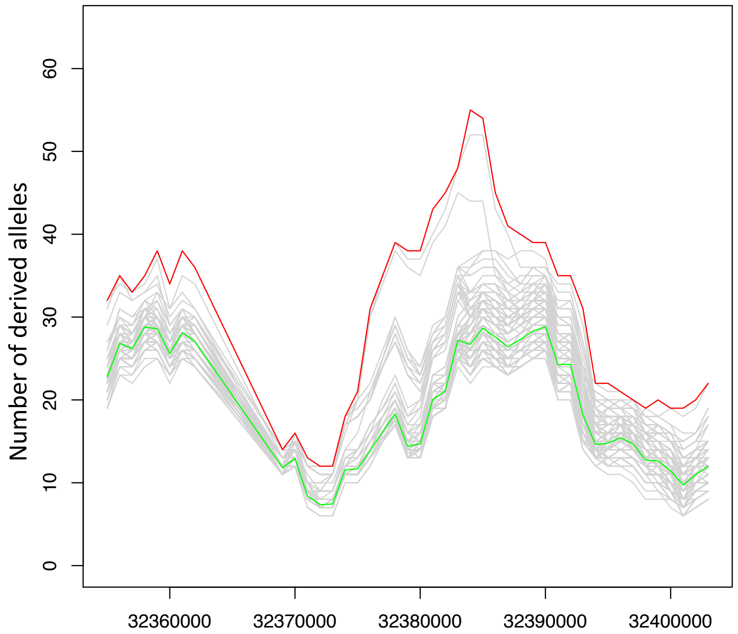

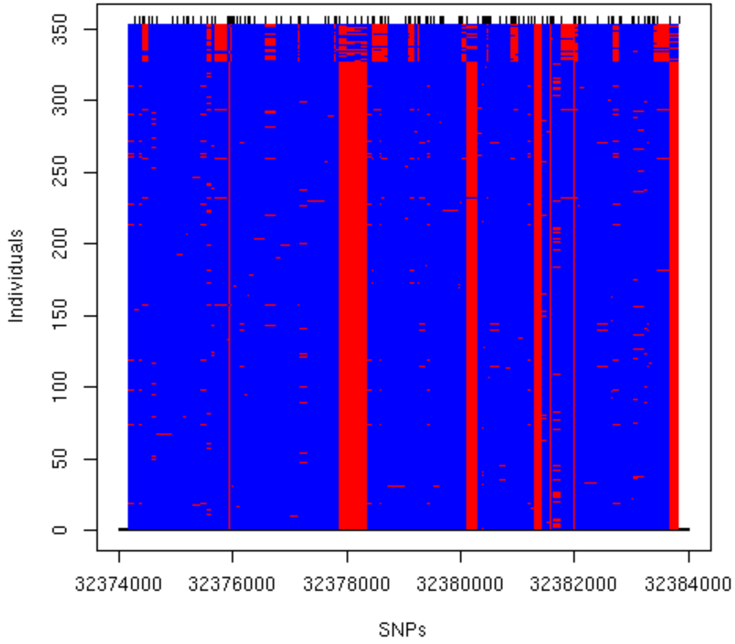

Locus: Chr18, 24Mb

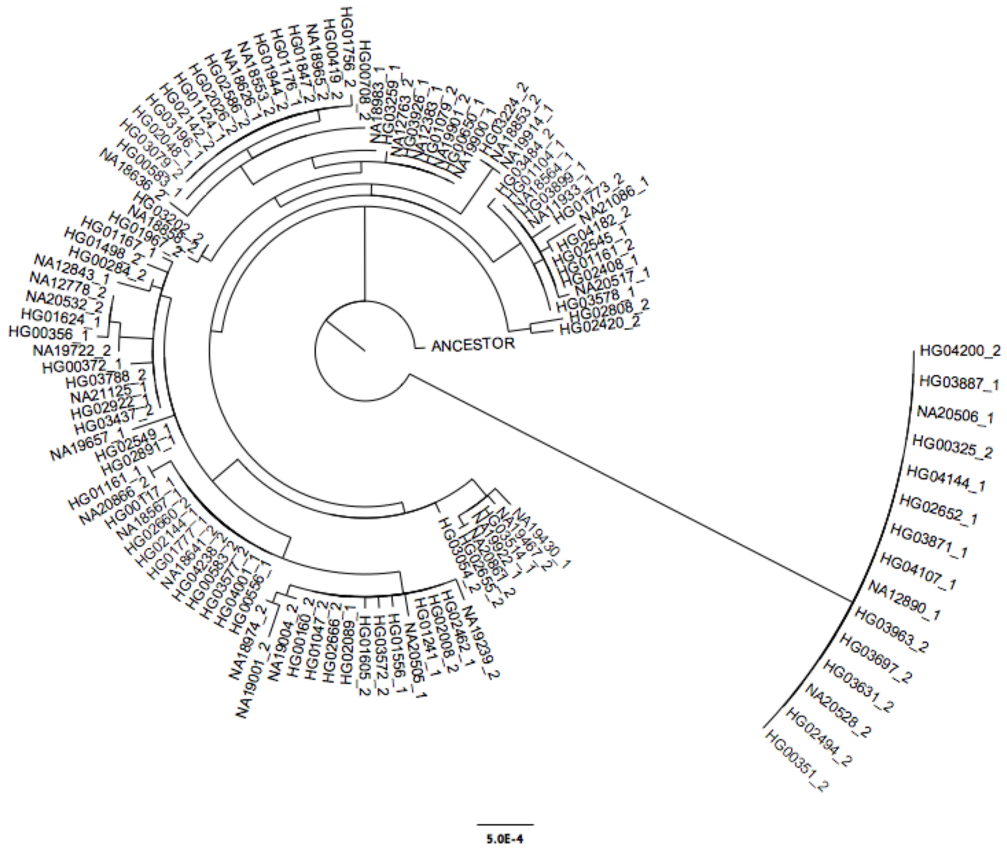

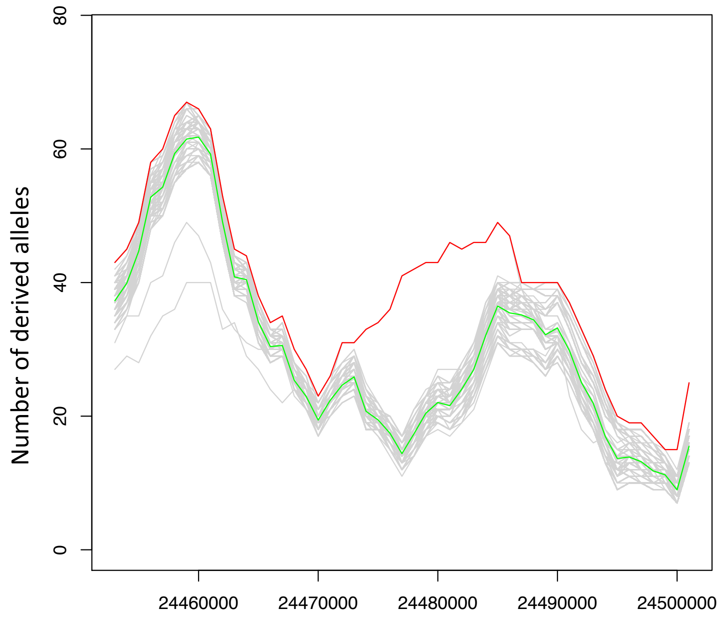

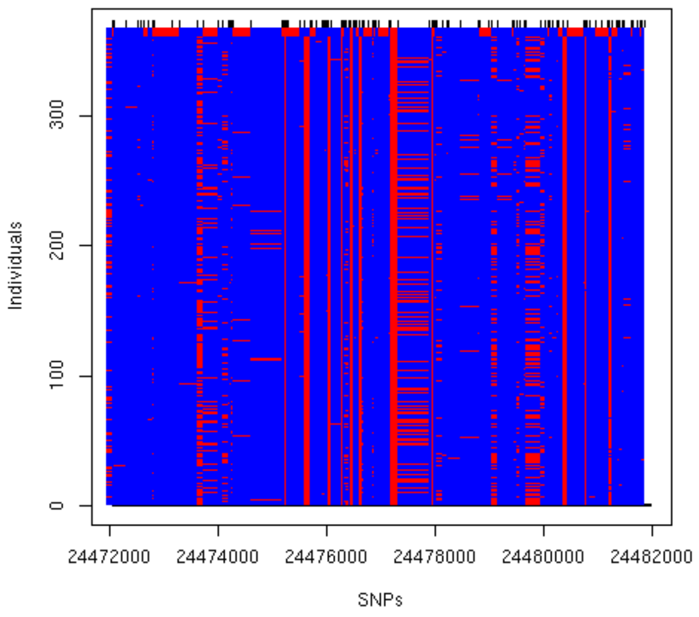

Locus: Chr12, 87Mb

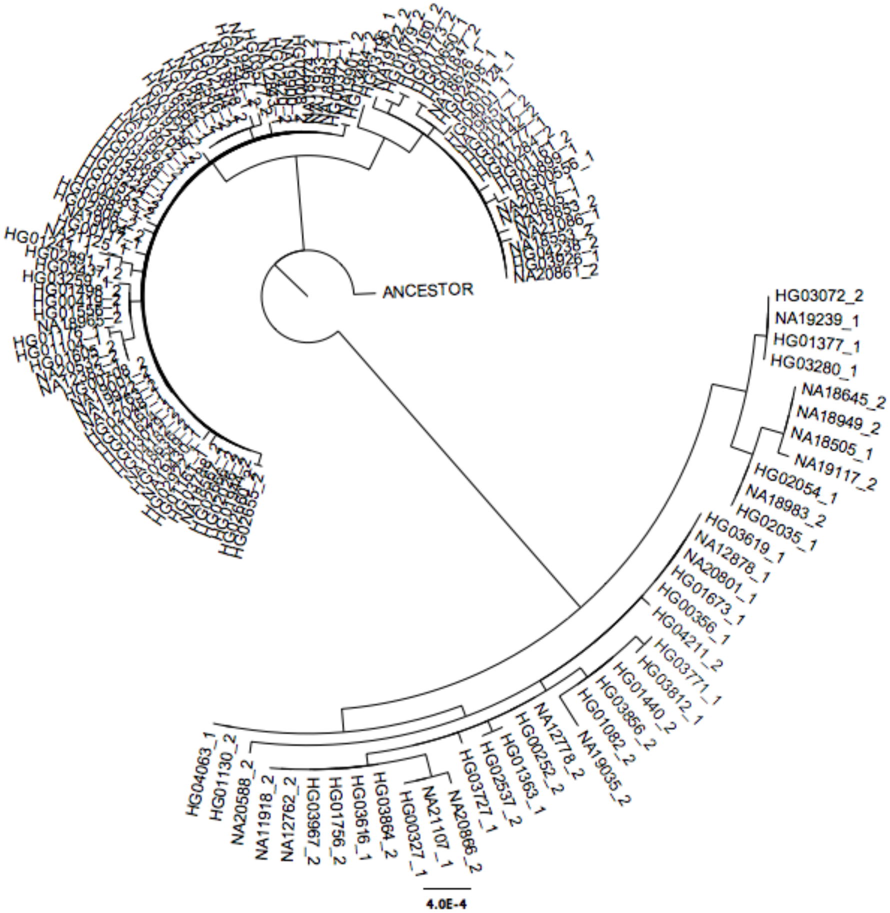

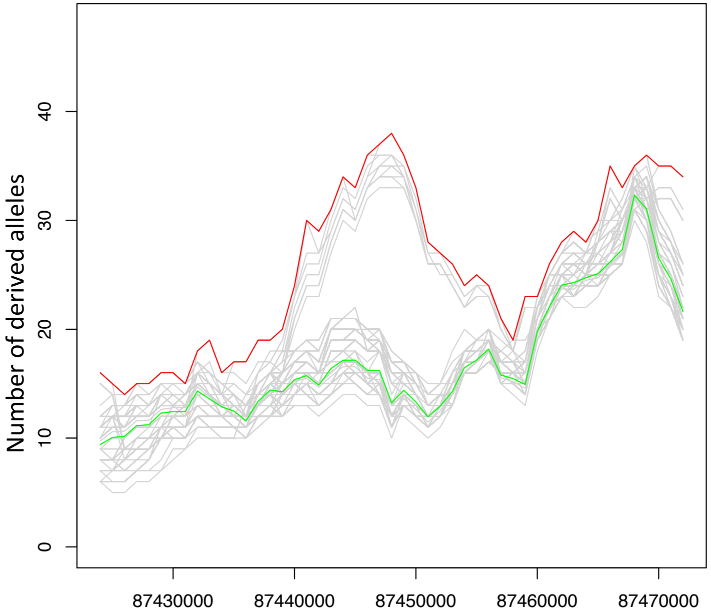

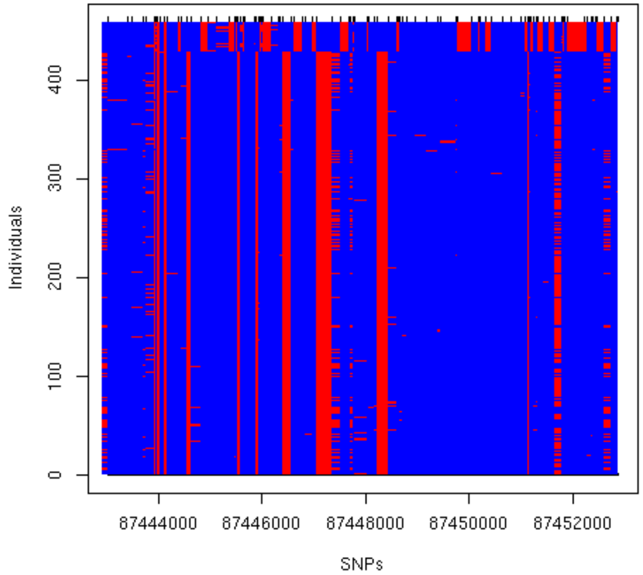

Locus: Chr1, 193Mb

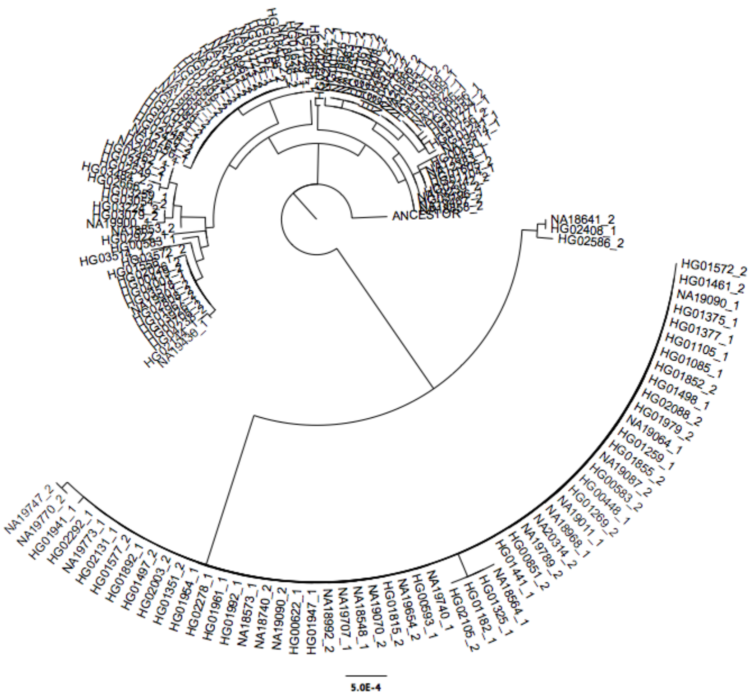

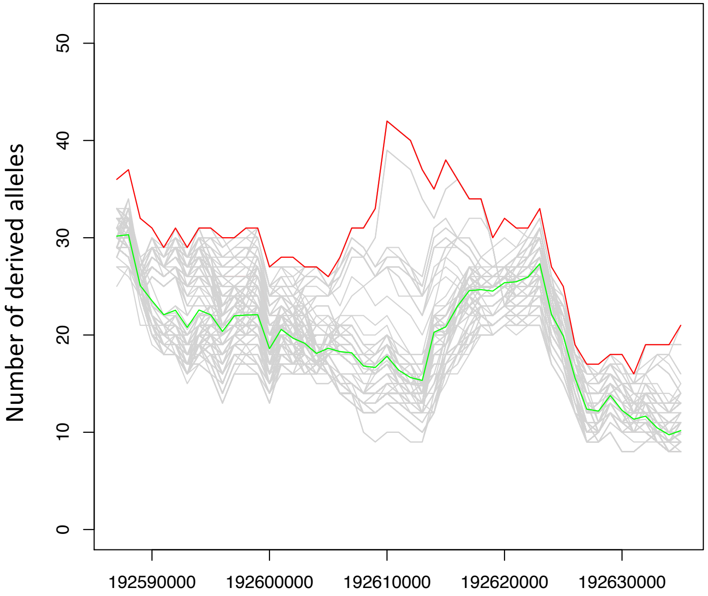

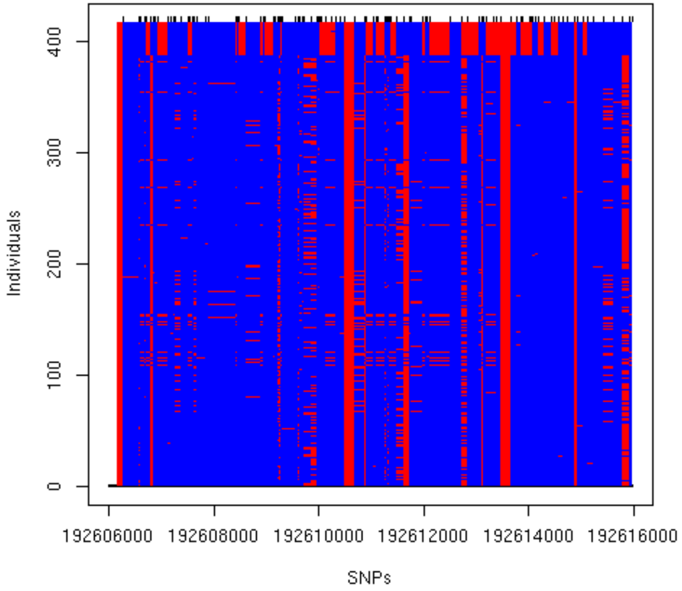

Locus: Chr12, 100Mb

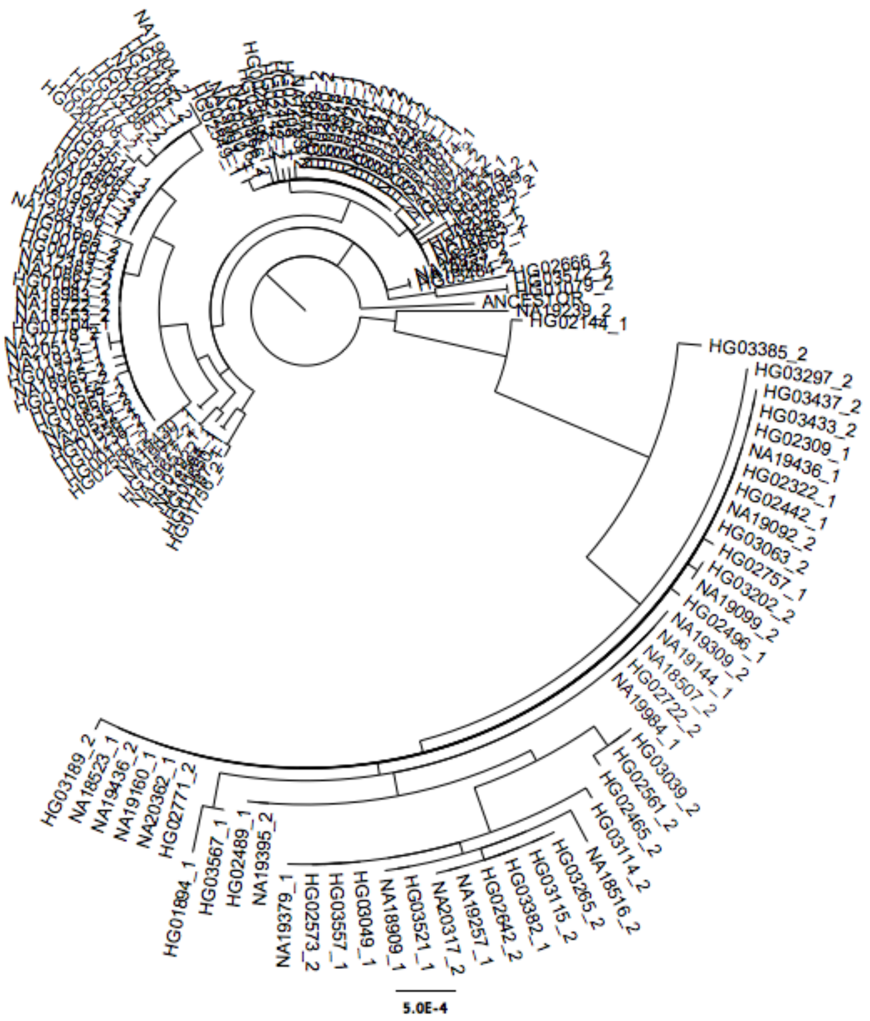

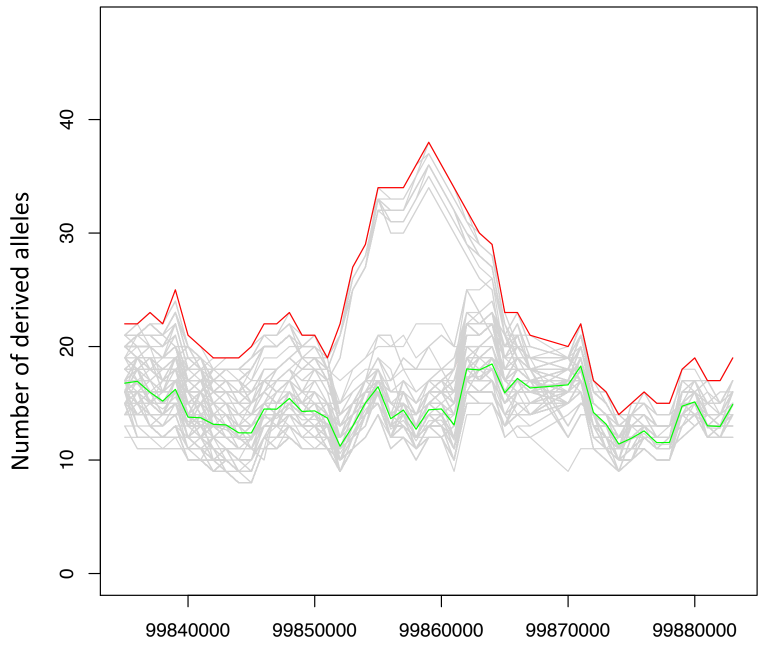

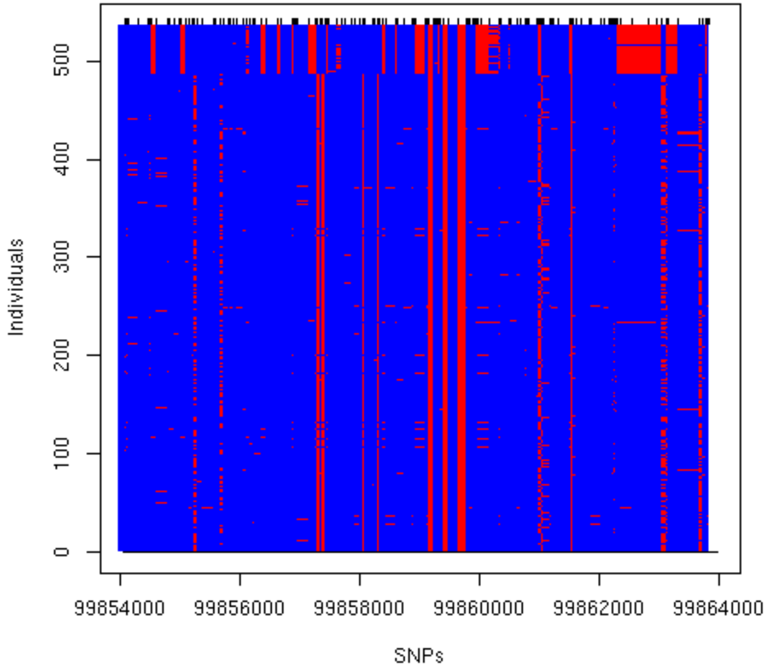

Locus: Chr17, 15.9Mb

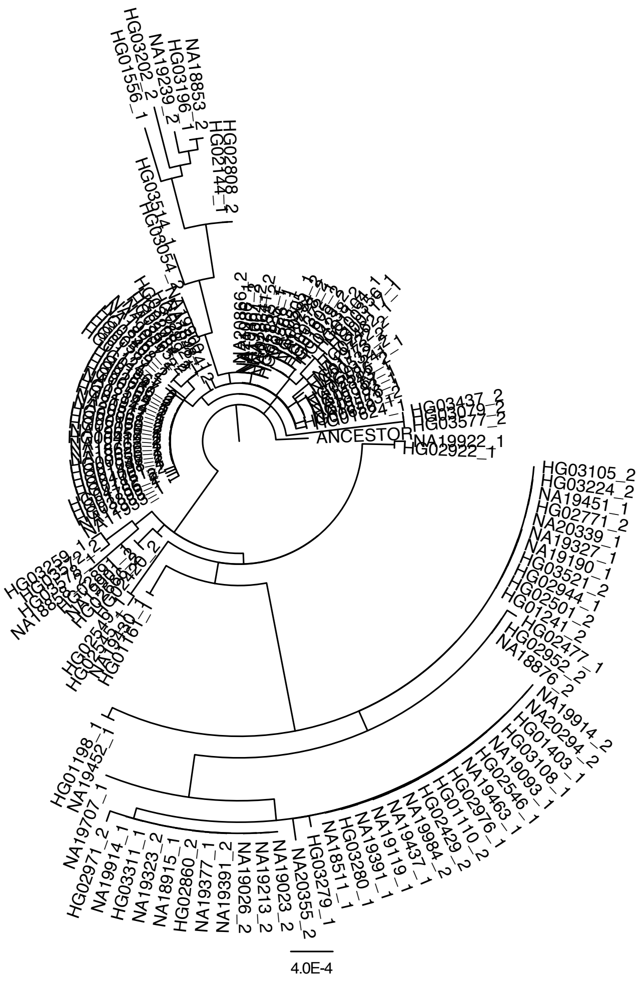

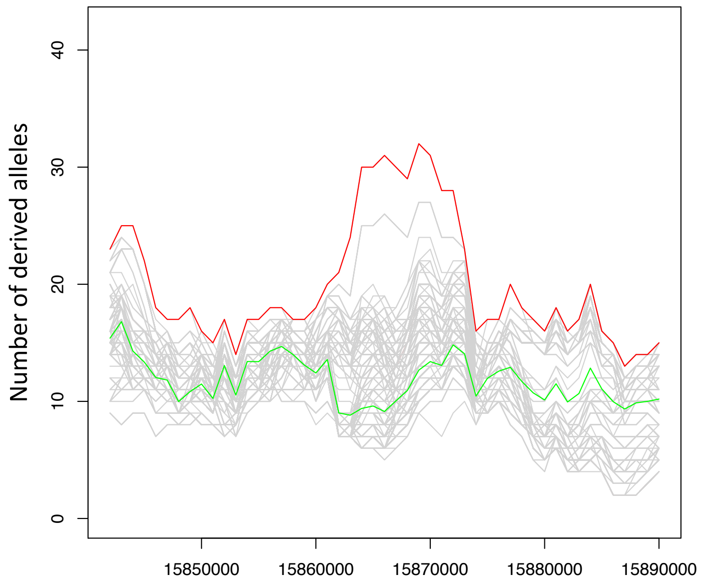

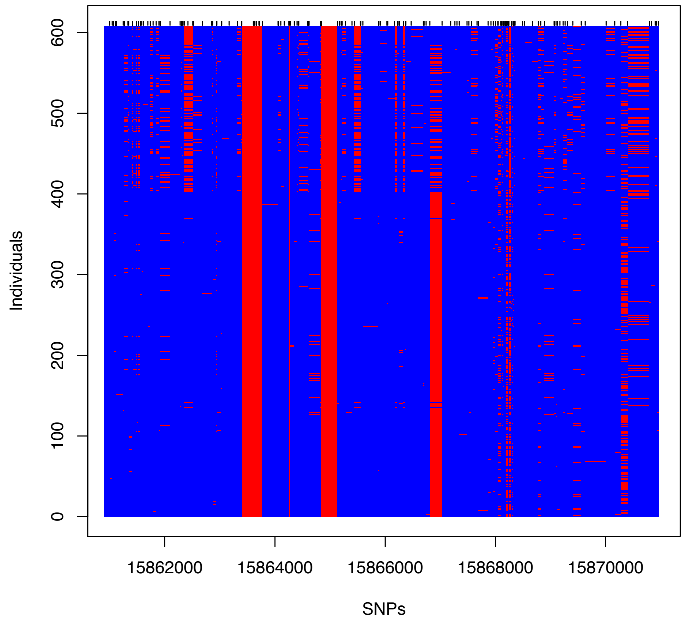

Locus: Chr14, 90Mb

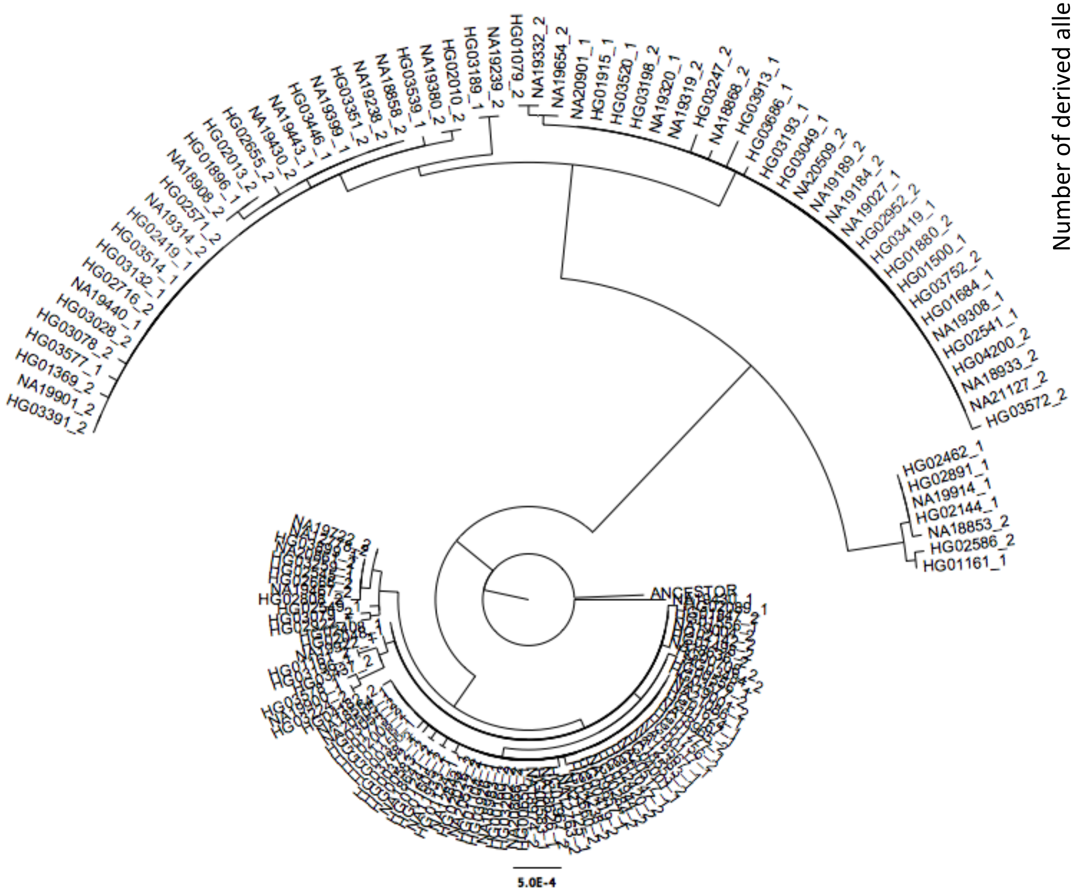

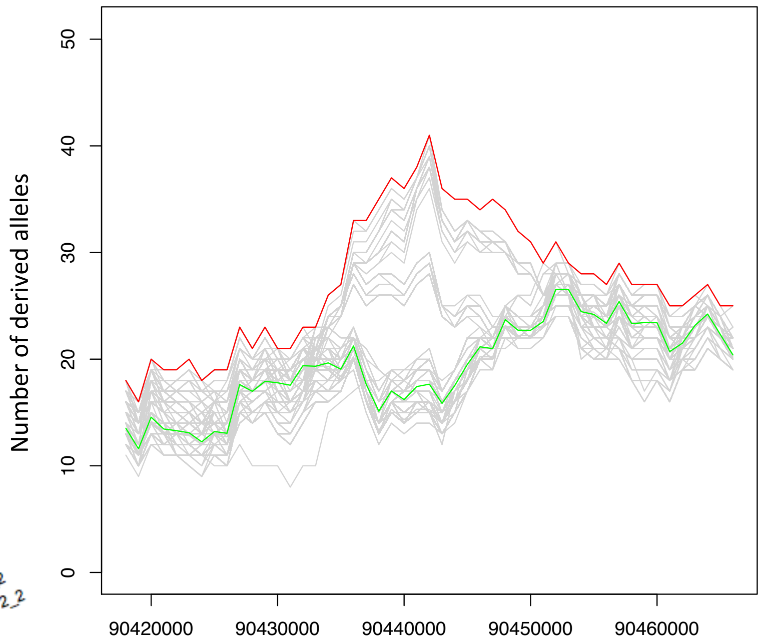

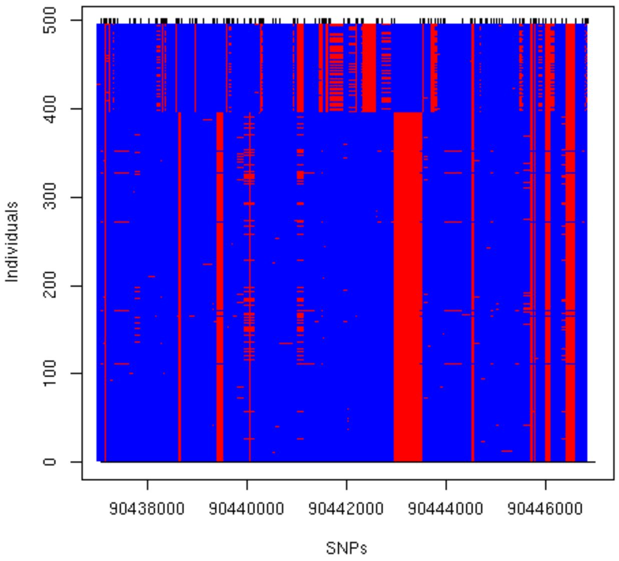

Locus: Chr6, 130.9Mb

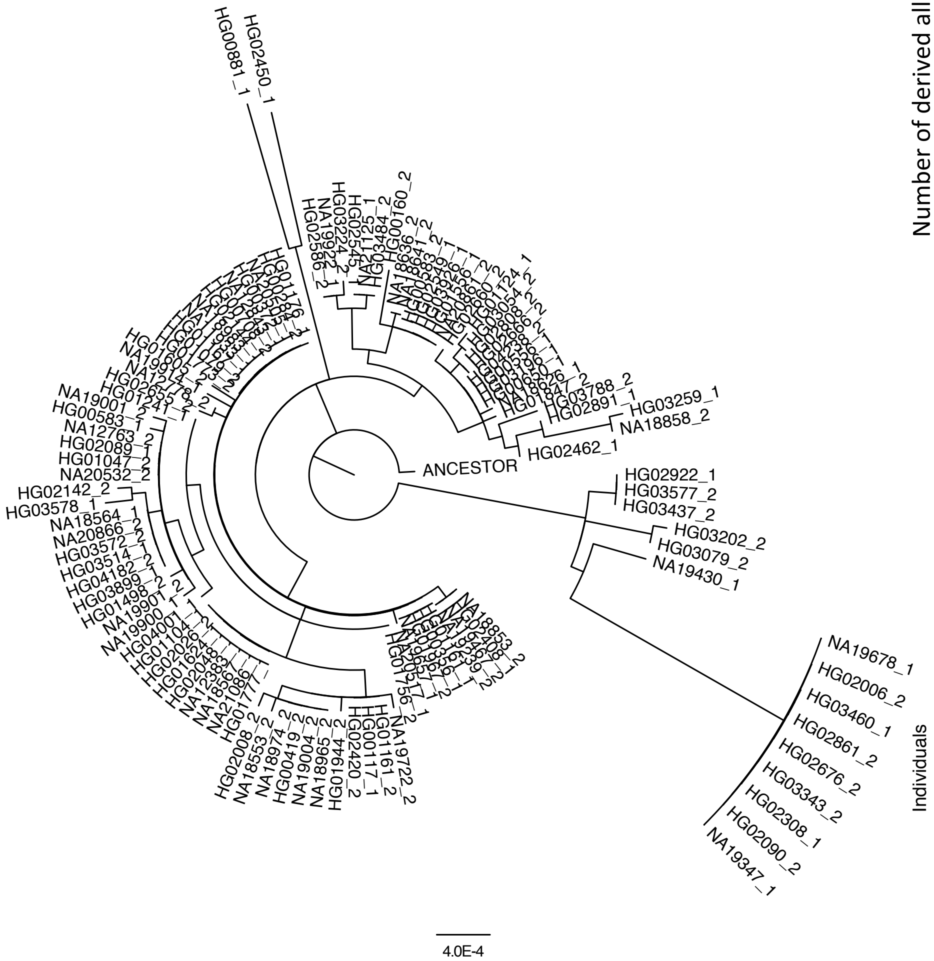

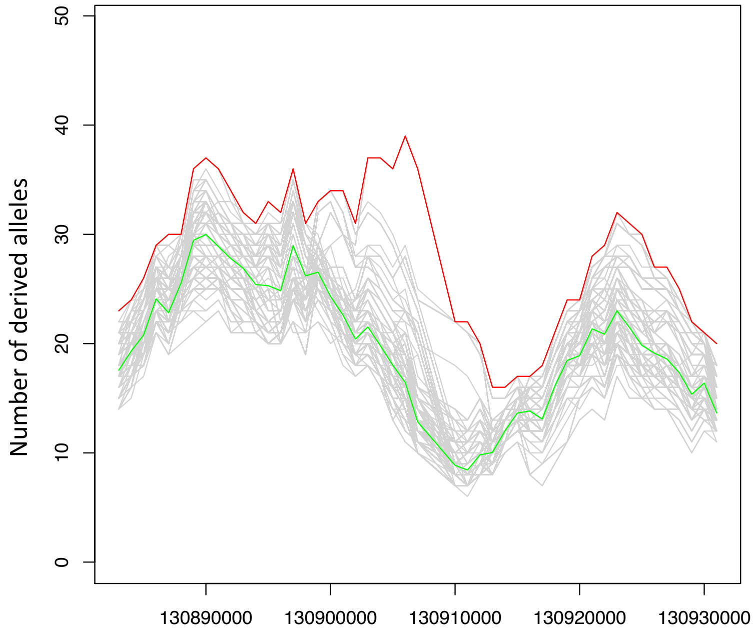

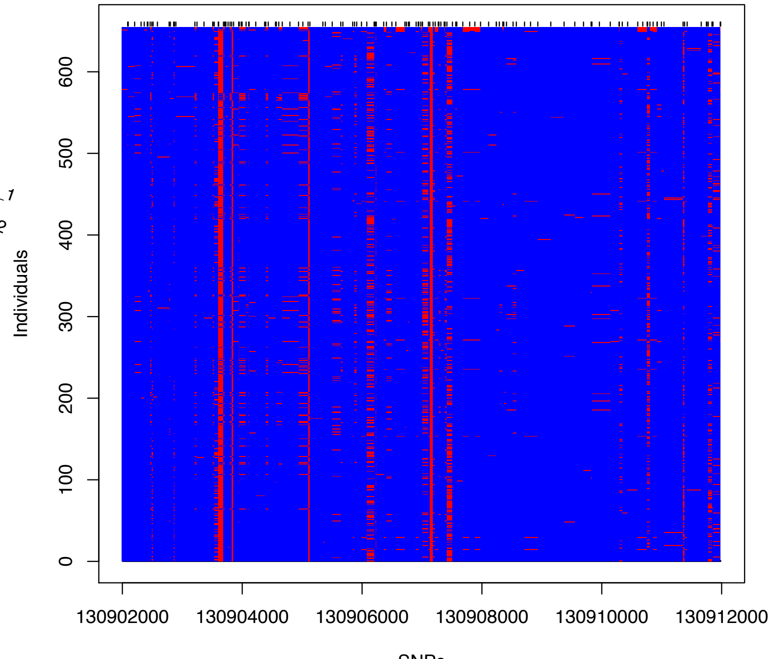

Locus: Chr8, 42Mb

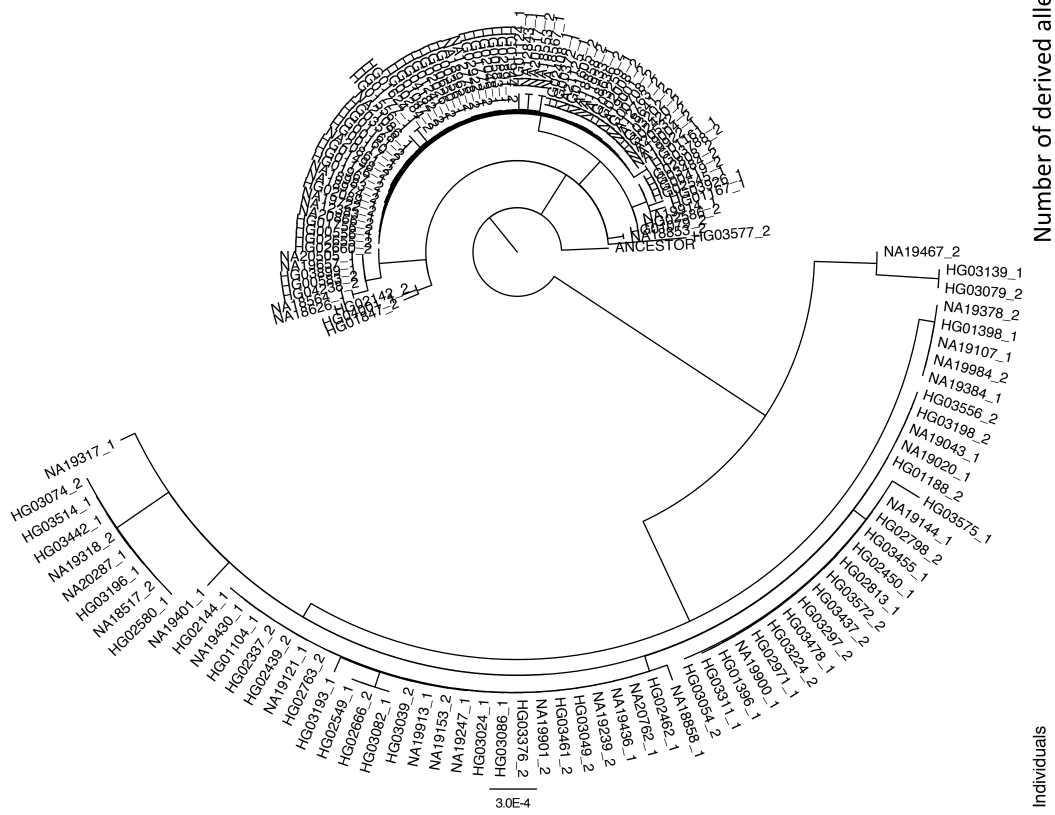

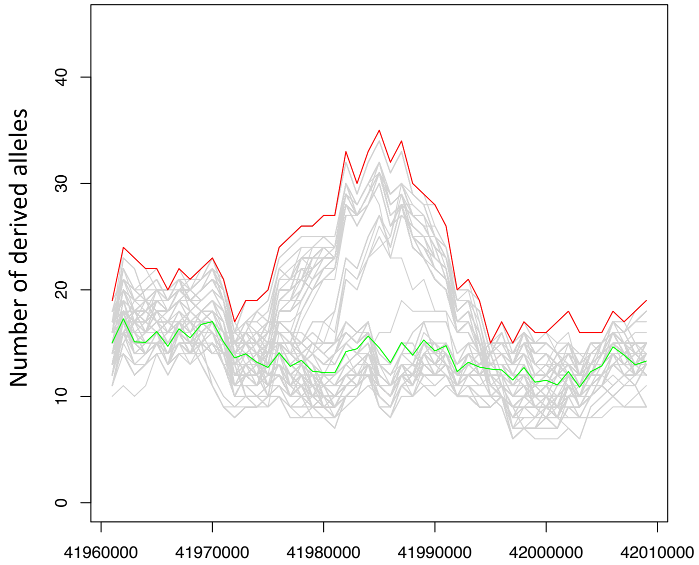

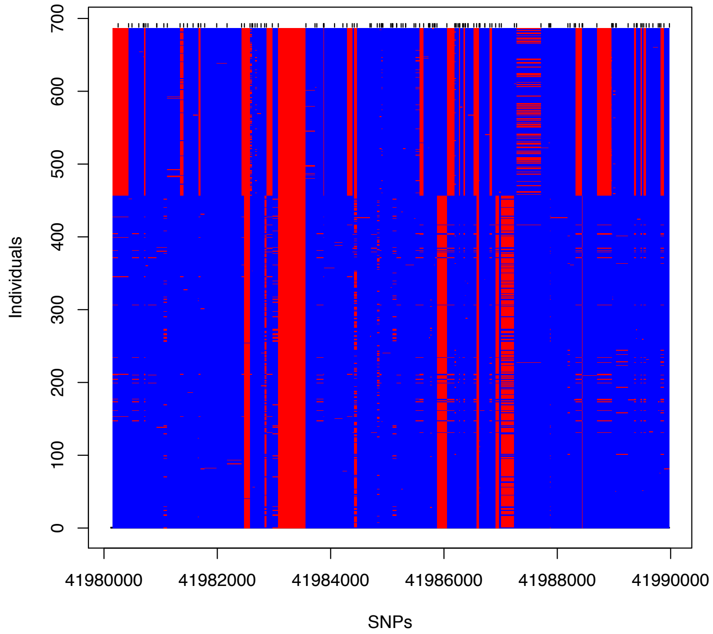

Locus: Chr10, 25Mb

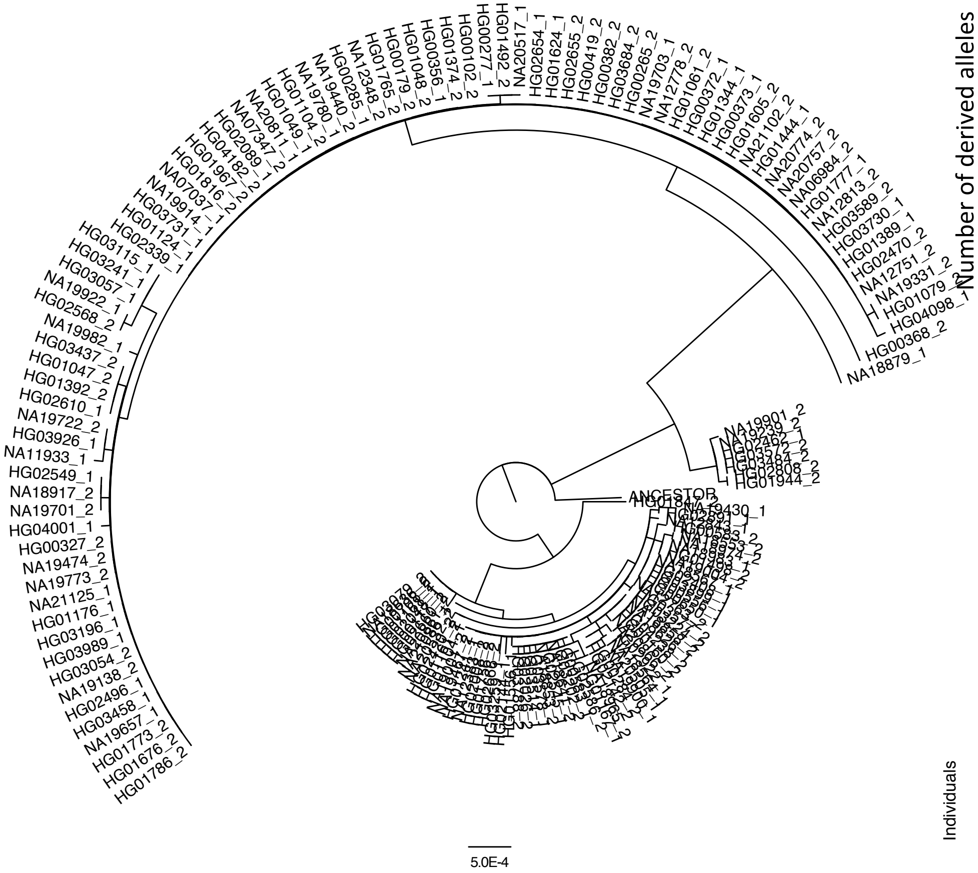

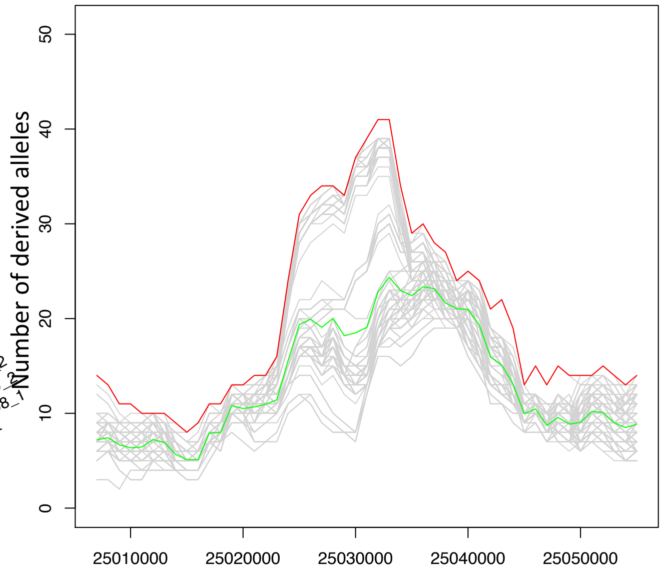

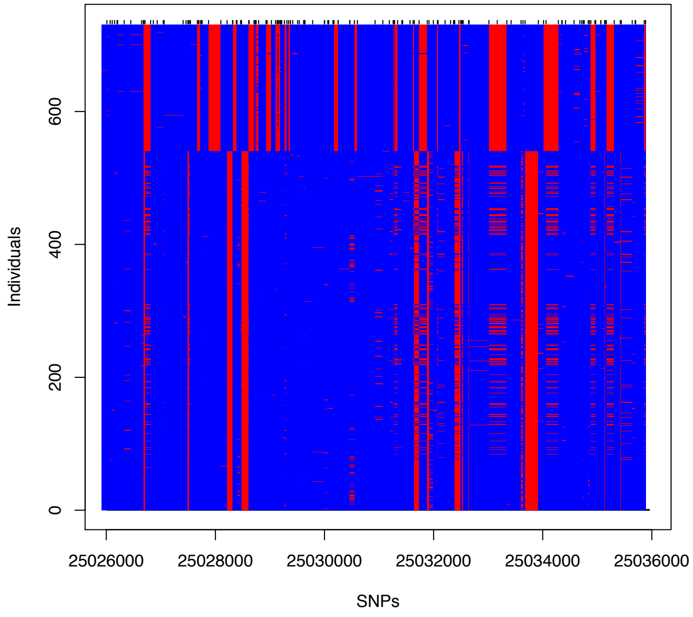

Locus: Chr21, 24.1Mb

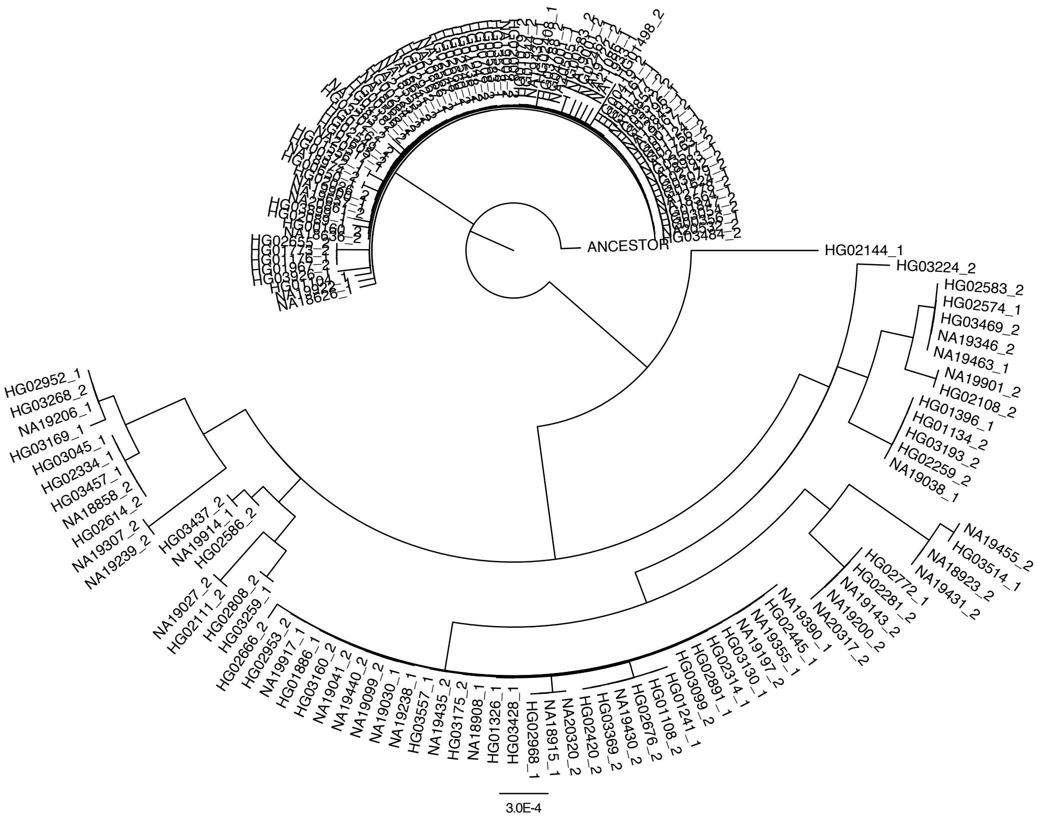

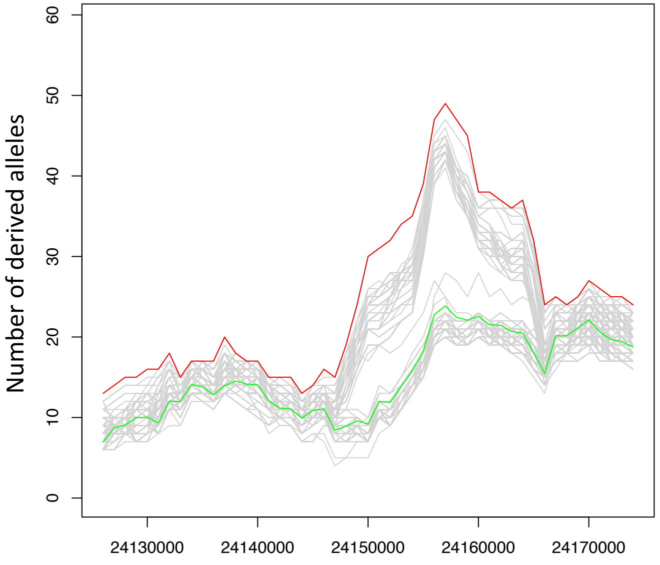

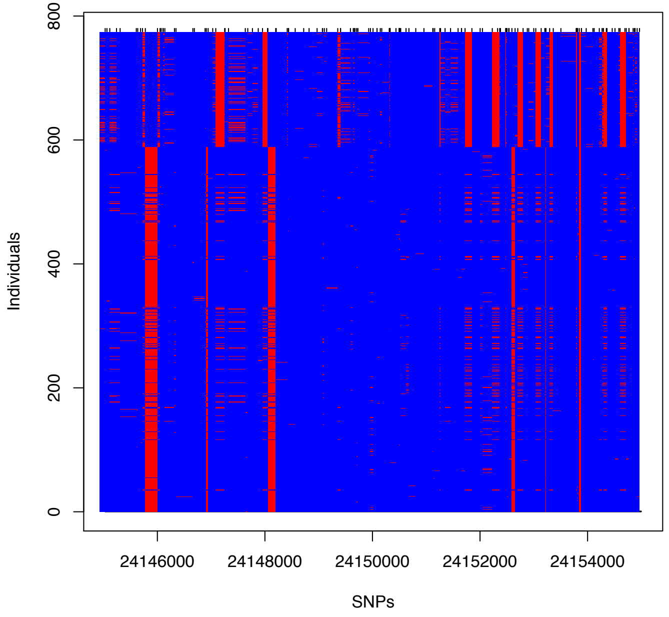

Locus: Chr12, 44.6Mb

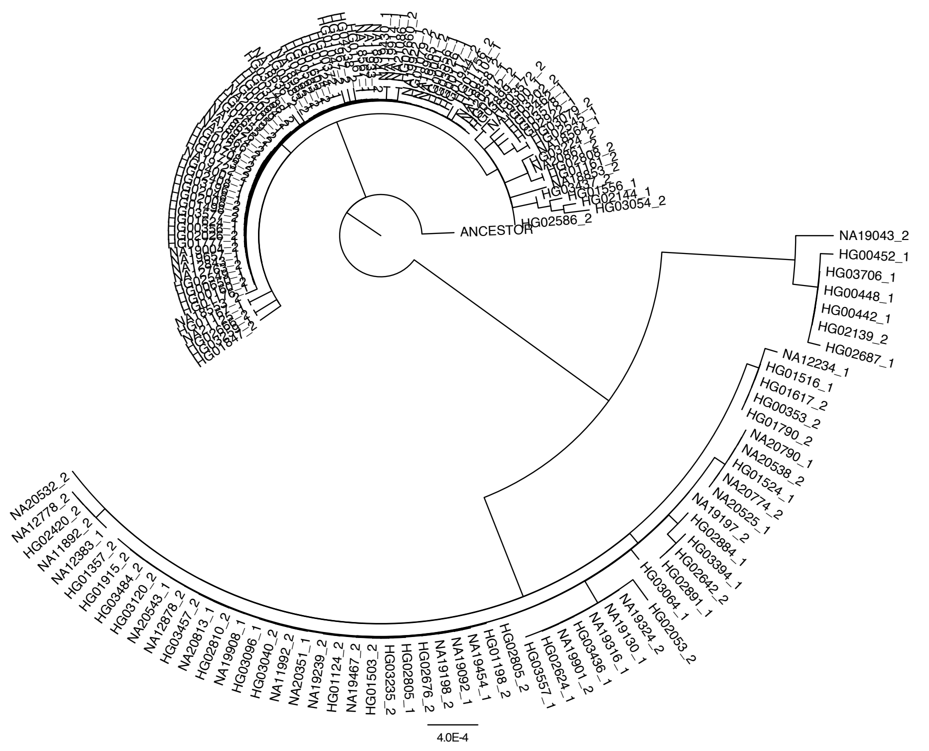

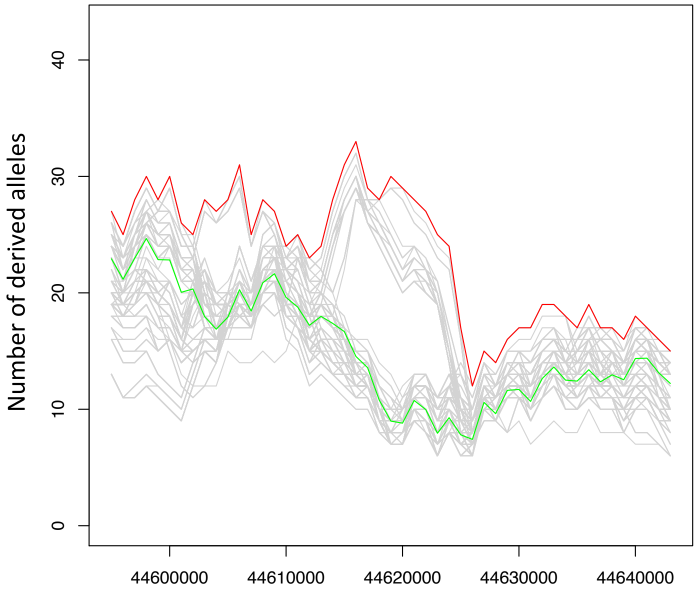

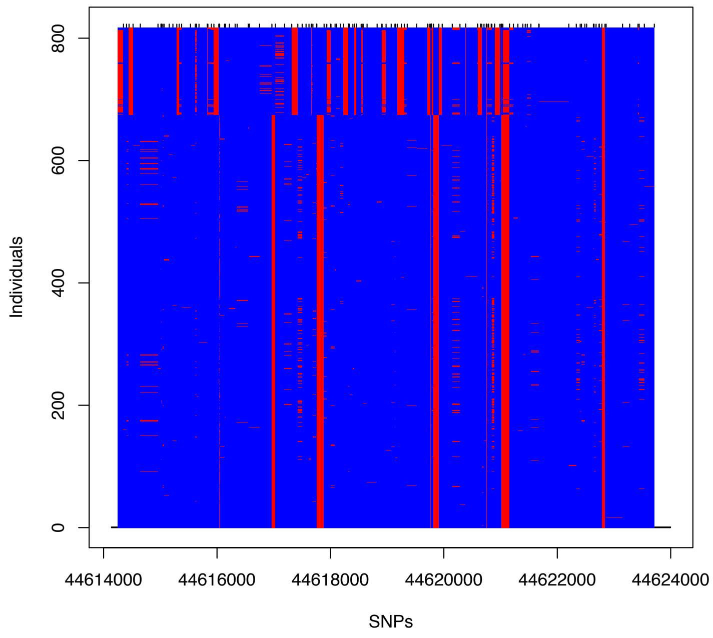

Locus: Chr5, 17.3Mb

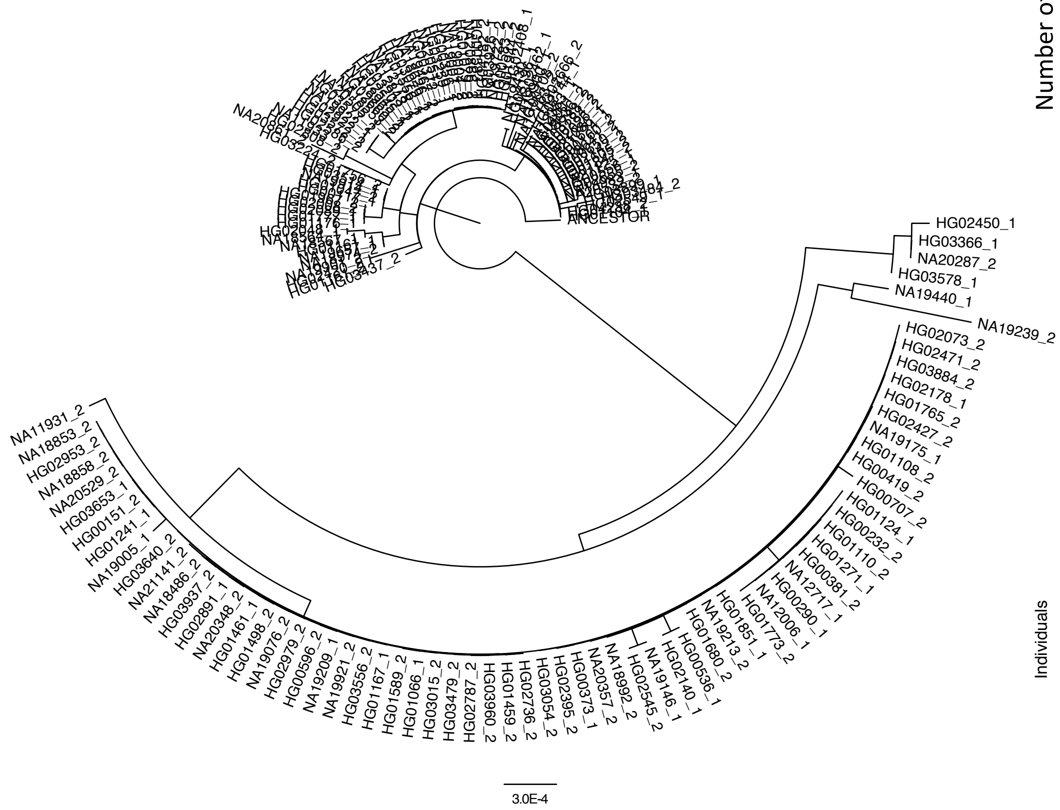

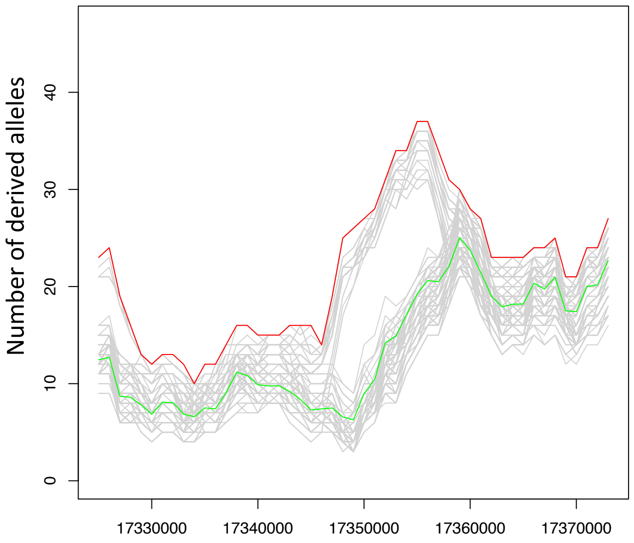

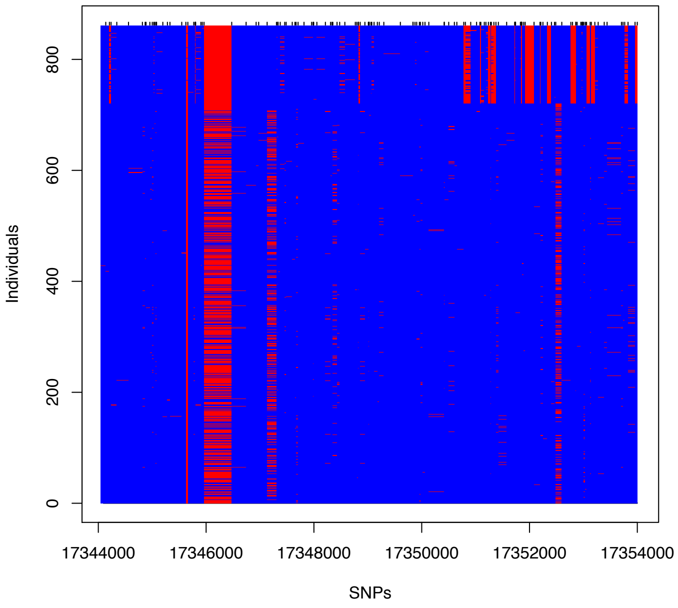

Supplement: S3 Fig — For each candidate mutator locus in Table 1 we show a phylogenetic tree (left), constructed from a 10 Kb window overlapping the peak. Due to the difficulty displaying all 5008 haplotypes from the 1000 Genomes Project we sampled 100 haplotypes at random and supplemented these with up to approximately 50 of the highly derived haplotypes, again at random. Bottom right panels illustrate for this reduced set of haplotypes the number of derived (red) and ancestral alleles (blue) in each haplotype across the 10Kb window. Each haplotype is represented as a row on the plot and the haplotypes with the largest number of derived alleles are grouped at the top of the plot. Tick marks indicate the positions of individual SNPs. The top right panels show the maximum (red) and interquartile mean (green) number of derived alleles, as well as the number of derived alleles on actual haplotypes (grey). (PDF) [file pgen.1006549.s003.pdf]
